# MicroRNA biogenesis and activity in plant cell dedifferentiation stimulated by cell wall removal

**DOI:** 10.1186/s12870-021-03323-9

**Published:** 2022-01-03

**Authors:** Konrad Dełeńko, Przemysław Nuc, Dawid Kubiak, Dawid Bielewicz, Jakub Dolata, Katarzyna Niedojadło, Sylwia Górka, Artur Jarmołowski, Zofia Szweykowska-Kulińska, Janusz Niedojadło

**Affiliations:** 1 Department of Cellular and Molecular Biology, Nicolaus Copernicus University, Lwowska 1, 87-100 Toruń, Poland; 2 Centre For Modern Interdisciplinary Technologies, Nicolaus Copernicus University, Wileńska 4, 87-100 Torun, Poland; 3 Department of Gene Expression, Institute of Molecular Biology and Biotechnology, Faculty of Biology, Adam Mickiewicz University, Uniwersytetu Poznańskiego 6, 61-614 Poznan, Poland; 4 Center for Advanced Technology, Adam Mickiewicz University, Uniwersytetu Poznańskiego 10, 61-614 Poznań, Poland

**Keywords:** Dedifferentiation, Protoplasts, miRNA, Dicing body, Cell cycle regulation

## Abstract

**Background:**

Despite the frequent use of protoplast-to-plant system in in vitro cultures of plants, the molecular mechanisms regulating the first and most limiting stages of this process, i.e., protoplast dedifferentiation and the first divisions leading to the formation of a microcallus, have not been elucidated.

**Results:**

In this study, we investigated the function of miRNAs in the dedifferentiation of *A. thaliana* mesophyll cells in a process stimulated by the enzymatic removal of the cell wall. Leaf cells, protoplasts and CDPs (cells derived from protoplasts) cultured for 24, 72 and 120 h (first cell division). In protoplasts, a strong decrease in the amount of AGO1 in both the nucleus and the cytoplasm, as well as dicing bodies (DBs), which are considered to be sites of miRNA biogenesis, was shown. However during CDPs division, the amounts of AGO1 and DBs strongly increased. MicroRNA transcriptome studies demonstrated that lower amount of differentially expressed miRNAs are present in protoplasts than in CDPs cultured for 120 h. Then analysis of differentially expressed miRNAs, selected pri-miRNA and mRNA targets were performed.

**Conclusion:**

This result indicates that miRNA function is not a major regulation of gene expression in the initial but in later steps of dedifferentiation during CDPs divisions. miRNAs participate in organogenesis, oxidative stress, nutrient deficiencies and cell cycle regulation in protoplasts and CDPs. The important role played by miRNAs in the process of dedifferentiation of mesophyll cells was confirmed by the increased mortality and reduced cell division of CDPs derived from mutants with defective miRNA biogenesis and miR319b expression.

**Supplementary Information:**

The online version contains supplementary material available at 10.1186/s12870-021-03323-9.

## Background

In plants, in vitro tissue or cell culture can be employed to induce organogenesis or somatic embryogenesis. Thus, whole fertile plants can be regenerated under proper culture conditions. These methods are widely employed for the rapid, commercial-scale propagation of large numbers of progeny plants that are genetically identical to the stock plant. In the first stage of each in vitro technique, reprogramming and dedifferentiation of plant cells occur [1, 2]. The dedifferentiation of cells includes reentry into the cell cycle, changes in the balance between euchromatin and heterochromatin, gradient distributions of phytohormones, hormone signal transduction and the reprogramming of gene expression [3, 4].

Protoplasts, cells that are dedifferentiated as a result of cell wall removal, are a convenient material for such studies due to the possibility of analyzing large numbers of individual cells. These cells acquire totipotency and, after being supplied with hormones, could be regenerated into fertile plants. The most dedifferentiation and reprogramming events occur within the first several days of culture [5]. One of the first changes to occur during the transition from leaf mesophyll cells to protoplasts is the decondensation of chromatin, which in *A. thaliana* and *Cucumis sativus* L. is manifested by a decrease in the number and size of chromocenters [6, 7]. This process is not accompanied by changes in epigenetic marker levels, such as DNA methylation and H3K9 dimethylation. Despite chromatin decondensation, the acquisition of totipotency by protoplasts is associated with the temporary inhibition of RNA synthesis. In *A. thaliana* protoplasts, it has been shown that the expression of only half of the differentially expressed genes (DEGs) was increased, and the total number of reads, as determined by mRNA sequencing, was the lowest of all studied stages of dedifferentiation [5]. After protoplasting, the level of the elongated form of RNA polymerase II was also strongly decreased by approximately 8.5-fold compared to mesophyll cells [8]. In the subsequent stages of dedifferentiation, intensive eradication of 25S rRNA and poly(A) RNA transcripts from the cytoplasm also takes place. This phenomenon - known as cytoplasm cleaning - removes rRNA and coding RNA that were derived from mesophyll cells. Hence, new mRNA transcripts enabling a cell fate switch may be translated into newly formed ribosomes [8]. However, the contribution of posttranscriptional events to mRNA removal in the initial steps of dedifferentiation has not been elucidated to date.

The process of cell reprogramming requires many modifications at the level of gene expression. In this process, miRNAs are molecules with very high potential because they regulate target genes at the transcriptional level and posttranscriptional level via two major mechanisms: transcript cleavage and translation repression [9]. In plants, after transcription, pri-miRNA is completely processed in the nucleus by a complex called Microprocessor, which, in *Arabidopsis*, consists of three core proteins: the RNase type III enzyme Dicer-like 1 (DCL1), the zinc finger protein Serrate (SE), and the double-stranded RNA binding protein Hyponastic Leaves 1 (HYL1) [10]. The first cut on pri-mRNA by DCL1 is 15–17 nt away from the base of the stem or a bulge or unstructured region within the loop-distal stem. After further cleavage, a 21-nt miRNA/miRNA* duplex is produced [11–14]. DICER-LIKE 1 (DCL1) proceeds then with three additional cuts until the mature miRNA is released [15]. The strong accumulation of Microprocessor proteins and the two miRNA precursors, i.e., pri-miR163 and pri-miR173, have been demonstrated in nuclear D-bodies (DBs); therefore, these structures are commonly recognized as pri-miRNA processing sites [16–18]. Currently, many other proteins are known, as well as their posttranslational modifications by the Microprocessor, which affect the level of pri-miRNA, miRNA and sometimes DB assembly/disassembly. For example, in the MOS2 mutant, DB identified by HYL1 were not observed [19]. The association between pri-miRNAs and HYL1 was greatly reduced in *mos2–2,* and decreased levels of mature miRNAs were observed. Another protein regulating DB formation is CPL-1 phosphatase, which dephosphorylates Ser5 of the C-terminal domain (CTD) of the largest subunit of RNA POLYMERASE II. CPL1 interacts with SE to dephosphorylate HYL1. Hypophosphorylated HYL1 enables the correct selection of strands from the miRNA:miRNA duplex * and is responsible for the assembly of D-bodies [20]. In turn, mutants of two Elongator subunits (*elp2–2* and *elp5–1*) have exhibited disrupted DCL1 localization and a reduced number of D-bodies [21]. Complex Elongator-DLC1 associates with chromatin and is localized in D-bodies. The knockout of Elongator subunits results in reduced levels of mature miRNA and pri-miRNAs. These findings suggest a connection between transcription and processing of pri-miRNA.

It is known that miRNAs function in both the nucleus and the cytoplasm. In the nucleus, some of the miRNAs bound to AGO1 associate with chromatin, regulating its activity [22]. Immunoprecipitation experiments have indicated that the nuclear pool of AGO1 increased with the initiation and elongation RNA polymerase II and SWI/SNF complex subunits (SWI3B, SWI3D and BSH) [22]. Moreover, the association of AGO1 with chromatin was shown to be impaired in the HYL1-knockout mutant (*hyl1–2*) or upon alteration of the phosphorylation status of HYL1. The results indicated that various stimuli, including plant hormones and stresses, specifically trigger AGO1 binding to stimulus-responsive genes. AGO1 has also been shown to block miR161 and miR173 transcription. AGO1 enrichment on these two MIR genes was observed upon salt stress treatment and correlated with decreased RNA POLYMERASE II occupancy. It has been shown that the presence of AGO1 on the gene body causes RNA POLYMERASE II to pause or terminate transcription prematurely [23, 24].

In this study, we determined the roles played by miRNAs in the dedifferentiation of *A. thaliana* mesophyll cells. The effect of disturbances in miRNA biogenesis on this process was analyzed. The biogenesis of miRNA and RISC was studied by qPCR pri-miRNA and miRNA, as well as by the localization and quantity of AGO1 and D-bodies. This research identified which miRNAs are involved in dedifferentiation and may influence the first division of CDPs (cells derived from protoplasts).

## Results

### Viability and division rate of protoplasts derived from wt and *dcl-1* mutant

**Fig. 1:**
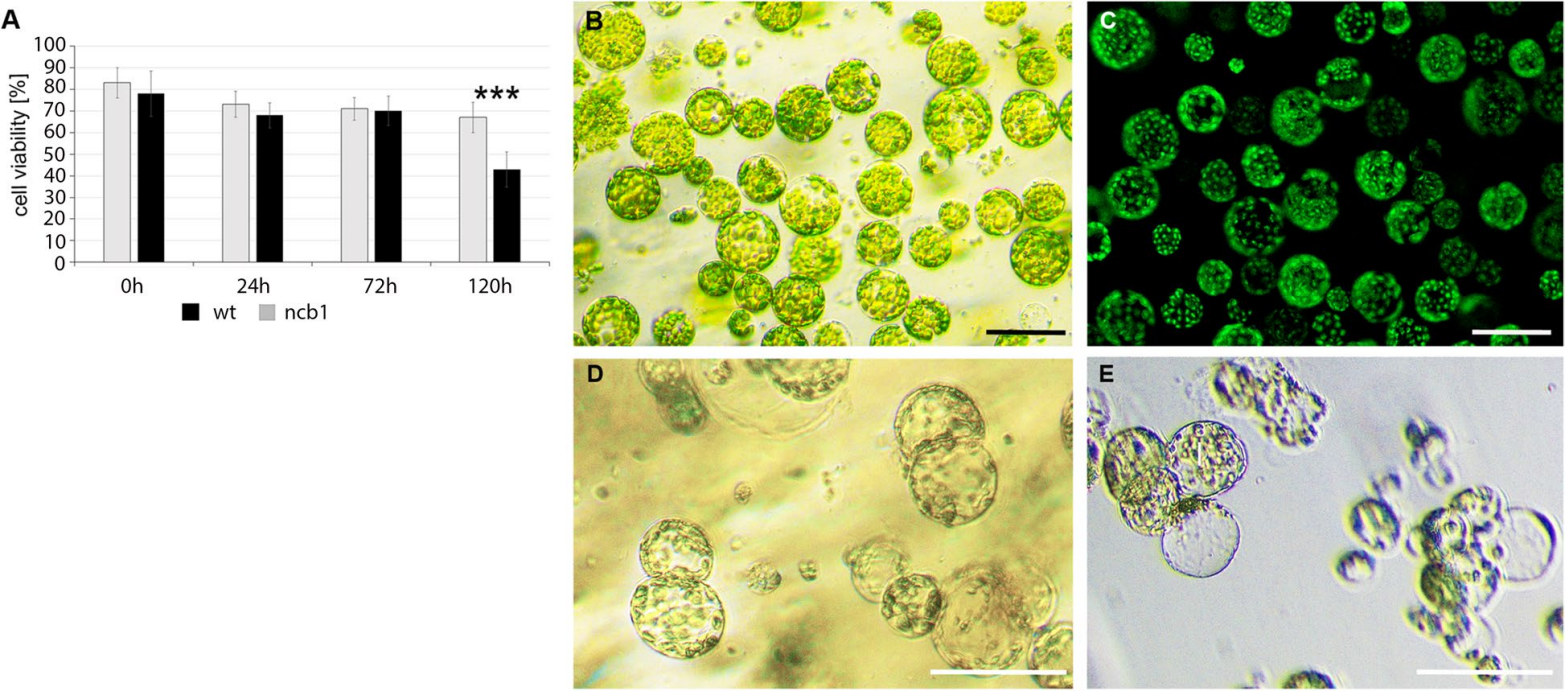
(**A**) Viability of isolated protoplasts (0 h) and CDPs cultured for 24 h, 72 h, and 120 h (dividing cells) obtained from wt and *dcl1–9* plants based on a fluorescein diacetate assay (FDA). *** indicates a significant difference between groups with *P* < 0.001, Student’s t-test, α = 0.05. Error bars represent standard deviation. (**B**) Isolated protoplasts, (**C**) the same cells stained with fluorescein diacetate (FDA). (**D**) Divided CDPs after 120 h of culture and (**E**) single microcalli. Bar 100 μm

Mesophyll cell dedifferentiation was stimulated by removal of the cell wall. To this end, slow degradation of cell walls was carried out for 14 h. The 75–90% protoplasts were determined to be viable in all repetitions based on a fluorescein diacetate assay (FDA) (Fig. 1A-C). The first cell divisions were observed after 72 h of culture of protoplasts (cells derived from protoplasts; CDP). After 120 h of culture, 25–35% of CDPs were divided, and single microcalli were visible (Fig. 1D, E, Supplementary Data Fig. 1). Protoplasts of mesophyll (0 h) and CDPs (cultured for 24, 72 and 120 h) were used to study the function of miRNA in the dedifferentiation process. Initially, the survival and number of division of cells derived from wt and mutant *dcl1–9* (which is defective in miRNA biogenesis) were analyzed. No differences in morphology, including size, staining and distribution of chloroplasts and vacuoles, between wt and *dcl1–9* protoplasts were observed (Supplementary Data Fig. 2). After protoplasting, the viable cells of both variants of plants were similar until 72 h of culture (Fig. 1A). A difference in the survival of wt and *dcl1–9* cells cultured for 120 h was noted. The percentages of viable cells were 67 and 43% for wt and *dcl1–9*, respectively. In addition, only 0.5–1% dividing *dcl1–9* cells were observed compared with 25–35% dividing wt cells. These results indicate that miRNA-dependent regulation of gene expression is important for dedifferentiation and the first divisions.

### miRNA activity based on the analysis of the quantity and distribution of D-bodies and AGO1

**Fig. 2:**
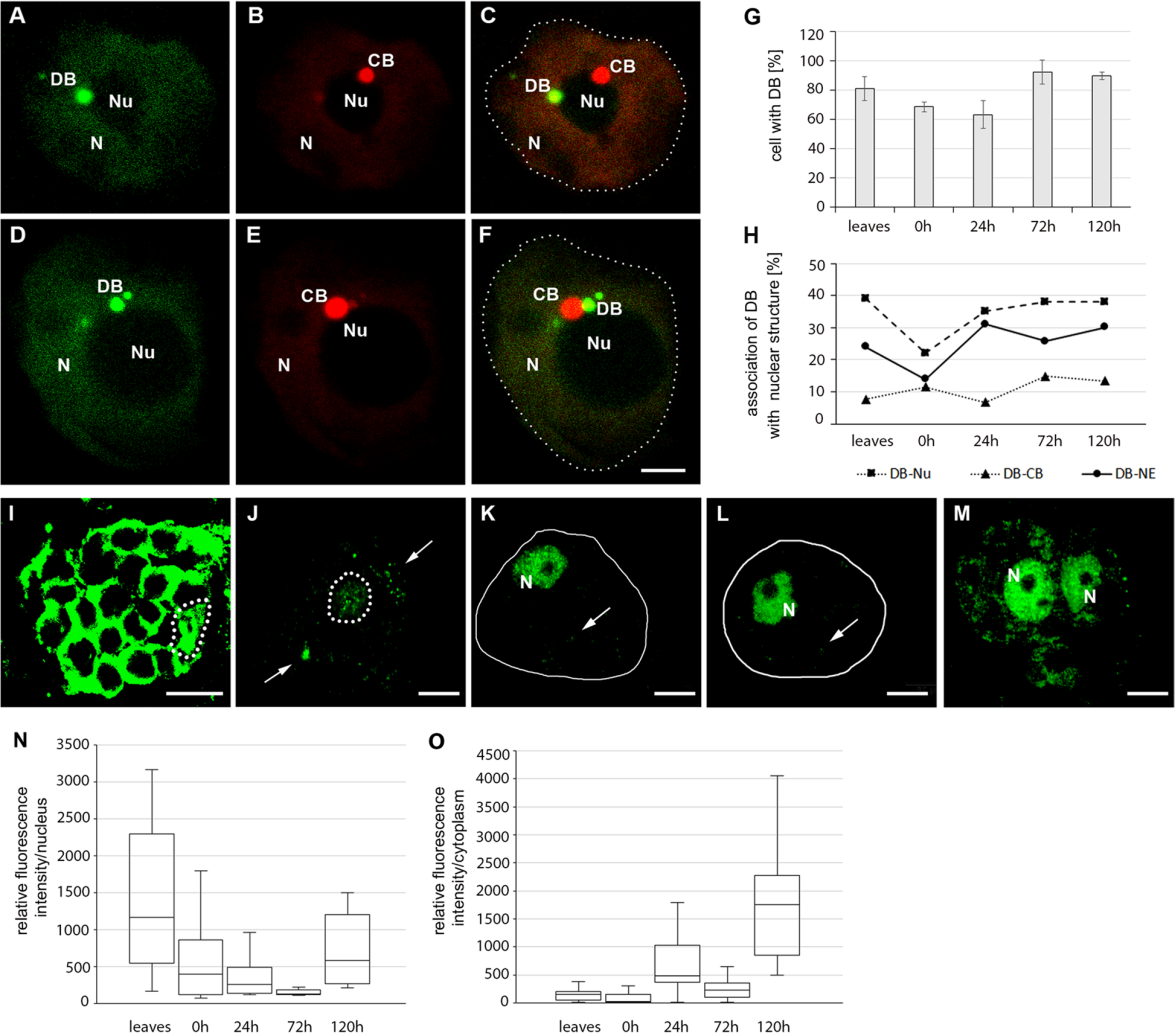
Quantity and distribution of D-bodies and AGO1. Localization of Hyl1-YFP and U2 snRNA in protoplasts (**A**-**C**) and CDPs cultured for 120 h (**D**-**F**) by confocal microscopy. (**A**-**C**) Dicer bodies (DB) and Cajal bodies (CB) occur separately at the periphery of the nucleolus (Nu) in protoplasts. In the CDP cells cultured for 120 h, two DBs are in the nucleus (N). Of these, 1 is strongly associated with CB. In C, F dotted line indicate nucleus (**G**). Percentage of isolated protoplasts (0 h) and CDPs cultured for 24 h, 72 h, and 120 h (dividing cells) with DBs; error bars represent standard deviation. (**H**) Percentage of DBs associated with nuclear structures in mesophilic cells and subsequent stages of their dedifferentiation (Nu-nucleolus, CB-Cajal body, NE-nuclear envelope). Localization of AGO1 in mesophyll cells (**I**), isolated protoplasts (**J**), and CDPs cultured for 24 h (**K**), 72 h (**L**) and 120 h (**M**) under a confocal microscope. Dotted line – nucleus, continuous line – cell, N - nucleus, arrow – clusters of AGO1 in the cytoplasm. Bar 5 μm. The relative fluorescence intensity of AGO1 in the cytoplasm (**N**) and nucleus (**O**) in mesophyll cells and during dedifferentiation

The DB (dicing body) is a structure involved in miRNA biogenesis. HYL1 detection was used to visualize DBs. To study the dynamics and intensity of miRNA processing, the quantity and distribution of DBs in the nucleus were examined. To this end, protoplasts derived from *A. thaliana* HYL1-YFP were isolated and cultured. In mesophyll cells and during dedifferentiation, the HYL1 protein was primarily observed in the nucleus and accumulated in the DBs (Fig. 2A-F). Next, the percentage of cells containing DBs (Fig. 2G) and their quantity in the nucleus were analyzed (Supplementary Data Fig. 3). DBs were identified in over 81% of mesophyll cells, while a strong decrease in the number of DBs was observed after cell wall removal. Only 62% of CDP cultured 24 h nuclei contained DBs (Fig. 2G). In subsequent stages of culture, the quantity of DBs in cells increased and was comparable to that observed in leaf mesophyll cells. The highest percentage of CDPs with DBs (92.5%) was observed after culturing for 72 h (Fig. 2G). At all studied stages, nuclei with one DB were most frequently observed, and the number of such cells did not change significantly during dedifferentiation (from 37 to 41.83%) (Supplementary Data Fig. 3). On the other hand, the number of nuclei with more DBs changed significantly. There were no nuclei with five or more DBs in protoplasts and CDPs cultured for 24 h, while the number of nuclei with 4 and 3 DB was the lowest of all analyzed stages. However, during the start of cell division, not only did the total number of DBs increase, but cells containing 6, 7 and even 8 DB were also were observed for the first time (Supplementary Data Fig. 3). The quantity of DBs was strongly correlated with the level of transcription during dedifferentiation. We have previously shown the lowest amount of elongation from RNA polymerase II in protoplasts, which subsequently increased in successive stages [8]. This finding indicates that DBs may be a highly transcription-dependent structure. Our results suggest a decrease in miRNA biogenesis in protoplasts and a significant increase in CDPs cultured for 72 h and 120 h.

The localization of structures in the nucleus is often related to their function. The association of CBs (Cajal bodies) with the nucleolus suggested the involvement of this structure in rRNA metabolism. Similarly, the movement of CBs between the nuclear envelope and the nucleolus suggested a transport function [25]. Given these findings, the association of DBs with the nuclear envelope, Cajal body and nucleoli was investigated. DBs were most often located in close proximity to the nucleolus (Fig. 2A-F, H). The stress of cell wall removal and the decrease in transcription shown by Dełeńko et al. [8] was observed to result in significant DB detachment from the nucleolus. Similar trends were demonstrated at the second location, i.e., the in association with the nuclear envelope (Fig. 2H). The simultaneous location of HYL1 and U2 snRNA (plant marker of CB) enables the analysis of spatial relationships between CBs and DBs (Fig. 2A-F). The association of both structures is the least common and is observed in between 7 and 14% of cells during dedifferentiation (Fig. 2H). An increase in DBs associated with the three studied nuclear structures has been reported in dividing cells.

To determine the activity of miRNAs, we studied the localization and level of AGO1 (Fig. 2I-O), which is an element of Microprocessor and RISC in the nucleus and cytoplasm, respectively. In the cytoplasm of leaf mesophyll cells, AGO1 was observed in the areas between chloroplasts (Fig. 2I). In the protoplasts, there was a strong reduction in the level of this protein (Fig. 2N) and AGO1 was observed in the individual clusters (Fig. 2J). In the cytoplasm of dividing cells (120 h), the amount of AGO1 significantly increased and reached the highest value in dedifferentiating cells (Fig. 2M, N). This result suggests a decrease in RISC activity after protoplastization followed by an increase during cell division.

In the nucleus, the level of AGO1 decreased in protoplasts (Fig. 2O), and proteins formed numerous individual speckles (Fig. 2J). In the subsequent studied stages, the AGO1 level increased, and a homogeneous distribution pattern of varying intensity was observed except for nucleolus (Fig. 2K-M). In cells cultured for 120 h, the AGO1 signal increased more than twenty-fold compared to that observed in mesophyll cells (Fig. 2O). This finding suggests that nuclear RISCs may play an important role in the dedifferentiation process because their level increases significantly in dividing cells.

### Changes in miRNA expression during protoplastization and first cell division

**Fig. 3:**
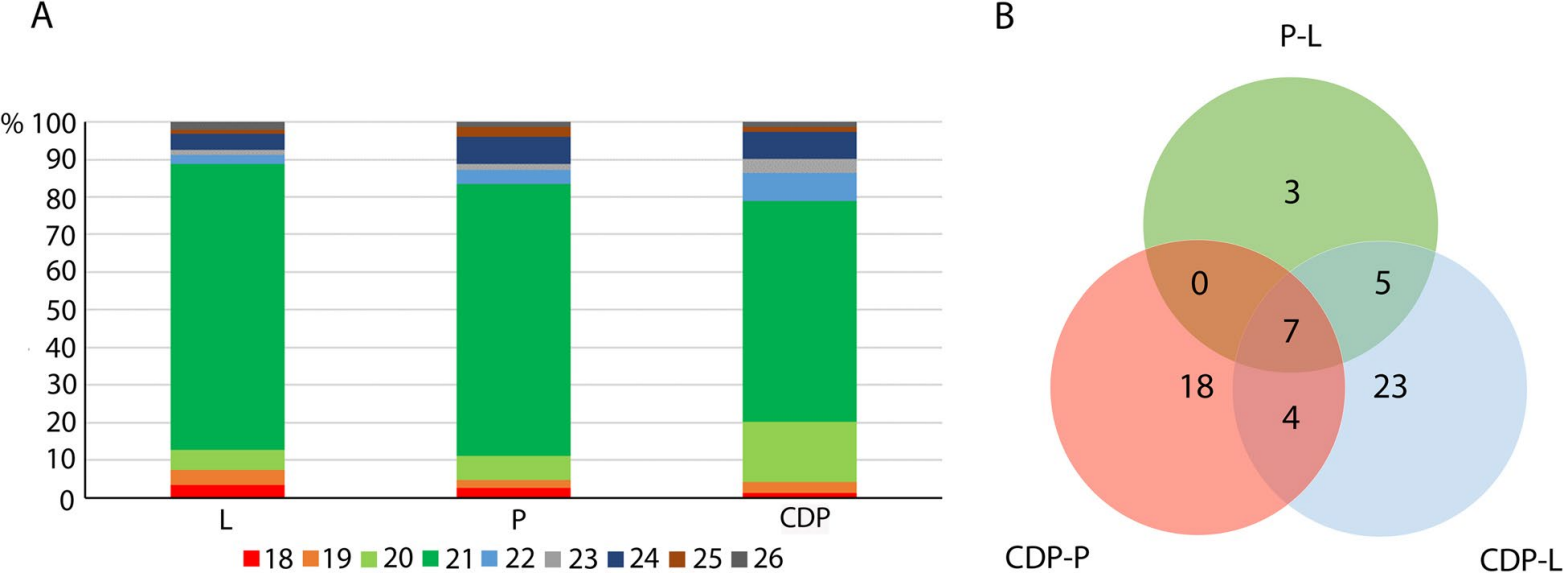
(**A**) Size of the distribution of reads originating from leaves (L), protoplasts (P) and divided cells (CDPs) expressed as a percentage of total reads. (**B**) Venna diagram representing miRNAs differentially expressed in protoplasts vs. leaves (P-L), dividing cells vs. protoplasts (CDP-P) and dividing cells vs. leaves (CDP-L)

To identify miRNAs involved in dedifferentiation, sRNA libraries were prepared from leaves, protoplasts and CDPs after 120 h of culture. High-throughput sequencing led to the generation of 9,251,955 reads from protoplasts and 17,835,610 from leaf cells (Supplementary Data Tab. 1). The majority of the reads were 18 to 26 nt in size, of which the 21-nt class was the most abundant (Fig. 3A). After annotation to the reference genome of *A. thaliana* (v. TAIR10), identification of miRNAs was performed (miRBase database; 21st edition). The number of identified known mature miRNAs in libraries was similar despite different numbers of total read miRNAs (Supplementary Data Tab 2). Next, differentially expressed miRNAs were identified between protoplasts vs. leaves (P-L), dividing cells vs. protoplasts (CDP-P) and dividing cells vs. leaves (CDP-L). The Wald statistical test showed no significant changes in the expression of individual miRNAs between replicates at a given stage (leaves, protoplasts and CDPs after 120 h of culture). During the dedifferentiation of mesophyll cells, 97 miRNAs were differentially expressed, specifically 15, 66 and 76 in P-L, CDP-P and CDP-L, respectively (Fig. 3B). The expression of seven miRNAs was significantly changed in all comparisons, i.e., P-L, CDP-P and CDP-L. Of these miRNAs, miR398b − 3p, miR866-3p, miR399c-5p, and miR398c-3p are involved in abiotic stress regulation, miR319 is involved in cell cycle regulation and leaf development and miR8175 has unknown functions.

In protoplasts, only 15 miRNAs were differentially expressed (DEGs) in comparison to leaves, and they constituted 5% of the identified miRNAs (Table 1). Only 3 of the DEGs were upregulated in comparison to leaves (miR831-5p, miR831-3p, miR866). Hits from the prediction of mRNA targets analyzed during all stages of dedifferentiation for confirmed miRNAs obtained using psRNATarget software are included in the Supplementary Data (Table 3). The function of miR831 has not been fully elucidated; however, recently, it has been shown that its expression is induced during the initial stages of embryogenesis [26] and salt stress [27]. The expression of miR866 is increased upon P starvation [28]. The highest decrease in expression (19-fold) was noted for miR8175. Although the exact function of this miRNA is unknown, an increase in the levels of miR8175 in plants has recently been demonstrated upon exposure to high-intensity light [29] and de-etiolation [30], which is probably associated with the regulation of nuclear-encoded transcripts involved in chloroplast biogenesis and functioning. The reduction in the amount of miR8175 in the protoplasts may be related to their culture in the dark. A significant reduction in expression (17 times) was observed for miR164b-3p. Moreover, the levels of miR398b-3p and miR398c-3p, which target mRNAs encoding CSD1 and CSD2 (Cu-Zn superoxide dismutase), indicating oxidative stress, decreased by approximately 9-fold [31]. The level of miR319, which is involved in plant development and cell division, decreased 13-fold (Table 1).

**Table 1:** Deregulated miRNA in protoplasts vs. leaves. * no reads in leaves, ** no reads in protoplasts; ↑ - increase or ↓ - decrease fold change

***miRNA***	***log***_***2***_***FC***	***miRNA***	***log***_***2***_***FC***
miR831-5p	↑ *	miR397a	-3135
miR866-3p	2946	miR395f	↓ **
miR831-3p	3817	miR395b	↓ **
miR8175	− 4289	miR319b	− 2851
miR408-5p	− 1885	miR319a	− 2341
miR399c-5p	− 2322	miR164b-3p	− 4064
miR398c − 3p	-3147	miR156f-3p	− 2937
miR398b − 3p	-3152		

Comparison between CDP and leaf samples showed that 76 miRNAs were deregulated, accounting for 25.5% of all expressed miRNAs, with half increasing and half decreasing (Table 2). In CDPs, the highest increase, approximately 167 times, was noted for miR866-3p. Predicted targets for miR866 are histone acetyltransferase of the CBP family, nuclear factor Y, subunit A10 and vacuolar sorting receptor 7 [32]. Compared to leaves, the amount of miR319 in CDPs was increased 27 times. This increase correlates with a 6-fold decrease in miR396b-5p, which is negatively regulated by TCP4 – the target of miR319. In addition, miR5028, miR398a-5p and miR4245, which were not expressed in the leaf cells, were observed to be present in CDPs. MiR4245 is intronic in the AGENET DOMAIN-CONTAINING PROTEIN gene (AT5G52070), which undergoes splicing regulation and has recently been shown to be involved in the cold stress response [33]. The largest reduction in miRNA was recorded for miR398b-3p and miR398c-3p, and their expression levels were decreased 308-fold (targets of these miRNAs are CSD1 and CSD2). The miR390 family, which is involved in biogenesis *TAS3* tasiRNA and auxin signaling, also showed significant changes [34]. The amounts of miR390a-5p and miR390b-5p increased threefold, while miR390a-3p increased more than fourfold (Table 2).

**Table 2:** Deregulated miRNA in CDPs cultured for 120 h vs. leaves. * no reads in leaves, ** no reads in CDPS; ↑ - increase or ↓ - decrease fold change

***miRNA***	***log***_***2***_***FC***	***miRNA***	***log***_***2***_***FC***	***miRNA***	***log***_***2***_***FC***	***miRNA***	***log***_***2***_***FC***
miR8175	− 7629	miR858b	− 2684	miR399d	− 2259	miR319a	3504
miR8169	− 3217	miR858a	− 2681	miR399c-5p	− 4568	miR173-3p	1958
miR8168	↓ **	miR841b-5p	3744	miR399c-3p	−2,83	miR172e-5p	2195
miR8166	− 4005	miR841b-3p	3249	miR399b	−5,77	miR172e-3p	2776
miR5996	− 1587	miR841a-5p	1653	miR398c-5p	− 1256	miR171a-3p	− 2496
miR5659	− 3587	miR841a-3p	2,92	miR398c-3p	− 8268	miR168b-3p	3127
miR5653	− 5059	miR840-5p	3907	miR398b-3p	− 8263	miR168a-3p	3259
miR5028	↑ *	miR838	1962	miR398a-5p	↑ *	miR167b	− 1855
miR5026	3038	miR833a-5p	↓ **	miR398a − 3p	-3853	miR167a-5p	− 1854
miR5020b	− 4113	miR831-3p	4044	miR397b	− 1824	miR166b-5p	−1,57
miR5014a-5p	1865	miR824-3p	1897	miR397a	− 4583	miR166a-5p	− 1586
miR2111b-5p	− 1745	miR823	1,97	miR396b-5p	− 2489	miR165a-5p	− 3883
miR2111a-5p	− 1648	miR775	2123	miR396a-3p	3803	miR164b-3p	− 2787
miR2111a − 3p	− 3959	miR773a	1631	miR393a-3p	3449	miR163	-3229
miR866-3p	7386	miR472-3p	2627	miR390b-5p	1527	miR158a-5p	3138
miR866-5p	4446	miR4245	↑ *	miR390a-5p	1603	miR157c-3p	− 2375
miR865-5p	1605	miR408-5p	− 2803	miR390a − 3p	2118	miR156f − 3p	-3863
miR863-3p	2928	miR406	-3,3	miR3434-3p	2697	miR156c-3p	1536
miR859	2261	miR399f	−2,3	miR319b	4041	miR156b − 3p	2502

Next, the expression of miRNA in cells cultured for 120 h (CDPs) was compared with that of protoplasts (Table 3), and 66 deregulated transcript miRNAs were identified (21.5%), of which 39 were upregulated (21.5%) and 27 were downregulated (12%). As many as 71% of miRNAs that are deregulated in this comparison are the same as those observed with CDPs vs. leaves. The highest increase in expression was recorded for miR319b and amounted to approximately 118-fold. MiR396a − 3p was also upregulated in the protoplasts vs. leaves comparison. The largest decrease, 35-fold, was recorded for miR398b-3p, miR398c-3p and miR399b. Molecules such as miR8177, miR5635c and miR8168 were expressed in protoplasts, while their presence was not observed in cultured cells (Table 3).

**Table 3:** Deregulated miRNA in CDPs cultured for 120 h vs protoplast. * no reads in protoplasts. ** no reads in cells cultured for 120 h; ↑ - increase or ↓ - decrease fold change

***miRNA***	***log***_***2***_***FC***	***miRNA***	***log***_***2***_***FC***	***miRNA***	***log***_***2***_***FC***
miR8177	↓ **	miR824-3p	1565	miR390a-3p	2,31
miR8175	-3,34	miR823	1448	miR3434-3p	3,07
miR8169	-3381	miR775	2,53	miR319c	2115
miR8168	↓ **	miR472-5p	2992	miR319b	6892
miR8166	− 4524	miR472-3p	1634	miR319a	4493
miR5996	− 1889	miR406	− 2729	miR173-3p	1285
miR5653	− 4935	miR400	− 1935	miR171b-5p	2076
miR5635c	↓ **	miR399f	−1,78	miR171a − 3p	-3131
miR5028	↑ *	miR399d	− 1881	miR170-3p	−1,94
miR5026	2527	miR399c-5p	− 2247	miR168b − 3p	2526
miR5020b	-3416	miR399c-3p	− 2289	miR168a-3p	2188
miR870-3p	↑ *	miR399b	− 5208	miR167d	− 1919
miR866 − 5p	2741	miR398c-3p	-5122	miR167c-5p	−1587
miR866-3p	4441	miR398b-3p	− 5111	miR167b	− 2199
miR863-3p	1845	miR398a-5p	↑ *	miR167a-5p	− 2194
miR861-5p	2,22	miR396b-5p	−2,68	miR166e-5p	2295
miR859	3,16	miR396a-3p	3763	miR163	− 4361
miR841b-5p	3609	miR395a	2896	miR160c-3p	2201
miR841b-3p	2435	miR393b-3p	2447	miR160a-3p	1559
miR841a-3p	2341	miR393a-3p	4363	miR158a-5p	2528
miR840-5p	4427	miR3933	↑ *	miR157c-3p	− 2204
miR829-5p	4118	miR390a-5p	1504	miR156b-3p	1774

### qPCR analysis of dedifferentiation-responsive miRNAs and their precursor and mRNA

**Fig. 4:**
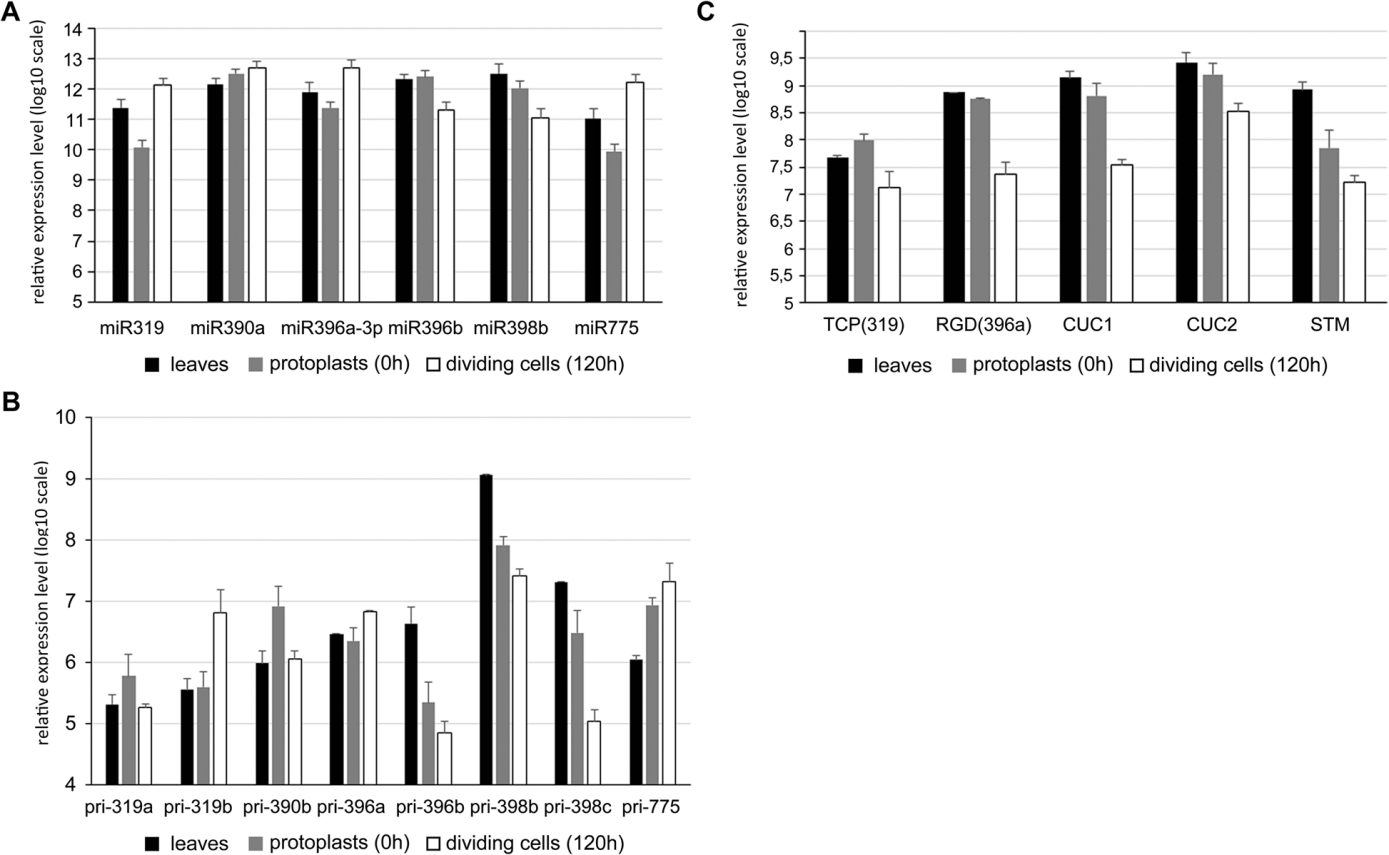
The relative expression of selected miRNAs (**A**), pri-miRNAs (**B**), target mRNAs (TCP-4, RGD) and mRNAs of CUC1/2 and STM (**C**) in leaves, protoplasts (0 h) and dividing cells (CDP 120 h). Vertical bars represent means of biological samples ±SE

To confirm the results obtained by deep sequencing, qPCR for selected miRNAs with changes in expression levels greater than 2.5-fold aside from miR390a (1.6-fold) in response to dedifferentiation was performed. The expression level of miRNAs from high-throughput sequencing and RT-qPCR exhibited exactly the same trends in dividing cells (Fig. 4A). In the protoplasts vs. leaves comparison, the relative expression levels for miR319 and miR398b had similar differences between the two methods (Fig. 4A). qPCR showed slight changes in the levels of 3 miRNAs, despite the absence of changes in NGS results in protoplasts.

We subsequently studied the transcriptional activity of selected *MIR* genes. All analyzed pri-miRNAs were modulated in protoplasts and CDPs (Fig. 4B). In dividing cells, half of the studied *MIR* genes showed an increase, and half exhibited a decrease, in transcriptional activity. There was also a high correlation between the amount of pri-miRNA and miRNA at this stage (Fig. 4A, B). This result may indicate that the transcription intensity of the *MIR* genes positively correlates with processing. The only exception was pri-miR319a, the accumulation of which occurred in protoplasts and a significant decrease in CDP cultured for 120 h. Notably, there was a strong increase in the amount of mature miR319 in dividing cells. Therefore, it appears that pri-miR319b is mainly processed to miR319 during this period (Fig. 4A, B). This result was confirmed by the NGS results, which showed the highest number of miR319b reads in CDPs (Tables 2 and 3). A similar positive correlation was also observed for pri- and miR396a. No such trend was observed in protoplasts. An accumulation of pri-miRNAs (miR775, miR319a, and miR319b) was observed with a decrease in the number of mature miRNAs (Fig. 4A, B). There was also a simultaneous reduction in the amount of pri-miRNA and miRNA (miR398b, miR398c and miR396a) (Fig. 4A, B). Both cases may indicate a reduction or disturbance of pri-miRNA processing in protoplasts. Since the decrease in pri-miR396b is accompanied by an increase in miR396b, the possibility cannot be ruled out that posttranscriptional regulation processes are different for different miRNAs in protoplasts.

MiR319 is one of the most modulated miRNAs after removal of the cell wall and during cell division; therefore, the expression level of its target mRNA was analyzed. The expression of the miR319-dependent TCP4 transcription factor regulating cell division slightly increased in protoplasts but significantly decreased in CDPs (Fig. 4C). This result correlates with a lower level of TCP4-dependent miR396b in CDPs (Fig. 4A). Next, the pathway of miR396a-3p was studied because it is one of the most strongly upregulated miRNAs, although the level of miR396a-5p is stabilized during dedifferentiation. The predicted target for miR396a-3p is RGD3 (ROOT GROWTH DEFECTIVE 3) (psRNATarget). RGD3 is a protein that positively regulates the expression of the CUC1 and CUC2 (CUP-SHAPED COTYLEDON) and STM (SHOOT MERISTEMLESS) genes during the regeneration of shoots from callus tissue. Protoplasting hardly changed the expression of RGD3 and slightly lowered the expression of CUC1, CUC2 and STM. Moreover, in CDPs, the decreased level of RGD3 mRNA was accompanied by a strong reduction in the expression levels of the CUC1/CUC2 and STM genes (Fig. 4C).

### Viability of protoplasts and CDPs derived from wt, *miR319a*^*129*^ and *ΔmiR319b* during dedifferentiation

**Fig. 5:**
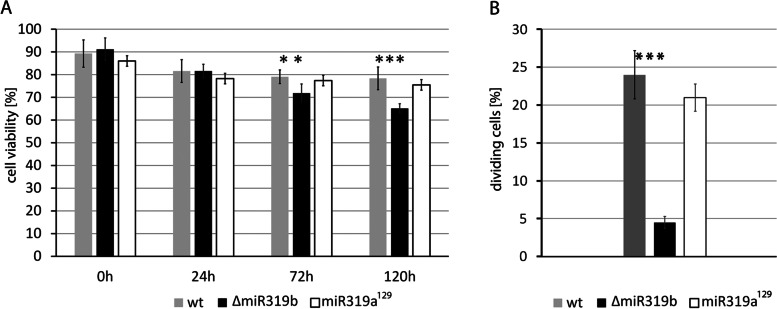
(**A**) Viability of dedifferentiating cells obtained from wt, *ΔmiR319b*, and *miR319a*^*129*^ based on a fluorescein diacetate assay (FDA). (**B**) Proliferation of cells derived from wt, *ΔmiR319b*, and *miR319a*^*129*^ cultured for 120 h. ** and *** indicate statistically significant differences between the groups with test probabilities of *P* < 0.01 and P < 0.001, respectively, Student’s t-test, α = 0.05. Error bars represent standard deviations

Subsequently, we tested the viability of the protoplasts and the ability of *miR319a*^*129*^ and *ΔmiR319b* to dedifferentiate cells. The percentages of viable wt and mutant cells in the subsequent stages of dedifferentiation are shown in Fig. 5A. There were no significant differences in survival between wt and mutant protoplasts and cells cultured for 24 h. The differences between the studied variants of cells were observed 72 h and 120 h after the removal of the cell wall. In CDP cultured for 120 h 79.36 and 64.94% of viable cells were observed in wt and *ΔmiR319b*, respectively. However, no significant differences were observed between wt and *miR319a*^*129*^ (Fig. 5A). In CDPs derived from *ΔmiR319b*, the ability to proliferate was low, as we observed ~ 24% dividing cells in wt cells after 120 h of culture and ~ 21% dividing cells in *miR319a*^*129*^ cells but only ~ 4.5% dividing cells in *ΔmiR319b* cells (Fig. 5B).

## Discussion

The results obtained with *A. thaliana dcl1–9* and *ΔmiR319b* mutants indicate that miRNA-mediated regulation of gene expression is an important mechanism for plant cell dedifferentiation stimulated by cell wall removal. There are several reports concerning the role of posttranscriptional gene regulation via miRNAs in plant regeneration. However, most of these reports concern the miRNA function in the regulation of the differentiation of calli, somatic embryogenesis or protoplasts isolated from calli [35–38]. However, our research indicates the participation of miRNAs in dedifferentiation mesophyll cells (completely differentiated cells) and enables the analysis of individual cells undergoing this process.

The results obtained in this study indicate that the miRNA system is not equally important in all stages of dedifferentiation from protoplast isolation to first cell division. Removal of the cell wall strongly reduced the cytoplasmic accumulation of AGO1, which is a permanent component of RISC and DBs recognized as miRNA biogenesis centers [39]. These data suggest a low level of miRNA biogenesis and miRNA activity in the regulation of gene expression in protoplasts and CDPs cultured for 24 h, a period spanning the first stages following cell wall removal. The above observations confirm the analysis of the miRNA transcriptome. The only 15 miRNAs in the protoplasts had a different level of expression compared to the leaf cells. Protoplasts are examples of the nonphysiological state of a plant cell. Stress symptoms in these cells have been demonstrated at the molecular and structural levels; these symptoms include decreased chromatin condensation [5, 6, 40–42]. It has been shown that in protoplasts, substantial changes in the transcriptome include genes involved in the response to stress [5]. miRNAs are considered to be specific markers of various types of plant stresses [27, 43]. However, in this study, it was shown that only a small number of miRNAs were deregulated in protoplasts compared with leaves. It follows that in the first stage of dedifferentiation caused by removal of the cell wall, the miRNA pathway plays a minor role. This phenomenon may be observed because the response mechanism of protoplast isolation is completely different from that to abiotic stresses because plant cells do not experience this phenomenon under natural conditions. Nevertheless, the results of this study on miRNA deregulation in protoplasts indicate the presence of oxidative stress, inhibition of the cell cycle and enhanced availability of several nutrients. In our study, the amount of miR395 was lowered in protoplasts, which indicates better sulfur availability. The miR395 gene family (miR395a, d, e, and miR395b, c, f) suppresses mRNA encoding the low-affinity sulfate transporter SULTR2;1 and the ATP sulfurylases APS1, APS3 and APS4. It has been shown that under sulfur deficiency, the expression levels of miR395 family genes increase significantly [44, 45]. Additionally, we showed deregulation of miR399c-3p and miR397a, which indicates similar mechanisms with better availability of phosphorus and copper, respectively [45–47]. Since similar trends in the expression of the above miRNAs were observed during cell division in CDPs (except for miR395), it can be presumed that the improved availability of these ions is due to single-cell suspension culture and not the lack of a cell wall in protoplasts.

The analysis of the amount of DBs suggests that miRNA biogenesis returns to the state observed before isolation after 72 h of culture, while the increase in the amount of AGO1 in the cytoplasm, which is an element of RISC, indicates that miRNA activity increases during early cell division. These increases are confirmed by the results of miRNA sequencing after 120 h of culture. The percentage of divided cells was 35% and microcalls was 3% in CPD cultured for 120 h. This makes it possible to consider that changes in the micro-transcriptome are the result of dedifferentiation and first cell division. In our work, we analyze miRNAs with a significant difference in quantity between the studied stages. After the decrease in miR319b expression in protoplasts, there was a strong increase in these miRNAs in CDP cells cultured for 120 h. The miR319 family posttranscriptionally regulates the mRNA expression of TCP transcription factors (TEOSINTE BRANCHED/CYCLOIDEA/PROLIFERATING CELL FACTORS) [48]. Of the 24 TCP genes known in *Arabidopsis*, five belong to TCP class II factors (TCP2, TCP3, TCP4, TCP10, and TCP24), which are considered to be negative growth regulators. TCP4 regulates the expression of miR396 by binding cis elements in the promoter region [49]. GRF (GROWTH REGULATING FACTOR) activity is posttranscriptionally repressed by miR396. GRF interacts with GIFs (GRF-INTERACTING FACTOR) and functions in promoting the proliferation of *Arabidopsis* cells during leaf development. In the double mutant *grf* and *gif*, a synergistic reduction in leaf size and cell number was observed, and overexpression of both stimulated leaf growth [50].This pathway was disclosed by our results in dedifferentiation. The decrease in TCP4 expression correlates with a decrease in the level of pri-miR396b and, consequently, of mature miR396b. It follows that miR319 is significantly involved in the dedifferentiation of mesophyll cells by initiating the first divisions.

Notably, TCP4 regulated by miR319 activates VND7 transcription by directly interacting with its promoter. VND7 is involved in secondary cell wall biosynthesis and programmed cell death in the stem, especially in xylem vessel formation [51]. The participation of miRNA in the composition of the cell wall is also indicated by changes in the expression of miR775 in the present study of dedifferentiation. In *Arabidopsis thaliana* with decreased expression of miR775, whose target gene is GALT9 (galactosyltransferase 9), an increase in pectin accumulation has been shown [52]. Pectins might enhance crosslinking with cellulose, which, in turn, increases elastic resistance to internal turgor pressure [53]. Our results indicated that the decrease in the amount of TCP4 as a result of miR319 activity as well as the increase in the amount of miR775 in CDPs reduced the amount of pectins, which may cause stiffening of the cell wall. This mechanism enables intracellular pressure to be established and promotes cell division. However, the participation of miR319 and miR775 in remodeling the cell wall during dedifferentiation warrants further study.

**Fig. 6:**
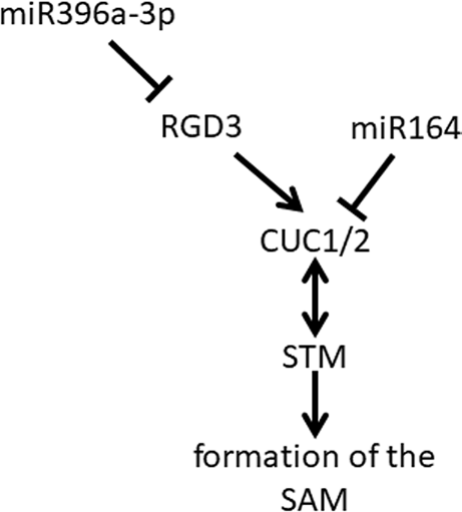
Scheme of the role of miR396a-3p in inhibiting de novo apical meristem formation during dedifferentiation

A significant increase in the level of expression was noted for miR396a-3p in CDPs cultured for 120 h vs. leaves and vs. protoplasts. On the other hand, the amount of miR396a-5p formed from the same pri-miRNA was stable in the studied stage (NGS data). Moreover, an increase in miR396a-3p was demonstrated during salt stress and copper deficiency [27]; however, the function of this miRNA needs to be elucidated. The putative target of miR396a-3p is RGD3 (ROOT GROWTH DEFECTIVE3) (psRNATarget, [54]), a protein that positively regulates the expression of the CUC1 and CUC2 (CUP-SHAPED) genes during the regeneration of shoots from calli [55]. CUC (CUP-SHAPED COTYLEDON) and STM (SHOOT MERISTEMLESS) contribute to the formation of the SAM (shoot apical meristem) [56]. CUC2 is also a central component of the regulatory pathway that controls the outline of the leaf margin [56]. In studies on the dedifferentiation of mesophyll cells, we showed a positive correlation between the amount of pri-miR396a and mature miR396a-3p in protoplasts and CDPs cultured for 120 h. This increase in dividing cells is accompanied by a decrease in the level of the putative RGD3 target mRNA, which is correlated with a slight reduction in the expression of CUC1 and CUC2 in protoplasts and is strong in dividing cells. A decrease in STM mRNA was observed as well. In *Arabidopsis*, CUC genes are required for the activation of STM during embryogenesis and it has been proposed that STM can in turn activate CUC expression [57]. This effect contributes to the inhibition of differentiation, leading to the formation of SAM-type cells during the dedifferentiation of mesophyll cells. The expression of CUC1 and CUC2 is subject to posttranscriptional regulation by the miR164 family [55]. However, significant changes in the amount of miR164a/b/c/− 5p were not observed in protoplasts or in CDPs cultured for 120 h (NGS data). These results indicate that regulation of miR396a-3p can inhibit de novo apical meristem formation during dedifferentiation without significantly reducing proliferation, keeping protoplasts and CPDs in a totipotent state (Fig. 6). The results obtained in this study also show that unlike in leaves, CUC genes can be regulated by RGD3, not by miR164, and their expression is not necessary for the dedifferentiation of mesophyll cells.

## Conclusion

The regulation of RNA metabolism is involved in the in vitro dedifferentiation of plants. In this study, we investigated the function of miRNAs in the dedifferentiation of *A. thaliana* mesophyll cells in a process stimulated by the enzymatic removal of the cell wall. miRNAs participate in organogenesis, oxidative stress, nutrient deficiencies and cell cycle regulation in protoplasts and CDPs. Expression of the *MIR319*, *MIR396* and *MIR398* genes seems to be crucial in the process of dedifferentiation and their appropriate genetic manipulation may increase the efficiency of plant regeneration from protoplasts and can be employed for the rapid, commercial-scale propagation.

## Methods

### Protoplasts isolation and in vitro culture

Protoplasts were isolated from wt, *dcl1–9* [11], *miR319a*^*129*^ [58] and *ΔmiR319b* (SALK_037093) *A. thaliana* Col-0 21-day-old plantlets grown in in vitro culture with 16/8 h day/night photoperiodlight with 150 μmol m^− 2^s^– 1^ irradiance and ambient temperature of 22 °C. Approximately 0,6 g of chopped leaves was added to maceration medium. The isolation and culturing of protoplasts and all necessary solutions were prepared according to the protocol of Chupeau et al. [5]. Protoplasts were isolated by incubating approximately 1.2–1.5 g of cut leaves into 10 ml of the enzyme mixture (Supplementary Data Tab. 4). Isolation was carried out 12–14 h at 24 °C in the dark on a shaker with a rotation speed of 70 rpm (d = 10 mm). Then the obtained cell suspension was filtered through a 60 μm mesh filter into 50 ml round-bottomed test tubes, 8 ml of washing solution (2.5% KCl, 0.2% CaCl2) was added and centrifuged 7–8 min, 60 g. Protoplast pellets were suspended in 10 ml of washing solution and centrifuged as before, washing was repeated three times. Approximately 3.5–5.5 × 10^7^ viable protoplasts were routinely isolated from plantlets. Then protoplast suspension was diluted in initial protoplast induction medium (PIM) (Supplementary Data Tab. 5) to a concentration of 8 × 10^4^ protoplasts per milliliter. The protoplasts were grown in the dark at 20 °C, on a shaker with a rotation speed of 70 rpm (d = 10 mm). Leaves, protoplasts and cells derived cultured for 24, 72 and 120 h were fixed in 4% formaldehyde in 50 mM PIPES pH 7.2, 16–18 h at 4 °C. The viability and division rate of wt, *dcl1–9*, *miR319a*^*129*^ and *ΔmiR319b* cells were determined based on a fluorescein diacetate assay (FDA) (Merk) on a Malassez slide. To 300 μl of the cell suspension was added 0.2 μl of a fluorescein diacetate solution (5 mg/ml acetone). The results were examined with an Olympus BX 50 microscope and documented with an Olympus XC50 camera, using a 100 x (numerical aperture: 1.4) immersion oil objective. At least 500 cells were analyses for each stage. Statistical analysis was performed using PAST. To compare two groups, Student’s t-tests were used.

### Localization of AGO1 and U2snRNA

Localization of AGO1 was performed on protoplasts, cells cultured and isolated nuclei [59]. For permeabilization of the cell membrane, we used increasing dilutions of Triton X-100 in PBS from 1:5000 for protoplasts to 1:2000 for CDP cultured for 5 days (120 h). To localize the AGO1, the cells were incubated with a primary anti-AGO1 rabbit polyclonal antibody (Agrisera) diluted 1:200 in 0.01% acBSA in PBS at 4 °C overnight. The secondary antibody was anti-rabbit antibody labeled with Alexa 488 (Molecular Probes, NY, USA) diluted in 0.01% acBSA in 1x PBS at a ratio of 1:500, and incubation was performed at 35 °C for 1 h. In situ hybridization to U2 snRNA was conducted on the HYL1-YFP *A. thaliana* [16] line. Hybridization was performed at 30 °C for 16–18 h using antisense U2 snRNA 5′ Cy3-ATATTAAACTGATAAGAACAGATACTACACTTG (1:500 diluted) (Genomed, Poland). Control reactions were conducted without primary antibodies or oligo probes. All procedures were conducted in 1.5-ml Eppendorf tubes, and when solutions were changed, the samples were centrifuged for 3.5 min (0.1×g for protoplasts and CDP, 0.3×g for isolated nuclei). The FISH and immunolocalization results were analyzed with a Nikon PCM-2000 confocal microscope and fluorescence inverted Nikon Eclipse TE 2000-E microscope. A 100× (numerical aperture, 1.3) Plan Fluor DIC H/N2 oil immersion lens was used. The images were collected under consistent acquisition conditions (laser power, emission band, gain and resolution) to ensure comparable results. For each material (mesophyll-isolated nuclei and cells, protoplasts and CDP), we obtained three-dimensional optical sections with a 0.5-μm step interval from 42 to 56 cells, depending on the stage. For signal evaluation ImageJ software were used. The signal intensity per cell/nucleus was measured and expressed in relative fluorescence units.

The analysis of the number and location of D-bodies was performed on the basis of 3 independent isolations and protoplast cultures from HYL1-YFP transgenic plants. At least 100 cells were analyzed in each replicate and in each stage. Statistical analysis of the quantity of AGO1 and D-bodies was performed using the PAST program and Microsoft Excel (Microsoft, Redmond, WA, USA). To compare all groups and to determine if there were any significant differences between them, a nonparametric Kruskal-Wallis test was used. To test between which group differences existed, a Mann-Whitney U test with Bonferroni correction was used.

### RNA isolation

Total RNA was isolated from 100 mg 21-day-old leaves, protoplasts and cultured cells of *A. thaliana* in four replicates each, and two replicates of the best RNA quality from each experiment were used for preparation of NGS libraries. Total RNA was isolated with Trizol reagent supplemented with 0.5% (w/v) N-lauroylsarcosine sodium salt, 3 mM β-mercaptoethanol and 5 mM EDTA. After phase separation, one phenol/chloroform and two chloroform extractions were performed. The aqueous phase (500 μL) was mixed with 3 μL of GlycoBlue (Invitrogen; 15 mg /μL) before RNA was precipitated with 625 μL of ethanol and 250 μL of 0.8 M sodium citrate/1.2 M sodium chloride. Samples were incubated for 30 min. at room temperature and then centrifuged (25 min., 16,000 g, 4 °C). The precipitate was washed with 80% (v/v) ethanol, air dried, and dissolved in 2 mM Tris-HCl (pH 7.5). RNA concentration was measured with a NanoDrop ND-1000 (NanoDrop Technologies), and integrity was measured with an Agilent-2100 Bioanalyzer (Agilent Technologies; RNA 6000 NanoChip).

### Preparation of small RNA libraries and deep sequencing

Total RNA of the best quality from two replicates of each experiment were used for preparation of cDNA libraries for NGS. Total RNA from each sample (5 μg) was mixed with 2x loading buffer II (Ambion), denatured for 2 min at 90 °C, and separated on a 15% polyacrylamide/7 M urea/1x TBE gel at 300 V. 10-bp DNA ladder (Invitrogen) were used to localize small RNAs (18–30 nucleotides) as well as ligation and PCR products on gels stained with SYBR Gold (Invitrogen). RNA and PCR products were eluted from polyacrylamide gels in 300 μL of EBR buffer (50 mM Mg acetate, 0.5 M ammonium acetate, 1 mM EDTA, and 0.1% SDS) for 10 to 16 h at 20 °C (300 rpm). After phenol/chloroform and chloroform extraction, the aqueous phase was mixed with 1,5 μL of glycogen and 900 μL of 96% (v/v) ethanol, then cooled to -20 °C for 2 h and centrifuged (25 min, 16,000 g, 4 °C). The RNA pellet was washed twice with 80% (v/v) ethanol and dissolved in 6 μL of water. 5′ and 3′ RNA adaptor ligations with RNA primers, RT, and PCR were performed according to Lu et al. (2007) [60], except for a 3′ RNA adaptor 3′ end modification consisting of a C3 hydrocarbon spacer (Biomers.net). Reverse transcription was performed with SuperScript™ II Reverse Transcriptase (Invitrogen), at 65 °C, and RT was inactivated at 95 °C for 5 min. The PCR (25 μL) with Illumina indexing primers was terminated with 75 μL of stop buffer (10 mM Tris-HCl, pH 8.0,1 mM EDTA, and 0.4 M ammonium acetate). After phenol (pH = 8.0)/ chloroform extraction, 2 μL of GlycoBlue (Invitrogen; 15 μg /μL) and 300 μL of ethanol were added to precipitate cDNA. The cDNA was denatured in loading buffer II (Ambion) and separated on an 8% polyacrylamide/7 M urea gel. The cDNA band was eluted and precipitated as above. The pellet was washed with 70% (v/v) ethanol, air dried, and dissolved in 14 μL of water. The cDNA concentration was measured using a NanoDrop ND-1000 and checked by 15% polyacrylamide/7 M urea gel electrophoresis with oligonucleotides of known concentration. Illumina sequencing and bioinformatic analysis was performed at FASTERIS SA (Switzerland).

### Quantitative real-time PCR profiling of pri-miRNAs, miRNAs, mRNA

All qPCR were performed as previously described [61] using a 7900HT Fast Real-Time PCR System (Applied Biosystems by Life Technologies). Briefly, total RNA for cDNA preparation was isolated as for preparation of small RNA libraries. For pri-miRNA and mRNA targets expression analysis cDNA synthesis was prepared with the use of SuperScript III Reverse Transcriptase (ThermoFisher Scientific), oligo-dT(18) primer (ThermoFisher Scientific) and 3 μg of total DNase I treated RNA (ThermoFisher Scientific). qPCRs were performed with Power SYBRR Green PCR Master Mix (ThermoFisher Scientific). The mRNA fragments of glyceraldehyde-3-phosphate dehydrogenase (GAPDH, At1g13440) gene were used as reference. Primers sequences for pri-miRNAs were retrieved from miREX database [62]. Primers used for mRNA are listed in Tab. 6 Supplementary Data. For mature miRNA analyses, RT reactions were prepared using MultiScribe Reverse Transcriptase (ThermoFisher Scientific), 10 ng of total DNase-treated RNA and 5 × RT primer specific for each miRNA or U6 snRNA used as a reference (ThermoFisher Scientific). qPCRs were performed with TaqMan Universal Master Mix II with UNG (ThermoFisher Scientific), TaqMan probes and primers specific for mature miRNAs and internal reference gene (U6 snRNA, At3g14735). All qPCR results were analyzed using SDS 2.4 software (ThermoFisher Scientific). Error bars were calculated using the SD Function in Microsoft Excel software. The fold change was calculated using the 2^-ΔΔCt method.

## Supplementary Information

**Additional file 1.**

## Data Availability

The data and material supporting the findings of this study are available from the corresponding author (K.D., J.N.) upon request.

## Abbreviations

AGO1: Argonaute1
CB: Cajal body
CDPs: cells derived from protoplasts
CSD: Copper superoxide dismutase
CUC1: Cup shaped cotyledone
DBs: dicing bodies
DCL1: Dicer-like 1
GRF: Growth regulating factor
HYL1: Hyponastic leaves 1
RGD3: Root growth defective
STM: Shoot meristemless
TCP4: Teosinte branched/cycloidea/proliferating cell factors
