# Supplementary material for: MicroRNA biogenesis and activity in plant cell dedifferentiation stimulated by cell wall removal
Source: BMC Plant Biol. 2022 Jan 3;22:9. doi: 10.1186/s12870-021-03323-9 (PMC8722089; doi:10.1186/s12870-021-03323-9)
Supplement: Supplementary file 1 — Additional file 1. [file 12870_2021_3323_MOESM1_ESM.docx]

Supplementary Data

[%]

Fig. 1 Percentage of divided, undivided and microcalli cells in CDPs cultured for 72h and 120h

wt *dcl1-9*


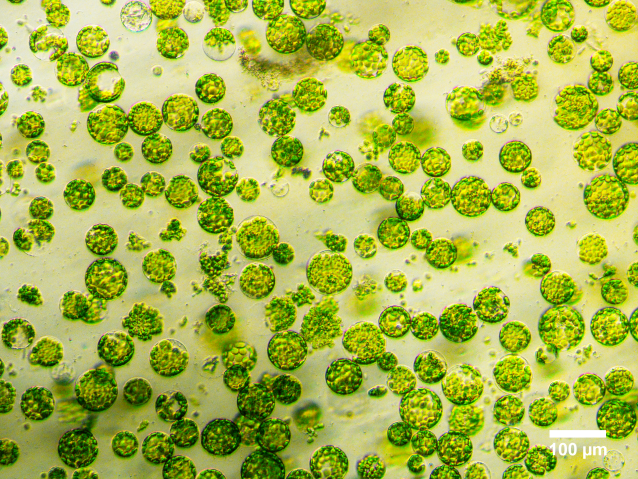

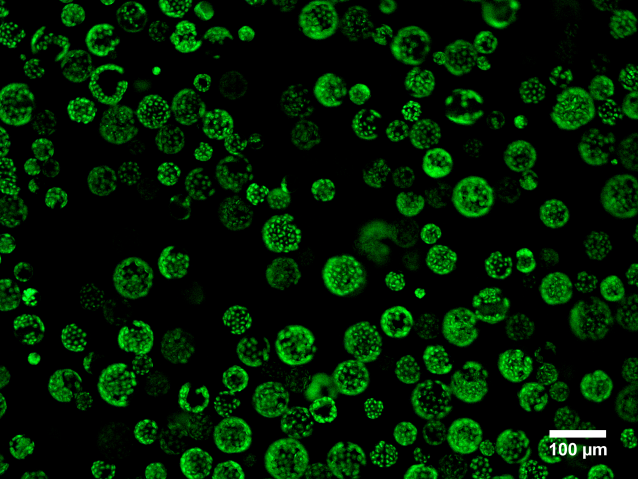

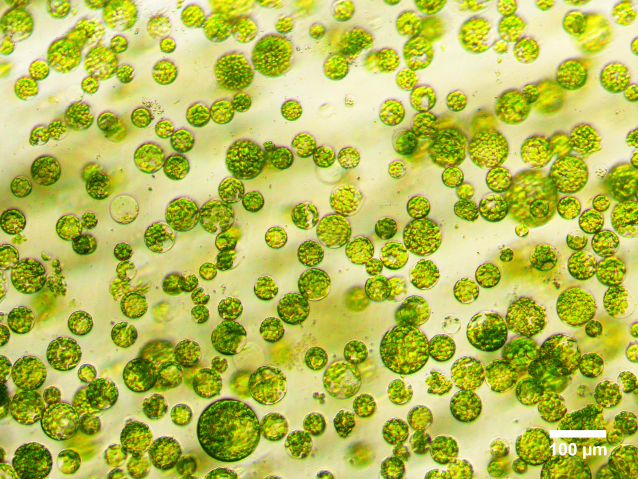

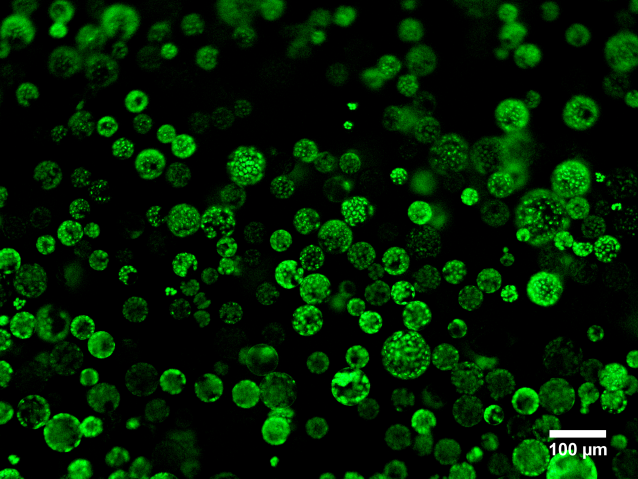

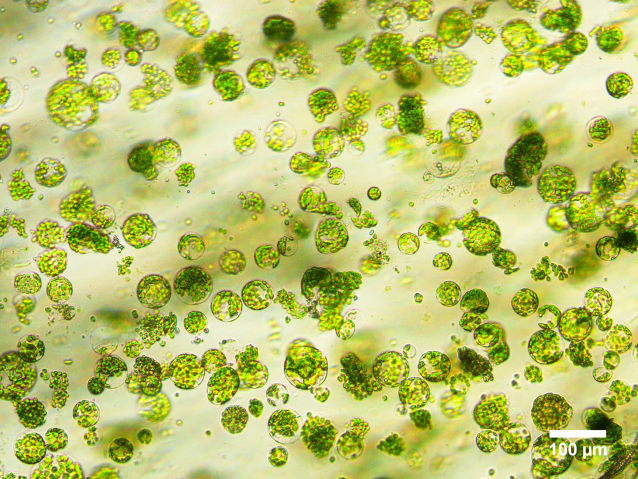

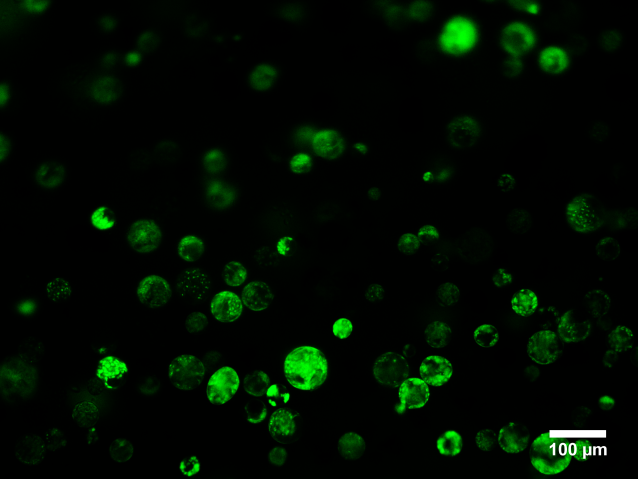

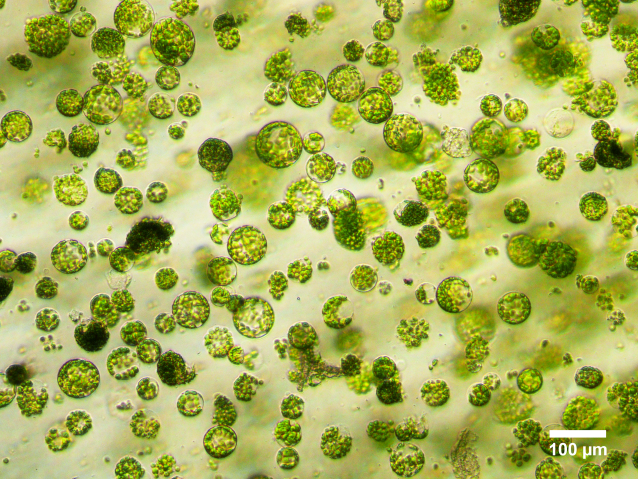

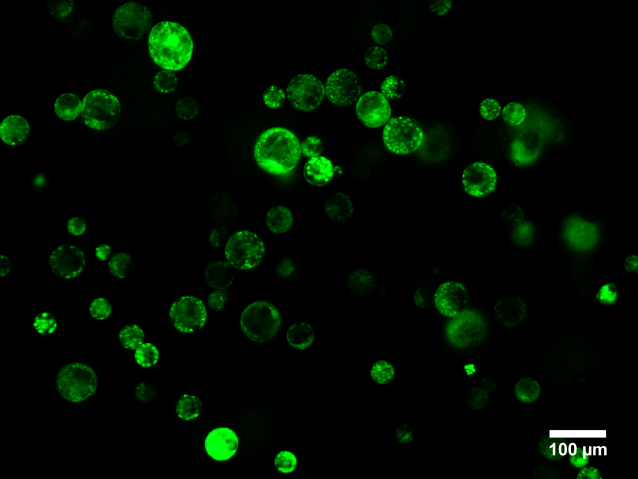


**CDP 24h**

**protoplasts 72h**

**B**

**A**

**D**

**C**

**F**

**E**

wt *dcl1-9*


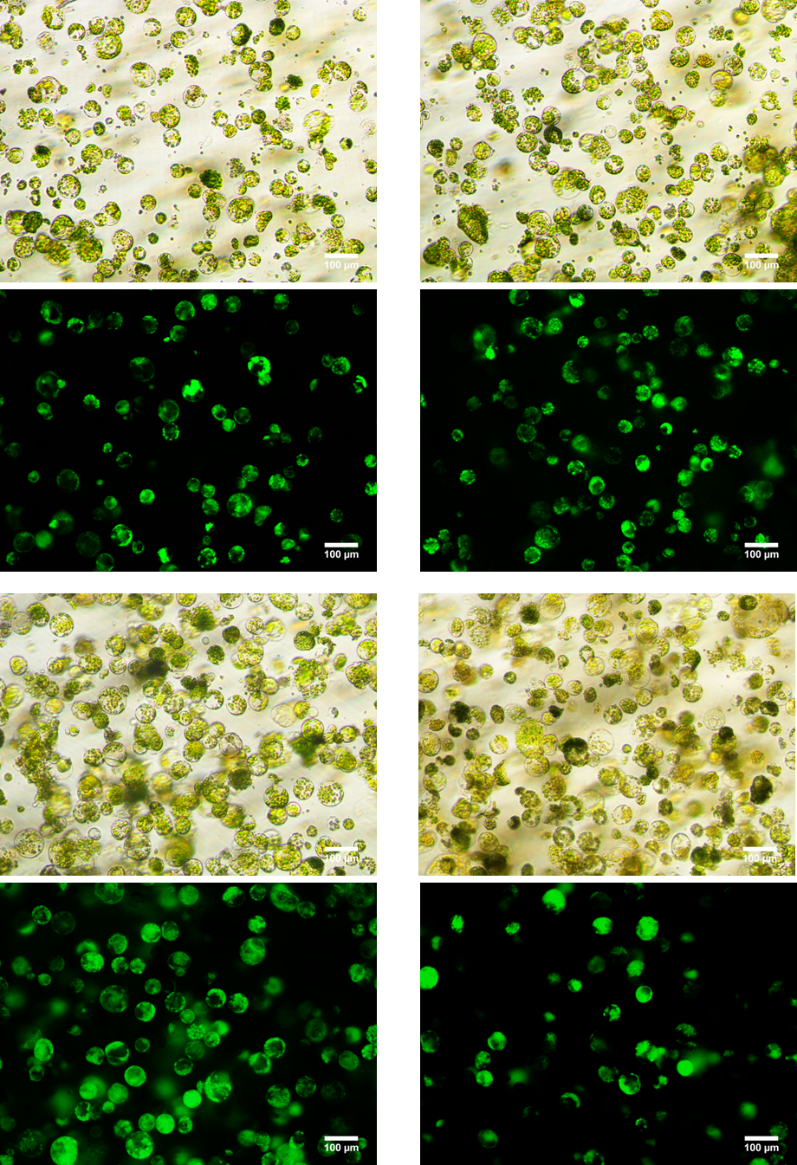


Fig. 2 Viability of protoplasts and CDP cultured for 24, 72, 120h derived from wt (A, C, E, G, I, K, M, O) and dcl-1 (B, D, F, H, J, L, N, P), based on the comparison of morphology and the number of cells observed in the transmission channel and fluorescence microscopy (fluorescein diacetate assay); Bar 100 µm

**P**

**O**

**N**

**M**

**L**

**K**

**J**

**I**

**CDP 120h**

**CDP 72h**

**P**

Fig. 3 Percentage of cells containing 1 to 8 DBs during dedifferentiation

Tab. 1 Sequenced inserts divided into classes according to the length expressed in nucleotides [nt] and their percentage share in each of the libraries. 0- sequences without inserts, * sequences in which the insert was not detected (adapter was outside the sequence reading length or an error in its sequence was found that made it impossible to match); 1L, 2L –leaves, 1P, 2P –protoplasts, 1CDP 120h, 2 CDP 120h – dividing cells

| Insert’s length  [nt]  Libraries |  | 0 | 1-17 | 18-26 | 27-44 | Others* |
| --- | --- | --- | --- | --- | --- | --- |
| 1L | l. reads | 106 997 | 4 288 543 | 13 167 612 | 37 066 | 235 392 |
|  | % reads | 0,60 | 24,04 | 73,83 | 0,21 | 1,32 |
|  | % inserts | 0,61 | 24,37 | 74,82 | 0,21 | Nd |
| 2L | l. reads | 562 864 | 2 183 985 | 10 053 061 | 198 607 | 250 892 |
|  | % reads | 4,25 | 16,48 | 75,88 | 1,50 | 1,89 |
|  | % inserts | 4,33 | 16,80 | 77,34 | 1,53 | Nd |
| 1P | l. reads | 144 781 | 2 956 431 | 8 177 525 | 226 709 | 217 423 |
|  | % reads | 1,24 | 25,22 | 69,76 | 1,93 | 1,85 |
|  | % inserts | 1,26 | 25,70 | 71,08 | 1,97 | Nd |
| 2P | l. reads | 27 628 | 2 043 231 | 6 820 831 | 191 671 | 168 594 |
|  | % reads | 0,30 | 22,08 | 73,72 | 2,07 | 1,82 |
|  | % inserts | 0,30 | 22,49 | 75,09 | 2,11 | Nd |
| 1CDP 120h | l. reads | 770 777 | 7 687 435 | 6 545 109 | 12 640 | 260 667 |
|  | % reads | 5,05 | 50,32 | 42,84 | 0,08 | 1,71 |
|  | % inserts | 5,13 | 51,20 | 43,59 | 0,08 | Nd |
| 2CDP 120h | l. reads | 614 379 | 6 235 225 | 7 115 503 | 12 065 | 228 620 |
|  | % reads | 4,32 | 43,89 | 50,09 | 0,08 | 1,61 |
|  | % inserts | 4,40 | 44,61 | 50,91 | 0,09 | Nd |

Tab. 2 Number of known mature miRNAs in individual libraries

| **Libraries** | **Number of miRNAs** | **Total reads miRNA** |
| --- | --- | --- |
| 1L | 287 | 9 296 136 |
| 2L | 290 | 6 449 774 |
| 1P | 263 | 6 486 518 |
| 2P | 299 | 6 486 518 |
| 1CDP 120h | 303 | 3 845 713 |
| 2CDP 120h | 308 | 3 816 493 |

Tab. 3 Hits from the prediction of mRNA targets for confirmed miRNAs, obtained using the psRNATarget software. A higher expectation value (Exp.) indicates less similarity between small RNA and the target candidate (lower probability of true interaction). The target gene regulation can be realized by its cleavage or translation inhibition.

| **miRNA** | **samples** | **Exp** | **Inhibition** | **Target Acc.** | **Target name** | **Target function** |
| --- | --- | --- | --- | --- | --- | --- |
| miR156b-3p | CDP-P  CDP-L | 3,0 | Cleavage | AT2G36690.1 |  | 2-oxoglutarate (2OG) and Fe(II)-dependent oxygenase superfamily protein |
|  |  | 3,0  3,5 | Cleavage  Translation | AT5G35792.1  AT3G29175.1 |  | Transposable element gene |
|  |  | 3,0 | Cleavage | AT1G09380.1 |  | Nodulin MtN21 /EamA-like transporter family protein |
|  |  | 3,5 | Cleavage | AT2G20880.1 |  | Integrase-type DNA-binding superfamily protein |
|  |  | 3,5 | Cleavage | AT3G12670.1 | emb2742 | CTP synthase family protein |
|  |  | 3,5 | Cleavage | AT4G33440.1 |  | Pectin lyase-like superfamily protein |
|  |  | 3,5 | Translation | AT3G47950.1 | AHA4, HA4 | H(+)-ATPase 4 |
|  |  | 3,5 | Cleavage | AT1G03370.1 |  | C2 calcium/lipid-binding and GRAM domain containing protein |
|  |  | 3,5 | Cleavage | AT1G03080.1 |  | Kinase interacting (KIP1-like) family protein |
|  |  | 3,5  3,5  3,5 | Translation  Translation  Translation | AT3G16800.1  AT3G16800.1  AT3G16800.1 |  | Protein phosphatase 2C family protein |
| miR156c-3p | CDP-L | 2,0  2,0 | Cleavage  Cleavage | AT5G25220.1  AT5G25220.2 | KNAT3 | KNOTTED1-like homeobox gene 3 |
|  |  | 2,5  3,5 | Translation  Translation | AT2G39410.2  AT2G39420.1 |  | Alpha/beta-Hydrolases superfamily protein |
|  |  | 2,5 | Cleavage | AT4G19640.1 | ARA7, ARA-7, ATRABF2B, ATRAB5B, RABF2B, ATRAB-F2B, RAB-F2B | Ras-related small GTP-binding family protein |
|  |  | 3,0 | Cleavage | AT1G12775.1 |  | Pentatricopeptide repeat (PPR) superfamily protein |
|  |  | 3,0 | Translation | AT1G18160.1 |  | Protein kinase superfamily protein |
|  |  | 3,5  3,5 | Cleavage  Cleavage | AT2G43180.1  AT2G43180.1 |  | Phosphoenolpyruvate carboxylase family protein |
|  |  | 3,5 | Cleavage | AT3G56330.1 |  | N2,N2-dimethylguanosine tRNA methyltransferase |
|  |  | 4,0 | Cleavage | AT1G50460.1 | HKL1, ATHKL1 | Hexokinase-like 1 |
|  |  | 4,0  4,0 | Cleavage  Cleavage | AT3G44590.1  AT3G44590.2 |  | 60S acidic ribosomal protein family |
|  |  | 4,0 | Cleavage | AT1G75230.2 |  | DNA glycosylase superfamily protein |
| miR156f-3p | P-L  CDP-L | 3,5 | Cleavage | AT3G45130.1 | LAS1 | Lanosterol synthase 1 |
|  |  | 3,5 | Cleavage | AT1G22340.1 | AtUGT85A7, UGT85A7 | UDP-glucosyl transferase 85A7 |
|  |  | 3,5 | Cleavage | AT5G20280.1 | ATSPS1F, SPS1F | Sucrose phosphate synthase 1F |
|  |  | 3,5 | Cleavage | AT4G31590.1 | ATCSLC05, CSLC05, ATCSLC5, CSLC5 | Cellulose-synthase-like C5 |
|  |  | 3,5 | Translation | AT3G54040.1 |  | PAR1 protein |
|  |  | 3,5 | Cleavage | AT1G80350.1 | ERH3, AAA1, FRA2, LUE1, ATKTN1, KTN1, FRC2, BOT1, FTR | P-loop containing nucleoside triphosphate hydrolases superfamily protein |
|  |  | 3,5 | Cleavage | AT4G22285.1 |  | Ubiquitin C-terminal hydrolases superfamily protein |
|  |  | 4,0 | Cleavage | AT2G23350.1 | PAB4, PABP4 | Poly(A) binding protein 4 |
|  |  | 4,0  4,0 | Cleavage  Cleavage | AT1G61010.2  AT1G61010.3 | CPSF73-I | Cleavage and polyadenylation specificity factor 73-I |
|  |  | 4,0 | Translation | AT3G57350.1 |  | Nucleoporin interacting component (Nup93/Nic96-like) family protein |
| miR157c-3p | CDP-P  CDP-L | 3,0 | Cleavage | AT1G45120.1 |  | Transposable element gene |
|  |  | 3,0 | Cleavage | AT5G08650.1 |  | Small GTP-binding protein |
|  |  | 3,0 | Cleavage | AT1G11400.3 | PYM | Partner of Y14-MAGO |
|  |  | 3,0  3,0 | Cleavage  Cleavage | AT5G04560.1  AT5G04560.2 | DME | HhH-GPD base excision DNA repair family protein |
|  |  | 3,5  3,5 | Cleavage  Cleavage | AT1G01620.1  AT1G01620.2 | PIP1C, TMP-B, PIP1;3 | Plasma membrane intrinsic protein 1C |
|  |  | 3,5 | Cleavage | AT2G06990.1 | HEN2 | RNA helicase, ATP-dependent, SK12/DOB1 protein |
|  |  | 3,5 | Cleavage | AT1G75640.1 |  | Leucine-rich receptor-like protein kinase family protein |
|  |  | 3,5 | Cleavage | AT1G17630.1 |  | Pentatricopeptide repeat (PPR-like) superfamily protein |
|  |  | 3,5 | Cleavage | AT5G62670.1 | AHA11, HA11 | H(+)-ATPase 11 |
|  |  | 4,0 | Cleavage | AT2G06990.1 | HEN2 | RNA helicase, ATP-dependent, SK12/DOB1 protein |
| miR158a-5p | CDP-P  CDP-L | 2,0  2,0 | Cleavage  Cleavage | AT5G22080.1  AT5G22080.2 |  | Chaperone DnaJ-domain superfamily protein |
|  |  | 2,0 | Cleavage | AT1G16120.1 | WAKL1 | Wall associated kinase-like 1 |
|  |  | 2,0 | Cleavage | AT4G26120.1 |  | Ankyrin repeat family protein / BTB/POZ domain-containing protein |
|  |  | 2,5  2,5 | Cleavage  Cleavage | AT5G27395.1  AT5G27395.2 |  | Mitochondrial inner membrane translocase complex, subunit Tim44-related protein |
|  |  | 2,5  2,5 | Cleavage  Cleavage | AT1G19390.1  AT1G17910.1 |  | Wall-associated kinase family protein |
|  |  | 2,5 | Cleavage | AT3G47580.1 |  | Leucine-rich repeat protein kinase family protein |
|  |  | 2,5 | Translation | AT1G79680.1 | WAKL10, ATWAKL10 | WALL ASSOCIATED KINASE (WAK)-LIKE 10 |
|  |  | 2,5 | Cleavage | AT4G22592.1 | CPuORF27 | Conserved peptide upstream open reading frame 27 |
|  |  | 2,5 | Cleavage | AT3G19270.1 | CYP707A4 | Cytochrome P450, family 707, subfamily A, polypeptide 4 |
|  |  | 3,0 | Cleavage | AT1G42550.1 | PMI1 | Plastid movement impaired1 |
| miR160a-3p | CDP-P | 3,0  3,0 | Cleavage  Cleavage | AT1G08845.1  AT1G08845.2 |  | Ribosomal L18p/L5e family protein |
|  |  | 3,0  3,0 | Cleavage  Cleavage | AT3G51490.1  AT3G51490.2 | TMT3 | Tonoplast monosaccharide transporter3 |
|  |  | 3,5  3,5 | Cleavage  Cleavage | AT3G47010.1  AT3G47010.2 |  | Glycosyl hydrolase family protein |
|  |  | 3,5 | Cleavage | AT3G19710.1 | BCAT4 | Branched-chain aminotransferase4 |
|  |  | 3,5  3,5 | Cleavage  Cleavage | AT4G33300.1  AT4G33300.1 | ADR1-L1 | ADR1-like 1 |
|  |  | 3,5  3,5 | Translation  Translation | AT1G80410.1  AT1G80410.2 | EMB2753 | Tetratricopeptide repeat (TPR)-containing protein |
|  |  | 3,5 | Cleavage | AT1G60020.1 |  | Transposable element gene |
|  |  | 3,5 | Cleavage | AT1G64600.1 |  | Methyltransferases;copper ion binding |
|  |  | 4,0 | Translation | AT4G26600.1 |  | S-adenosyl-L-methionine-dependent methyltransferases superfamily protein |
|  |  | 4,0 | Cleavage | AT1G27940.1 | PGP13 | P-glycoprotein 13 |
| miR160c-3p | CDP-P | 2,5 | Cleavage | AT4G00740.1 |  | S-adenosyl-L-methionine-dependent methyltransferases superfamily protein |
|  |  | 2,5 | Translation | AT5G35073.1 |  | Transposable element gene |
|  |  | 3,0  3,0 | Cleavage  Cleavage | AT5G42370.1  AT5G42370.2 |  | Calcineurin-like metallo-phosphoesterase superfamily protein |
|  |  | 3,0  3,0 | Cleavage  Cleavage | AT4G33300.1  AT4G33300.2 | ADR1-L1 | ADR1-like 1 |
|  |  | 3,0  3,0  3,0 | Cleavage  Cleavage  Cleavage | AT3G04600.1  AT3G04600.2  AT3G04600.3 |  | Nucleotidylyl transferase superfamily protein |
|  |  | 3,0  3,0 | Cleavage  Cleavage | AT2G25320.1  AT2G25330.1 |  | TRAF-like family protein |
|  |  | 3,0 | Cleavage | AT1G70210.1 | CYCD1;1, ATCYCD1;1 | CYCLIN D1;1 |
|  |  | 3,5 | Cleavage | AT3G24610.1 |  | Galactose oxidase/kelch repeat superfamily protein |
|  |  | 3,5 | Cleavage | AT3G49950.1 |  | GRAS family transcription factor |
|  |  | 3,5 | Cleavage | AT5G61650.1 | CYCP4;2, CYCP4 | CYCLIN P4;2 |
| miR163 | CDP-P  CDP-L | 1,0  1,0  1,0  1,5 | Cleavage  Cleavage  Cleavage  Cleavage | AT5G38100.1  AT5G38100.2  AT1G15125.1  AT3G44840.1 |  | S-adenosyl-L-methionine-dependent methyltransferases superfamily protein |
|  |  | 1,5 | Cleavage | AT5G35630.1 | GS2, GLN2, ATGSL1 | Glutamine synthetase 2 |
|  |  | 2,0  2,0 | Cleavage  Cleavage | AT1G66700.3  AT1G66700.1 | PXMT1 | S-adenosyl-L-methionine-dependent methyltransferases superfamily protein |
|  |  | 2,0  2,0 | Cleavage  Cleavage | ATCG00880.1  ATCG01260.1 | TRNL.2 | tRNA-Leu |
|  |  | 2,5 | Cleavage | AT5G15810.1 |  | N2,N2-dimethylguanosine tRNA methyltransferase |
|  |  | 2,5  2,5 | Cleavage  Cleavage | AT2G12480.2  AT2G12480.1 | SCPL43 | Serine carboxypeptidase-like 43 |
|  |  | 2,5 | Cleavage | AT1G17000.1 | ATTPS3, TPS3 | Trehalose-phosphatase/synthase 3 |
|  |  | 2,5 | Cleavage | AT2G45900.1 |  | Phosphatidylinositol N-acetyglucosaminlytransferase subunit P-related |
|  |  | 3,0 | Translation | AT1G54990.1 | AXR4, RGR, RGR1 | Alpha/beta-Hydrolases superfamily protein |
|  |  | 3,5 | Translation | AT2G13440.1 |  | Glucose-inhibited division family A protein |
| miR164b-3p | P-L  CDP-L | 2,0 | Cleavage | AT5G14770.1 |  | Tetratricopeptide repeat (TPR)-like superfamily protein |
|  |  | 2,5  3,5 | Cleavage  Cleavage | AT2G09870.1  AT5G28935.1 |  | Transposable element gene |
|  |  | 3,0  3,0  3,0 | Cleavage  Cleavage  Cleavage | AT3G08840.1  AT3G08840.2  AT3G08840.3 |  | D-alanine-D-alanine ligase family |
|  |  | 3,0  3,0 | Cleavage  Cleavage | AT3G15940.1  AT3G15940.1 |  | UDP-Glycosyltransferase superfamily protein |
|  |  | 3,0 | Cleavage | AT1G32750.1 | HAF01, HAF1, HAC13, GTD1, TAF1 | HAC13 protein (HAC13) |
|  |  | 3,5  3,5 | Cleavage  Cleavage | AT5G07530.1  AT5G07530.2 | GRP17, ATGRP17, ATGRP-7 | Glycine rich protein 17 |
|  |  | 3,5 | Cleavage | AT3G51150.1 |  | ATP binding microtubule motor family protein |
|  |  | 3,5 | Cleavage | AT1G04210.1 |  | Leucine-rich repeat protein kinase family protein |
|  |  | 3,5  3,5  3,5  3,5 | Cleavage  Cleavage  Cleavage  Cleavage | AT5G63470.1 AT5G63470.2 AT1G54830.2  AT1G54830.3 | NF-YC4 | Nuclear factor Y, subunit C4 |
|  |  | 4,0 | Cleavage | AT3G05580.1 |  | Calcineurin-like metallo-phosphoesterase superfamily protein |
| miR165a-5p | CDP-L | 2,0 | Cleavage | AT1G34575.1 |  | FAD-binding Berberine family protein |
|  |  | 2,5  2,5 | Cleavage  Cleavage | AT3G48580.2  AT3G48580.1 | XTH11 | Xyloglucan endotransglucosylase/hydrolase 11 |
|  |  | 2,5 | Cleavage | AT1G43640.1 | AtTLP5, TLP5 | Tubby like protein 5 |
|  |  | 3,0 | Cleavage | AT1G24050.1 |  | RNA-processing, Lsm domain |
|  |  | 3,0 | Cleavage | AT5G52620.1 |  | F-box associated ubiquitination effector family protein |
|  |  | 3,0 | Cleavage | AT5G57035.1 |  | U-box domain-containing protein kinase family protein |
|  |  | 3,0  3,5  3,5 | Cleavage  Translation  Translation | AT1G19630.1  AT4G39500.1  AT4G32170.1 | CYP722A1 | Cytochrome P450, family 722, subfamily A, polypeptide 1 |
|  |  | 3,5 | Cleavage | AT5G40010.1 | AATP1 | AAA-ATPase 1 |
|  |  | 3,5 | Cleavage | AT5G54690.1 | GAUT12, LGT6, IRX8 | Galacturonosyltransferase 12 |
|  |  | 3,5 | Cleavage | AT4G13080.1 | XTH1 | Xyloglucan endotransglucosylase/hydrolase 1 |
| miR166a-5p  miR166b-5p | CDP-L  CDP-L | 2,5  3,0  3,0 | Cleavage  Cleavage  Cleavage | AT3G05380.2  AT3G05380.1  AT3G05380.3 | ALY2, ATALY2 | DIRP;Myb-like DNA-binding domain |
|  |  | 3,0  3,0 | Cleavage  Cleavage | AT3G05380.4  AT3G05380.5 | ALY2 | DIRP ;Myb-like DNA-binding domain |
|  |  | 3,5 | Cleavage | AT4G17950.1 |  | AT hook motif DNA-binding family protein |
|  |  | 3,5 | Cleavage | AT4G03080.1 | BSL1 | BRI1 suppressor 1 (BSU1)-like 1 |
|  |  | 3,5 | Cleavage | AT5G09720.1 |  | Magnesium transporter CorA-like family protein |
|  |  | 3,5 | Cleavage | AT5G58170.1 | SVL5 | SHV3-like 5 |
|  |  | 4,0 | Translation | AT3G11020.1 | DREB2B, DREB2 | DRE/CRT-binding protein 2B |
|  |  | 4,0 | Cleavage | AT4G24510.1 | CER2, VC2, VC-2 | HXXXD-type acyl-transferase family protein |
|  |  | 4,0  4,0 | Cleavage  Cleavage | AT2G27150.1  AT2G27150.2 | AAO3, At-AO3, AOdelta, AtAAO3 | Abscisic aldehyde oxidase 3 |
|  |  | 4,0 | Translation | AT1G36180.2 | ACC2 | Acetyl-CoA carboxylase 2 |
| miR166e-5p | CDP-P | 2,0 | Cleavage | AT5G04240.1 | ELF6 | Zinc finger (C2H2 type) family protein / transcription factor jumonji (jmj) family protein |
|  |  | 3,0 | Cleavage | AT1G01040.2 | DCL1 | Dicer-like 1 |
|  |  | 3,0 | Cleavage | AT1G01040.1 | DCL1, CAF, SUS1, SIN1, ASU1, EMB76, EMB60, ATDCL1 | Dicer-like 1 |
|  |  | 3,0 | Cleavage | AT5G35670.1 | iqd33 | IQ-domain 33 |
|  |  | 3,0  3,0 | Cleavage  Cleavage | AT1G78420.1  AT1G78420.2 |  | RING/U-box superfamily protein |
|  |  | 3,0 | Cleavage | AT3G26400.1 | EIF4B1 | Eukaryotic translation initiation factor 4B1 |
|  |  | 3,0 | Cleavage | AT1G10550.1 | XTH33, XET | Xyloglucan:xyloglucosyl transferase 33 |
|  |  | 3,5 | Cleavage | AT5G23310.1 | FSD3 | Fe superoxide dismutase 3 |
|  |  | 3,5 | Cleavage | AT5G65970.1 | MLO10, ATMLO10 | Seven transmembrane MLO family protein |
|  |  | 3,5  3,5 | Cleavage  Cleavage | AT1G77800.1  AT1G77800.2 |  | PHD finger family protein |
| miR167a-5p  miR167b | CDP-P  CDP-L  CDP-P  CDP-L | 2,5 | Cleavage | AT5G41300.1 |  | Receptor-like protein kinase-related family protein |
|  |  | 3,0 | Cleavage | AT5G58590.1 | RANBP1 | RAN binding protein 1 |
|  |  | 3,5  3,5 | Cleavage  Cleavage | AT5G37020.1  AT5G37020.2 | ARF8, ATARF8 | Auxin response factor 8 |
|  |  | 3,5  3,5 | Cleavage  Cleavage | AT3G45310.1  AT3G45310.2 |  | Cysteine proteinases superfamily protein |
|  |  | 3,5  3,5 | Cleavage  Cleavage | AT1G30330.1  AT1G30330.2 | ARF6 | Auxin response factor 6 |
|  |  | 3,5 | Cleavage | AT3G07810.1 |  | RNA-binding (RRM/RBD/RNP motifs) family protein |
|  |  | 3,5 | Cleavage | AT1G36010.1 |  | Transposable element gene |
|  |  | 3,5  3,5  3,5  3,5 | Translation  Translation  Translation  Translation | AT1G08570.1  AT1G08570.2  AT1G08570.3  AT1G08570.4 | ACHT4 | Atypical CYS HIS rich thioredoxin 4 |
|  |  | 3,5 | Cleavage | AT1G02800.1 | ATCEL2, CEL2 | Cellulase 2 |
|  |  | 3,5 | Cleavage | AT5G35840.1 | PHYC | Phytochrome C |
| miR167c-5p | CDP-P | 2,0  2,0 | Cleavage  Cleavage | AT1G30330.1  AT1G30330.2 | ARF6 | Auxin response factor 6 |
|  |  | 2,0  2,0 | Cleavage  Cleavage | AT5G37020.1  AT5G37020.2 | ARF8, ATARF8 | Auxin response factor 8 |
|  |  | 2,5 | Cleavage | AT1G40075.1 |  | Transposable element gene |
|  |  | 3,5 | Cleavage | AT3G61300.1 |  | C2 calcium/lipid-binding plant phosphoribosyltransferase family protein |
|  |  | 3,5 | Cleavage | AT3G21810.1 |  | Zinc finger C-x8-C-x5-C-x3-H type family protein |
|  |  | 3,5 | Cleavage | AT5G41300.1 |  | Receptor-like protein kinase-related family protein |
|  |  | 3,5  3,5 | Cleavage  Cleavage | AT3G59000.1  AT3G59000.2 |  | F-box/RNI-like superfamily protein |
|  |  | 3,5 | Cleavage | AT2G35650.1 | ATCSLA07, CSLA07, ATCSLA7, CSLA7 | Cellulose synthase like |
|  |  | 3,5  3,5 | Cleavage  Cleavage | AT4G19490.1  AT4G19490.2 | ATVPS54, VPS54 | VPS54 |
|  |  | 3,5  3,5 | Cleavage  Cleavage | AT1G16010.1  AT1G16010.2 | MGT2, MRS2-1 | Magnesium transporter 2 |
| miR167d | CDP-P | 2,5 | Cleavage | AT5G41300.1 |  | Receptor-like protein kinase-related family protein |
|  |  | 3,0 | Cleavage | AT5G58590.1 | RANBP1 | RAN binding protein 1 |
|  |  | 3,5  3,5 | Cleavage  Cleavage | AT3G07810.1  AT3G07810.2 |  | RNA-binding (RRM/RBD/RNP motifs) family protein |
|  |  | 3,5 | Cleavage | AT5G37020.1 | ARF8, ATARF8 | Auxin response factor 8 |
|  |  | 3,5 | Cleavage | AT5G37020.2 | ARF8 | Auxin response factor 8 |
|  |  | 3,5  3,5 | Cleavage  Cleavage | AT1G30330.1  AT1G30330.2 | ARF6 | Auxin response factor 6 |
|  |  | 3,5 | Cleavage | AT3G01820.1 |  | P-loop containing nucleoside triphosphate hydrolases superfamily protein |
|  |  | 3,5  3,5 | Cleavage  Cleavage | AT3G45310.1  AT3G45310.2 |  | Cysteine proteinases superfamily protein |
|  |  | 3,5  3,5  3,5  3,5 | Translation  Translation  Translation  Translation | AT1G08570.1  AT1G08570.2  AT1G08570.3  AT1G08570.4 | ACHT4 | Atypical CYS HIS rich thioredoxin 4 |
|  |  | 3,5 | Cleavage | AT1G02800.1 | ATCEL2, CEL2 | Cellulase 2 |
| miR168a-3p | CDP-P  CDP-L | 2,5 | Cleavage | AT3G07195.1 |  | RPM1-interacting protein 4 (RIN4) family protein |
|  |  | 3,0 | Cleavage | AT1G70170.1 | MMP | Matrix metalloproteinase |
|  |  | 3,0 | Cleavage | AT3G59780.1 |  | Rhodanese/Cell cycle control phosphatase superfamily protein |
|  |  | 3,0 | Cleavage | AT3G02210.1 | COBL1 | COBRA-like protein 1 precursor |
|  |  | 3,5  3,5 | Cleavage  Cleavage | AT5G17780.1  AT5G17780.2 |  | Alpha/beta-Hydrolases superfamily protein |
|  |  | 3,5 | Cleavage | AT5G07140.1 |  | Protein kinase superfamily protein |
|  |  | 3,5 | Cleavage | AT5G24830.1 |  | Tetratricopeptide repeat (TPR)-like superfamily protein |
|  |  | 4,0 | Cleavage | AT5G06460.1 | ATUBA2, UBA 2 | Ubiquitin activating enzyme 2 |
|  |  | 4,0 | Cleavage | AT2G43290.1 | MSS3 | Calcium-binding EF-hand family protein |
|  |  | 4,5  4,5  4,5 | Cleavage  Cleavage  Cleavage | AT5G62000.1  AT5G62000.2  AT5G62000.3 | ARF2, ARF1-BP, HSS, ORE14 | Auxin response factor 2 |
| miR168b-3p | CDP-P  CDP-L | 3,0  3,0 | Translation  Translation | AT4G20400.1  AT4G20400.2 | JMJ14, PKDM7B | JUMONJI 14 |
|  |  | 3,0 | Cleavage | AT1G60050.1 |  | Nodulin MtN21 /EamA-like transporter family protein |
|  |  | 3,0 | Cleavage | AT3G07195.1 |  | RPM1-interacting protein 4 (RIN4) family protein |
|  |  | 3,5  3,5 | Cleavage  Cleavage | AT1G11180.1  AT1G11180.2 |  | Secretory carrier membrane protein (SCAMP) family protein |
|  |  | 3,5  3,5  3,5 | Cleavage  Cleavage  Cleavage | AT1G64625.1  AT1G64625.2  AT1G64625.3 |  | Serine/threonine-protein kinase WNK (With No Lysine)-related |
|  |  | 3,5  3,5  3,5 | Translation  Translation  Translation | AT3G33035.1  AT2G12390.1  AT4G07760.1 |  | Transposable element gene |
|  |  | 3,5 | Cleavage | AT2G36660.1 | PAB7 | Poly(A) binding protein 7 |
|  |  | 4,0 | Cleavage | AT5G24910.1 | CYP714A1 | cytochrome P450, family 714, subfamily A |
|  |  | 4,0 | Cleavage | AT3G24520.1 | AT-HSFC1, HSFC1 | Heat shock transcription factor C1 |
|  |  | 4,0 | Cleavage | AT1G70170.1 | MMP | Matrix metalloproteinase |
| miR170-3p | CDP-P | 1,0 | Cleavage | AT4G00150.1 | HAM3, ATHAM3, LOM3 | GRAS family transcription factor |
|  |  | 1,0 | Cleavage | AT3G60630.1 | HAM2, ATHAM2, LOM2 | GRAS family transcription factor |
|  |  | 1,0 | Cleavage | AT2G45160.1 | HAM1, ATHAM1, LOM1 | GRAS family transcription factor |
|  |  | 3,0 | Cleavage | AT3G47170.1 |  | HXXXD-type acyl-transferase family protein |
|  |  | 3,0 | Cleavage | AT5G07920.1 | DGK1, ATDGK1 | Diacylglycerol kinase1 |
|  |  | 3,5 | Translation | AT3G55410.1 |  | 2-oxoglutarate dehydrogenase, E1 component |
|  |  | 3,5  3,5  3,5 | Translation  Translation  Translation | AT2G27920.1  AT2G27920.2  AT2G27920.3 | SCPL51 | Serine carboxypeptidase-like 51 |
|  |  | 3.5  3.5 | Translation  Translation | AT1G10340.1  AT1G10340.2 |  | Ankyrin repeat family protein |
|  |  | 3,5 | Cleavage | AT1G35940.1 |  | Transposable element gene |
|  |  | 3,5  3,5 | Cleavage  Cleavage | AT4G31900.1  AT4G31900.2 | PKR2 | Chromatin remodeling factor, putative |
| miR171a-3p | CDP-P  CDP-L | 0,0 | Cleavage | AT4G00150.1 | HAM3, ATHAM3, LOM3 | GRAS family transcription factor |
|  |  | 0,0 | Cleavage | AT3G60630.1 | HAM2, ATHAM2, LOM2 | GRAS family transcription factor |
|  |  | 0,0 | Cleavage | AT2G45160.1 | HAM1, ATHAM1, LOM1 | GRAS family transcription factor |
|  |  | 3,0 | Cleavage | AT4G11270.1 |  | Transducin/WD40 repeat-like superfamily protein |
|  |  | 3,5 | Cleavage | AT5G14720.1 |  | Protein kinase superfamily protein |
|  |  | 3,5 | Cleavage | AT2G02110.1 |  | Transposable element gene |
|  |  | 3,5 | Cleavage | AT3G50400.1 |  | GDSL-like Lipase/Acylhydrolase superfamily protein |
|  |  | 4,0 | Cleavage | AT3G47170.1 |  | HXXXD-type acyl-transferase family protein |
|  |  | 4,0 | Translation | AT3G05530.1 | RPT5A, ATS6A.2 | Regulatory particle triple-A ATPase 5A |
|  |  | 4,0  4,0 | Cleavage  Cleavage | AT2G03280.1  AT2G03280.2 |  | O-fucosyltransferase family protein |
| miR171b-5p | CDP-P | 2,5 | Cleavage | AT3G45860.1 | CRK4 | Cysteine-rich RLK (RECEPTOR-like protein kinase) 4 |
|  |  | 2,5 | Cleavage | AT1G71460.1 |  | Pentatricopeptide repeat (PPR-like) superfamily protein |
|  |  | 3,0 | Cleavage | AT4G21990.2 | APR3 | APS reductase 3 |
|  |  | 3,0 | Cleavage | AT4G21990.1 | APR3, PRH-26, PRH26, ATAPR3 | APS reductase 3 |
|  |  | 3,0 | Cleavage | AT4G08890.1 |  | Transposable element gene |
|  |  | 3,0  3,0 | Cleavage  Cleavage | AT1G03960.1  AT1G03960.2 |  | Calcium-binding EF hand family protein |
|  |  | 3,5 | Cleavage | AT1G73300.1 | scpl2 | Serine carboxypeptidase-like 2 |
|  |  | 3,5 | Cleavage | AT1G73270.1 | scpl6 | Serine carboxypeptidase-like 6 |
|  |  | 3,5 | Cleavage | AT5G36180.1 | scpl1 | Serine carboxypeptidase-like 1 |
|  |  | 3,5 | Cleavage | AT3G22800.1 |  | Leucine-rich repeat (LRR) family protein |
| miR172e-3p | CDP-L | 0,5 | Cleavage | AT3G54990.1 |  | Integrase-type DNA-binding superfamily protein |
|  |  | 1,0  1,0 | Cleavage  Cleavage | AT5G60120.1  AT5G60120.2 | TOE2 | Target of early activation tagged (EAT) 2 |
|  |  | 1,0 | Cleavage | AT5G67180.1 | TOE3 | Target of early activation tagged (EAT) 3 |
|  |  | 1,0 | Cleavage | AT2G39250.1 | SNZ | Integrase-type DNA-binding superfamily protein |
|  |  | 1,0 | Cleavage | AT4G36920.1 | AP2, FLO2, FL1 | Integrase-type DNA-binding superfamily protein |
|  |  | 1,0 | Cleavage | AT4G36920.2 | AP2 | Integrase-type DNA-binding superfamily protein |
|  |  | 1,0  1,0 | Cleavage  Cleavage | AT2G28550.1  AT2G28550.3 | RAP2.7, TOE1 | Related to AP2.7 |
|  |  | 2,5 | Cleavage | AT3G62240.1 |  | RING/U-box superfamily protein |
|  |  | 2,5 | Cleavage | AT5G65790.1 | ATMYB68, MYB68 | Myb domain protein 68 |
|  |  | 2,5  2,5 | Cleavage  Cleavage | AT1G05000.1  AT1G05000.2 |  | Phosphotyrosine protein phosphatases superfamily protein |
| miR172e-5p | CDP-L | 2,5 | Translation | AT2G16500.1 | ADC1, ARGDC1, ARGDC, SPE1 | Arginine decarboxylase 1 |
|  |  | 2,5 | Cleavage | AT5G48410.1 | ATGLR1.3, GLR1.3 | Glutamate receptor 1.3 |
|  |  | 3,0 | Cleavage | AT2G22950.1 |  | Cation transporter/ E1-E2 ATPase family protein |
|  |  | 3,0 | Cleavage | AT3G57330.1 | ACA11 | Autoinhibited Ca2+-ATPase 11 |
|  |  | 3,0 | Cleavage | AT2G03820.1 |  | Nonsense-mediated mRNA decay NMD3 family protein |
|  |  | 3,5 | Cleavage | AT3G07050.1 |  | GTP-binding family protein |
|  |  | 3,5 | Cleavage | AT1G79730.1 | ELF7 | Hydroxyproline-rich glycoprotein family protein |
|  |  | 0,5  0,5  1,0  1,0  1,0  1,0  1,0  1,0  1,0 | Cleavage  Cleavage  Cleavage  Cleavage  Cleavage  Cleavage  Cleavage  Cleavage  Cleavage | AT3G17265.1  AT5G36200.1  AT3G16820.1  AT3G16880.1  AT3G22350.1  AT3G17570.1  AT5G36730.1  AT5G36820.1  AT2G18780.1 |  | F-box and associated interaction domains-containing protein |
|  |  | 1,0  1,0 | Cleavage  Cleavage | AT3G22710.1  AT3G49510.1 |  | F-box family protein |
|  |  | 1,5 | Cleavage | AT3G14030.1 |  | F-box associated ubiquitination effector family protein |
|  |  | 2,5 | Cleavage | AT1G65990.1 |  | Type 2 peroxiredoxin-related / thiol specific antioxidant / mal allergen family protein |
|  |  | 2,5 | Cleavage | AT1G67830.1 | ATFXG1, FXG1 | Alpha-fucosidase 1 |
| miR173-3p | CDP-P  CDP-L | 2,5 | Cleavage | AT1G53020.1 | UBC26, PFU3 | UBC26 (UBIQUITIN-CONJUGATING ENZYME 26); ubiquitin-protein ligase |
|  |  | 2,5 | Cleavage | AT1G53025.1 |  | Ubiquitin-conjugating enzyme family protein |
|  |  | 2,5 | Cleavage | AT2G25240.1 |  | Serine protease inhibitor (SERPIN) family protein |
|  |  | 3,0 | Cleavage | AT2G13630.1 |  | F-box associated ubiquitination effector family protein |
|  |  | 3,0 | Cleavage | AT4G01883.1 |  | Polyketide cyclase / dehydrase and lipid transport protein |
|  |  | 3,0  3,0 | Cleavage  Cleavage | AT5G62620.1  AT5G62620.2 |  | Galactosyltransferase family protein |
|  |  | 3,0 | Cleavage | AT2G31830.1 |  | Endonuclease/exonuclease/phosphatase family protein |
|  |  | 3,0 | Cleavage | AT1G56500.1 |  | Haloacid dehalogenase-like hydrolase family protein |
|  |  | 3,0 | Translation | AT2G44000.1 |  | Late embryogenesis abundant (LEA) hydroxyproline-rich glycoprotein family |
|  |  | 3,5  3,5  3,5 | Cleavage  Cleavage  Cleavage | AT4G38960.1  AT4G38960.2  AT4G38960.3 |  | B-box type zinc finger family protein |
| miR319a | P-L  CDP-P  CDP-L | 1,5 | Cleavage | AT2G26950.1 | AtMYB104, MYB104 | Myb domain protein 104 |
|  |  | 1,5  1,5  1,5 | Cleavage  Cleavage  Cleavage | AT5G06100.1  AT5G06100.2  AT5G06100.3 | MYB33, ATMYB33 | Myb domain protein 33 |
|  |  | 1,5 | Cleavage | AT3G11440.1 | ATMYB65, MYB65 | Myb domain protein 65 |
|  |  | 2,5 | Cleavage | AT3G15030.1 | TCP4, MEE35 | TCP family transcription factor 4 |
|  |  | 2,5  2,5 | Cleavage  Cleavage | AT3G66658.1  AT3G66658.2 | LDH22A1 | Aldehyde dehydrogenase 22A1 |
|  |  | 2,5  2,5  2,5  2,5 | Cleavage  Cleavage  Cleavage  Cleavage | AT3G33076.1  AT4G22415.1  AT5G28335.1  AT3G33084.1 |  | Transposable element gene |
|  |  | 2,5 | Cleavage | AT1G48090.1 |  | Calcium-dependent lipid-binding family protein |
|  |  | 3,0 | Translation | AT2G29570.1 | PCNA2, ATPCNA2 | Proliferating cell nuclear antigen 2 |
|  |  | 3,0 | Translation | AT2G44450.1 | BGLU15 | Beta glucosidase 15 |
|  |  | 3,0 | Cleavage | AT1G53230.1 | TCP3 | TEOSINTE BRANCHED 1, cycloidea and PCF transcription factor 3 |
| miR319b  miR319c | P-L  CDP-P  CDP-L  CDP-P | 1,5 | Cleavage | AT2G26950.1 | AtMYB104, MYB104 | Myb domain protein 104 |
|  |  | 1,5  1,5  1,5 | Cleavage  Cleavage  Cleavage | AT5G06100.1  AT5G06100.2  AT5G06100.3 | MYB33, ATMYB33 | Myb domain protein 33 |
|  |  | 1,5 | Cleavage | AT3G11440.1 | ATMYB65, MYB65 | Myb domain protein 65 |
|  |  | 2,5 | Cleavage | AT2G31070.1 | TCP10 | TCP domain protein 10 |
|  |  | 2,5  2,5  2,5 | Cleavage  Cleavage  Cleavage | AT3G15030.1  AT3G15030.2  AT3G15030.3 | TCP4 | TCP family transcription factor 4 |
|  |  | 2,5  2,5 | Cleavage  Cleavage | AT3G66658.1  AT3G66658.2 | ALDH22A1 | Aldehyde dehydrogenase 22A1 |
|  |  | 2,5  2,5  2,5  2,5 | Cleavage  Cleavage  Cleavage  Cleavage | AT3G33076.1  AT4G22415.1  AT5G28335.1  AT3G33084.1 |  | Transposable element gene |
|  |  | 2,5  2,5 | Cleavage  Cleavage | AT1G30210.1  AT1G30210.2 | TCP24 | TEOSINTE BRANCHED 1, cycloidea, and PCF family 24 |
|  |  | 2,5  2,5 | Cleavage  Cleavage | AT4G18390.1  AT4G18390.2 | TCP2 | TEOSINTE BRANCHED 1, cycloidea and PCF transcription factor 2 |
|  |  | 2,5 | Cleavage | AT1G48090.1 |  | Calcium-dependent lipid-binding family protein |
|  |  | 3,0 | Cleavage | AT2G29570.1 | PCNA2, ATPCNA2 | Proliferating cell nuclear antigen 2 |
| miR390a-3p | CDP-L | 3,0 | Cleavage | AT1G58050.1 |  | RNA helicase family protein |
|  |  | 3,5 | Cleavage | AT2G05170.1 | ATVPS11, VPS11 | Vacuolar protein sorting 11 |
|  |  | 3,5  3,5  3,5  3,5  3,5  3,5 | Cleavage  Cleavage  Cleavage  Cleavage  Cleavage  Cleavage | AT3G42313.1  AT1G42605.1  AT2G14650.1  AT5G26236.1  AT2G13830.1  AT3G31970.1 |  | Transposable element gene |
|  |  | 3,5  3,5 | Cleavage  Cleavage | AT3G07810.1  AT3G07810.2 |  | RNA-binding (RRM/RBD/RNP motifs) family protein |
|  |  | 3,5 | Cleavage | AT4G39700.1 |  | Heavy metal transport/detoxification superfamily protein |
|  |  | 3,5 | Cleavage | AT1G72280.1 | AERO1, ERO1 | Endoplasmic reticulum oxidoreductins 1 |
|  |  | 4,0 | Cleavage | AT2G36990.1 | SIGF, SIG6, ATSIG6, SOLDAT8 | RNApolymerase sigma-subunit F |
|  |  | 4,0  4,0 | Translation  Translation | AT5G65670.1  AT5G65670.2 | IAA9 | indole-3-acetic acid inducible 9 |
|  |  | 4,0 | Cleavage | AT5G10530.1 |  | Concanavalin A-like lectin protein kinase family protein |
|  |  | 4,0 | Cleavage | AT1G36950.1 |  | RING/U-box superfamily protein |
| miR390a-5p  miR390b-5p | CDP-P  CDP-L  CDP-L | 2,5 | Cleavage | AT5G57735.1 | TASIR-ARF | tasiR-ARF; other RNA |
|  |  | 2,5 | Translation | AT5G49615.1 | TAS3b | TAS3b (trans-acting siRNA 3b); other RNA |
|  |  | 3,0  3,0  3,5  3,5 | Cleavage  Cleavage  Translation  Translation | AT3G17185.1  AT3G17185.2  AT3G17185.1  AT3G17185.1 | TASIR-ARF, TAS3, ATTAS3 | TAS3/TASIR-ARF (TRANS-ACTING SIRNA3); other RNA |
|  |  | 3,0 | Translation | AT5G03640.1 |  | Protein kinase superfamily protein |
|  |  | 3,0 | Cleavage | AT5G48480.1 |  | Lactoylglutathione lyase / glyoxalase I family protein |
|  |  | 3,0  3,0 | Cleavage  Cleavage | AT4G32820.1  AT4G32820.2 |  | Tetratricopeptide repeat (TPR)-like superfamily protein |
|  |  | 3,5  3,5 | Cleavage  Cleavage | AT5G05570.1  AT5G05570.2 |  | Transducin family protein / WD-40 repeat family protein |
|  |  | 3,5  3,5 | Cleavage  Cleavage | AT5G66530.1  AT5G66530.2 |  | Galactose mutarotase-like superfamily protein |
|  |  | 3,5 | Cleavage | AT3G24660.1 | TMKL1 | Transmembrane kinase-like 1 |
| miR390a-3p | CDP-P | 3,0 | Cleavage | AT1G58050.1 |  | RNA helicase family protein |
|  |  | 3,5 | Cleavage | AT2G05170.1 | ATVPS11, VPS11 | Vacuolar protein sorting 11 |
|  |  | 3,5  3,5  3,5  3,5 | Cleavage  Cleavage  Cleavage  Cleavage | AT3G42313.1  AT1G42605.1  AT2G14650.1  AT5G26236.1 |  | Transposable element gene |
|  |  | 3,5 | Cleavage | AT3G07810.1  AT3G07810.2 |  | RNA-binding (RRM/RBD/RNP motifs) family protein |
|  |  | 3,5 | Cleavage | AT4G39700.1 |  | Heavy metal transport/detoxification superfamily protein |
|  |  | 3,5 | Cleavage | AT1G72280.1 | AERO1, ERO1 | Endoplasmic reticulum oxidoreductins 1 |
|  |  | 4,0 | Cleavage | AT2G36990.1 | SIGF, SIG6, ATSIG6, SOLDAT8 | RNApolymerase sigma-subunit F |
|  |  | 4,0  4,0 | Translation  Translation | AT5G65670.1  AT5G65670.2 | IAA9 | Indole-3-acetic acid inducible 9 |
|  |  | 4,0 | Cleavage | AT5G10530.1 |  | Concanavalin A-like lectin protein kinase family protein |
|  |  | 4,0 | Cleavage | AT1G36950.1 |  | RING/U-box superfamily protein |
| miR393a-3p | CDP-P  CDP-L | 2,0  2,0  2,0 | Cleavage  Cleavage  Cleavage | AT1G26850.1  AT1G26850.2  AT1G26850.3 |  | S-adenosyl-L-methionine-dependent methyltransferases superfamily protein |
|  |  | 3,0 | Cleavage | AT5G65930.3 | ZWI | Kinesin-like calmodulin-binding protein (ZWICHEL) |
|  |  | 3,0  3,0 | Cleavage  Cleavage | AT5G65930.1  AT5G65930.2 | ZWI, PKCBP, KCBP | Kinesin-like calmodulin-binding protein (ZWICHEL) |
|  |  | 3,0 | Cleavage | AT3G19420.1 | ATPEN2, PEN2 | PTEN 2 |
|  |  | 3,5  3,5 | Cleavage  Cleavage | AT3G13040.1  AT3G13040.2 |  | Myb-like HTH transcriptional regulator family protein |
|  |  | 3,5  3,5 | Cleavage  Cleavage | AT2G32415.1  AT2G32415.2 |  | Polynucleotidyl transferase, ribonuclease H fold protein with HRDC domain |
|  |  | 3,5 | Cleavage | AT4G30600.1 |  | Signal recognition particle receptor alpha subunit family protein |
|  |  | 3,5 | Cleavage | AT1G20720.1 |  | RAD3-like DNA-binding helicase protein |
|  |  | 4,0 | Cleavage | AT4G16550.1 |  | HSP20-like chaperone |
|  |  | 4,0 | Translation | AT5G49570.1 | AtPNG1, PNG1 | Peptide-N-glycanase 1 |
| miR393b-3p | CDP-P | 2,0 | Cleavage | AT3G19420.1 | ATPEN2, PEN2 | PTEN 2 |
|  |  | 2,5 | Translation | AT3G59740.1 |  | Concanavalin A-like lectin protein kinase family protein |
|  |  | 2,5  2,5 | Cleavage  Cleavage | AT3G63180.1  AT3G63180.2 | ATTKL, TKL | TIC-like |
|  |  | 2,5  3,0 | Cleavage  Cleavage | AT5G34770.1  AT3G43358.1 |  | Transposable element gene |
|  |  | 3,0 | Cleavage | AT5G42820.2 | ATU2AF35B, U2AF35B | Zinc finger C-x8-C-x5-C-x3-H type family protein |
|  |  | 3,0  3,0 | Cleavage  Cleavage | AT3G13225.1  AT3G13225.2 |  | WW domain-containing protein |
|  |  | 3,5  3,5 | Cleavage  Cleavage | AT4G09040.1  AT4G09040.2 |  | RNA-binding (RRM/RBD/RNP motifs) family protein |
|  |  | 3,5  3,5 | Translation  Translation | AT3G05510.1  AT3G05510.2 |  | Phospholipid/glycerol acyltransferase family protein |
|  |  | 3,5 | Cleavage | AT1G56670.1 |  | GDSL-like Lipase/Acylhydrolase superfamily protein |
|  |  | 3,5 | Cleavage | AT5G08590.1 | ASK2, SNRK2-1, SNRK2.1, SRK2G | SNF1-related protein kinase 2.1 |
| miR395a | CDP-P | 1,5 | Cleavage | AT5G43780.1 | APS4 | Pseudouridine synthase/archaeosine transglycosylase-like family protein |
|  |  | 1,5 | Cleavage | AT5G10180.1 | AST68, SULTR2;1 | Slufate transporter 2;1 |
|  |  | 2,0  2,0 | Cleavage  Cleavage | AT5G13630.1  AT5G13630.2 | GUN5, CCH, CHLH, CCH1, ABAR | Magnesium-chelatase subunit chlH, chloroplast, putative / Mg-protoporphyrin IX chelatase, putative (CHLH) |
|  |  | 2,5 | Cleavage | AT4G23990.1 | ATCSLG3, CSLG3 | Cellulose synthase like G3 |
|  |  | 3,0 | Cleavage | AT4G14680.1 | APS3 | Pseudouridine synthase/archaeosine transglycosylase-like family protein |
|  |  | 3,0 | Cleavage | AT3G22890.1 | APS1 | ATP sulfurylase 1 |
|  |  | 3,0 | Cleavage | AT4G23840.1 |  | Leucine-rich repeat (LRR) family protein |
|  |  | 3,0  3,0 | Cleavage  Cleavage | AT3G29078.1  AT5G12085.1 |  | Transposable element gene |
|  |  | 3,5 | Cleavage | AT3G09220.1 | LAC7 | Laccase 7 |
|  |  | 3,5 | Cleavage | AT2G20463.1 |  | Defensin-like (DEFL) family protein |
| miR395b  miR395f | P-L  P-L | 2,0 | Cleavage | AT5G43780.1 | APS4 | Pseudouridine synthase/archaeosine transglycosylase-like family protein |
|  |  | 2,0 | Cleavage | AT5G10180.1 | AST68, SULTR2;1 | Slufate transporter 2;1 |
|  |  | 2,5  2,5 | Cleavage  Cleavage | AT5G13630.1  AT5G13630.2 | GUN5, CCH, CHLH, CCH1, ABAR | Magnesium-chelatase subunit chlH, chloroplast, putative / Mg-protoporphyrin IX chelatase, putative (CHLH) |
|  |  | 3,0 | Cleavage | AT4G23990.1 | ATCSLG3, CSLG3 | Cellulose synthase like G3 |
|  |  | 3,0  3,0 | Cleavage  Cleavage | AT5G65140.1  AT5G65140.1 | TPPJ | Haloacid dehalogenase-like hydrolase (HAD) superfamily protein |
|  |  | 3,5 | Cleavage | AT4G14680.1 | APS3 | Pseudouridine synthase/archaeosine transglycosylase-like family protein |
|  |  | 3,5  3,5  3,5  3,5 | Cleavage  Cleavage  Cleavage  Cleavage | AT2G10820.1  AT4G06547.1  AT3G29078.1  AT5G31927.1 |  | Transposable element gene |
|  |  | 3,5 | Cleavage | AT1G66400.1 | CML23 | Calmodulin like 23 |
|  |  | 3,5  3,5 | Cleavage  Cleavage | AT3G57300.1 AT3G57300.2 | INO80, ATINO80 | INO80 ortholog |
|  |  | 3,5  3,5 | Cleavage  Cleavage | AT5G22080.1  AT5G22080.2 |  | Chaperone DnaJ-domain superfamily protein |
| miR396a-3p | CDP-P  CDP-L | 2,5 | Cleavage | AT3G54280.1 | CHR16, CHA16, RGD3, ATBTAF1, BTAF1 | DNA binding;ATP binding;nucleic acid binding;binding;helicases;ATP binding;DNA binding;helicases |
|  |  | 2,5 | Cleavage | AT3G54280.2 | RGD3 | DNA binding;ATP binding;nucleic acid binding;binding;helicases;ATP binding;DNA binding;helicases |
|  |  | 2,5  2,5 | Cleavage  Cleavage | AT2G46060.1  AT2G46060.2 |  | Transmembrane protein-related |
|  |  | 2,5 | Cleavage | AT1G08290.1 | WIP3 | WIP domain protein 3 |
|  |  | 3,0  3,0  3,0  3,0 | Cleavage  Cleavage  Cleavage  Cleavage | AT5G25560.1  AT5G25560.2  AT5G25560.3  AT5G25560.4 |  | CHY-type/CTCHY-type/RING-type Zinc finger protein |
|  |  | 3,5 | Cleavage | AT5G18320.1 |  | ARM repeat superfamily protein |
|  |  | 3,5  3,5 | Cleavage  Cleavage | AT5G64250.1  AT5G64250.2 |  | Aldolase-type TIM barrel family protein |
|  |  | 3,5 | Cleavage | AT2G04378.1 |  | Beta-galactosidase related protein |
|  |  | 3,5 | Cleavage | AT1G67120.1 |  | ATPases;nucleotide binding;ATP binding;nucleoside-triphosphatases;transcription factor binding |
|  |  | 3,5  3,5 | Cleavage  Cleavage | AT1G53710.1  AT1G53710.1 |  | Calcineurin-like metallo-phosphoesterase superfamily protein |
| miR396b-5p | CDP-P  CDP-L | 1,5 | Cleavage | AT5G01370.1 | ACI1 | ALC-interacting protein 1 |
|  |  | 2,0 | Cleavage | AT5G43060.1 |  | Granulin repeat cysteine protease family protein |
|  |  | 2,0 | Cleavage | AT2G15630.1 |  | Pentatricopeptide repeat (PPR) superfamily protein |
|  |  | 2,5 | Cleavage | AT5G57590.1 | BIO1 | Adenosylmethionine-8-amino-7-oxononanoate transaminases |
|  |  | 2,5  2,5  2,5  2,5  2,5 | Cleavage Cleavage  Cleavage  Cleavage  Cleavage | AT1G58020.1  AT1G60120.1  AT4G28960.1  AT2G23710.1  AT5G44415.1 |  | Transposable element gene |
|  |  | 2,5 | Cleavage | AT3G19040.1 | TAF1, TAF1B, HAF2 | Histone acetyltransferase of the TAFII250 family 2 |
|  |  | 2,5 | Cleavage | AT1G60140.1 | ATTPS10, TPS10 | Trehalose phosphate synthase |
|  |  | 3,0 | Cleavage | AT2G22840.1 | AtGRF1, GRF1 | Growth-regulating factor 1 |
|  |  | 3,0 | Cleavage | AT4G37740.1 | AtGRF2, GRF2 | Growth-regulating factor 2 |
|  |  | 3,0 | Cleavage | AT2G36400.1 | AtGRF3, GRF3 | Growth-regulating factor 3 |
|  |  | 3,0 | Cleavage | AT2G45480.1 | AtGRF9, GRF9 | Growth-regulating factor 9 |
|  |  | 3,0 | Cleavage | AT3G52910.1 | AtGRF4, GRF4 | Growth-regulating factor 4 |
|  |  | 3,0 | Cleavage | AT5G53660.1 | AtGRF7, GRF7 | Growth-regulating factor 7 |
|  |  | 3,0 | Cleavage | AT4G24150.1 | AtGRF8, GRF8 | Growth-regulating factor 8 |
|  |  | 3,0 | Cleavage | AT3G19400.2 |  | Cysteine proteinases superfamily protein |
|  |  | 3,0  2,5  2,5 | Translation  Translation  Translation | AT3G14110.1  AT3G14110.2  AT3G14110.3 |  | Tetratricopeptide repeat (TPR)-like superfamily protein |
|  |  | 3,0 | Cleavage | AT5G58980.1 |  | Neutral/alkaline non-lysosomal ceramidase |
| miR397a | P-L  CDP-L | 0,5 | Cleavage | AT2G29130.1 | LAC2, ATLAC2 | Laccase 2 |
|  |  | 0,5 | Cleavage | AT2G38080.1 | IRX12, LAC4, ATLMCO4, LMCO4 | Laccase/Diphenol oxidase family protein |
|  |  | 1,0 | Cleavage | AT5G60020.1 | LAC17, ATLAC17 | Laccase 17 |
|  |  | 2,0 | Cleavage | AT3G06470.1 |  | GNS1/SUR4 membrane protein family |
|  |  | 2,5  2,5 | Cleavage  Cleavage | AT3G17880.1  AT3G17880.2 | HIP, ATTDX, ATHIP2, TDX | Tetraticopeptide domain-containing thioredoxin |
|  |  | 3,0 | Cleavage | AT4G33230.1 |  | Plant invertase/pectin methylesterase inhibitor superfamily |
|  |  | 3,0 | Cleavage | AT1G20950.1 |  | Phosphofructokinase family protein |
|  |  | 3,0  3,0  3,0 | Cleavage  Cleavage  Cleavage | AT3G06040.1  AT3G06040.2  AT3G06040.3 |  | Ribosomal protein L12/ ATP-dependent Clp protease adaptor protein ClpS family protein |
|  |  | 3,0  3,0 | Cleavage  Cleavage | AT1G79920.1  AT1G79920.2 |  | Heat shock protein 70 (Hsp 70) family protein |
|  |  | 3,0  3,0 | Cleavage  Cleavage | AT1G79930.1  AT1G79930.2 | HSP91 | Heat shock protein 91 |
| miR397b | CDP-L | 1,5  1,5 | Cleavage  Cleavage | AT3G60250.1  AT3G60250.2 | CKB3 | Casein kinase II beta chain 3 |
|  |  | 2,0 | Cleavage | AT2G29130.1 | LAC2, ATLAC2 | Laccase 2 |
|  |  | 2,0 | Cleavage | AT2G38080.1 | IRX12, LAC4, ATLMCO4, LMCO4 | Laccase/Diphenol oxidase family protein |
|  |  | 2,5  2,5  2,5 | Cleavage  Cleavage  Cleavage | AT5G02470.1  AT5G02470.2  AT5G02470.3 |  | Transcription factor DP |
|  |  | 2,5 | Cleavage | AT5G41300.1 |  | Receptor-like protein kinase-related family protein |
|  |  | 3,0 | Cleavage | AT1G79600.1 |  | Protein kinase superfamily protein |
|  |  | 3,0 | Cleavage | AT3G24640.1 |  | Lyases |
|  |  | 3,0 | Cleavage | AT1G21160.1 |  | Eukaryotic translation initiation factor 2 (eIF-2) family protein |
|  |  | 3,0 | Translation | AT4G34480.1 |  | O-Glycosyl hydrolases family 17 protein |
|  |  | 3,0 | Cleavage | AT1G19360.1 |  | Nucleotide-diphospho-sugar transferase family protein |
| miR398a-3p  miR398b-3p  miR398c-3p | CDP-L  P-L  CDP-P  CDP-L  P-L  CDP-P  CDP-L | 0,0 | Cleavage | AT5G14550.1 |  | Core-2/I-branching beta-1,6-N-acetylglucosaminyltransferase family protein |
|  |  | 3,0 | Cleavage | AT3G06370.1 | NHX4, ATNHX4 | Sodium hydrogen exchanger 4 |
|  |  | 3,0 | Cleavage | AT3G43860.1 | AtGH9A4, GH9A4 | Glycosyl hydrolase 9A4 |
|  |  | 3,5 | Translation | AT4G26230.1 |  | Ribosomal protein L31e family protein |
|  |  | 3,5 | Cleavage | AT4G11250.1 | AGL52 | AGAMOUS-like 52 |
|  |  | 3,5 | Cleavage | AT2G33410.1 |  | RNA-binding (RRM/RBD/RNP motifs) family protein |
|  |  | 3,5 | Cleavage | AT4G32320.1 | APX6 | Ascorbate peroxidase 6 |
|  |  | 3,5  3,5  3,5 | Cleavage  Cleavage  Cleavage | AT1G12520.1  AT1G12520.2  AT1G12520.3 | ATCCS, CCS | Copper chaperone for SOD1 |
|  |  | 3,5 | Cleavage | AT2G39850.1 |  | Subtilisin-like serine endopeptidase family protein |
|  |  | 4,0 | Cleavage | AT1G08830.1 | CSD1 | Copper/zinc superoxide dismutase 1 |
|  |  | 5,0 | Cleavage | AT2G28190.1 | CSD2, CZSOD2 | Copper/zinc superoxide dismutase 2 |
| miR398a-5p | CDP-P  CDP-L | 2,0  2,0  2,0  2,0 | Cleavage  Cleavage  Cleavage  Cleavage | AT5G06120.1  AT5G06120.2  AT5G06120.3  AT5G06120.4 |  | ARM repeat superfamily protein |
|  |  | 2,5 | Cleavage | AT1G15520.1 | PDR12, ATPDR12, ABCG40, ATABCG40 | Pleiotropic drug resistance 12 |
|  |  | 3,0 | Cleavage | AT4G09680.1 | CTC1, ATCTC1 | Conserved telomere maintenance component 1 |
|  |  | 3,0  3,0  3,0 | Cleavage  Cleavage  Cleavage | AT1G04080.1  AT1G04080.2  AT1G04080.3 | PRP39 | Tetratricopeptide repeat (TPR)-like superfamily protein |
|  |  | 3,5 | Cleavage | AT3G03540.1 | NPC5 | Non-specific phospholipase C5 |
|  |  | 3,5 | Translation | AT1G74580.1 |  | Pentatricopeptide repeat (PPR) superfamily protein |
|  |  | 3,5 | Cleavage | AT2G27480.1 |  | Calcium-binding EF-hand family protein |
|  |  | 3,5 | Cleavage | AT2G03210.1 | FUT2, ATFUT2 | Fucosyltransferase 2 |
|  |  | 3,5 | Cleavage | AT1G25375.1 |  | Metallo-hydrolase/oxidoreductase superfamily protein |
|  |  | 4,0  4,0 | Translation  Translation | AT3G45100.1  AT3G45100.1 | SETH2 | UDP-Glycosyltransferase superfamily protein |
| miR398c-5p | CDP-L | 0,0 | Cleavage | AT5G14550.1 |  | Core-2/I-branching beta-1,6-N-acetylglucosaminyltransferase family protein |
|  |  | 1,5 | Cleavage | AT5G37910.1 |  | Protein with RING/U-box and TRAF-like domains |
|  |  | 2,0  2,5  2,5  2,5 | Cleavage  Cleavage  Cleavage  Cleavage | AT2G04070.1  AT2G04050.1  AT2G04100.1  AT2G04090.1 |  | MATE efflux family protein |
|  |  | 2,5 | Cleavage | AT1G78050.1 | PGM | Phosphoglycerate/bisphosphoglycerate mutase |
|  |  | 3,0 | Cleavage | AT1G20910.1 |  | ARID/BRIGHT DNA-binding domain-containing protein |
|  |  | 3,0 | Cleavage | AT1G21460.1 | SWEET1, AtSWEET1 | Nodulin MtN3 family protein |
|  |  | 3,0 | Cleavage | AT1G07745.2 | ATRAD51D, RAD51D, SSN1 | Homolog of RAD51 D |
|  |  | 3,5  3,5 | Cleavage  Cleavage | AT4G27050.1  AT4G27050.2 |  | F-box/RNI-like superfamily protein |
|  |  | 3,5 | Cleavage | AT5G48740.1 |  | Leucine-rich repeat protein Kinase family protein |
|  |  | 3,5 | Cleavage | AT2G01480.1 |  | O-fucosyltransferase family protein |
| miR399b  miR399c-3p | CDP-P  CDP-L  CDP-P  CDP-L | 1,5 | Cleavage | AT2G33770.1 | UBC24, ATUBC24, PHO2 | Phosphate 2 |
|  |  | 2,5 | Cleavage | AT3G06500.1 |  | Plant neutral invertase family protein |
|  |  | 3,0 | Cleavage | AT3G54700.1 | PHT1;7 | Phosphate transporter 1;7 |
|  |  | 3,0 | Cleavage | AT5G27630.1 | ACBP5 | Acyl-CoA binding protein 5 |
|  |  | 3,0  3,0 | Cleavage  Cleavage | AT5G35210.2  AT5G35210.1 |  | Metalloendopeptidases; zinc ion binding; DNA binding |
|  |  | 3,0 | Cleavage | AT1G21270.1 | WAK2 | Wall-associated kinase 2 |
|  |  | 3,0 | Cleavage | AT1G56560.1 |  | Plant neutral invertase family protein |
|  |  | 3,0 | Cleavage | AT5G27630.1 | ACBP5 | Acyl-CoA binding protein 5 |
|  |  | 3,0 | Cleavage | AT4G00170.1 |  | Plant VAMP (vesicle-associated membrane protein) family protein |
|  |  | 3,5 | Cleavage | AT2G24696.1 |  | Transcriptional factor B3 family protein |
| miR399c-5p | P-L  CDP-P  CDP-L | 2,5  2,5 | Cleavage  Cleavage | AT5G64470.1  AT5G64470.2 | TBL12 | Plant protein of unknown function (DUF828) |
|  |  | 3,0  3,0 | Cleavage  Cleavage | AT5G36930.1  AT5G36930.2 |  | Disease resistance protein (TIR-NBS-LRR class) family |
|  |  | 3,0  3,0  3,0 | Cleavage  Cleavage  Cleavage | AT2G34410.1  AT2G34410.2 AT2G34410.3 |  | O-acetyltransferase family protein |
|  |  | 3,0 | Cleavage | AT3G54190.1 |  | Transducin/WD40 repeat-like superfamily protein |
|  |  | 3,5  3,5 | Translation  Translation | AT2G01630.2  AT2G01630.1 |  | O-Glycosyl hydrolases family 17 protein |
|  |  | 3,5 | Cleavage | AT5G06000.1 | EIF3G2, ATEIF3G2 | Eukaryotic translation initiation factor |
|  |  | 3,5 | Cleavage | AT5G04600.1 |  | RNA-binding (RRM/RBD/RNP motifs) family protein |
|  |  | 3,5  3,5  3,5 | Cleavage  Cleavage  Translation | AT2G01630.1  AT2G01630.2  AT1G66250.1 |  | O-Glycosyl hydrolases family 17 protein |
|  |  | 3,5 | Cleavage | AT1G61500.1 |  | S-locus lectin protein kinase family protein |
|  |  | 3,5 | Cleavage | AT5G10180.1 | AST68, SULTR2;1 | Slufate transporter 2;1 |
| miR399d  miR399f | CDP-P  CDP-L  CDP-P  CDP-L | 0,0  0,5  1,0 | Cleavage  Cleavage  Cleavage | AT2G33770.1  AT2G33770.1  AT2G33770.1 | UBC24, ATUBC24, PHO2 | Phosphate 2 |
|  |  | 2,0 | Cleavage | AT2G26900.1 |  | Sodium Bile acid symporter family |
|  |  | 2,5 | Cleavage | AT4G09730.1 | RH39 | RH39 |
|  |  | 3,0 | Cleavage | AT3G20940.1 | CYP705A30 | Cytochrome P450, family 705, subfamily A, polypeptide 30 |
|  |  | 3,0 | Cleavage | AT5G09730.1 | BXL3, ATBXL3, XYL3, ATBX3, BX3 | Beta-xylosidase 3 |
|  |  | 3,0 | Cleavage | AT1G78500.1 |  | Terpenoid cyclases family protein |
|  |  | 3,0 | Cleavage | AT5G36150.1 | ATPEN3, PEN3 | Putative pentacyclic triterpene synthase 3 |
|  |  | 3,5 | Cleavage | AT4G25960.1 | PGP2 | P-glycoprotein 2 |
|  |  | 4,0 | Cleavage | AT5G15660.1 |  | F-box and associated interaction domains-containing protein |
|  |  | 4,0  4,0 | Cleavage  Cleavage | AT1G05670.1  AT1G05670.2 |  | Pentatricopeptide repeat (PPR-like) superfamily protein |
| miR400 | CDP-P | 0,0  0,0  0,0 | Cleavage  Cleavage  Cleavage | AT1G06580.1  AT1G62720.1  AT3G22470.1 |  | Pentatricopeptide repeat (PPR) superfamily protein |
|  |  | 1,0  1,0  2,0  2,0 | Cleavage  Cleavage  Cleavage  Cleavage | AT4G19440.1  AT4G19440.2  AT1G63130.1  AT1G62930.1 |  | Tetratricopeptide repeat (TPR)-like superfamily protein |
|  |  | 2,0 | Cleavage | AT1G62670.1 | RPF2 | RNA processing factor 2 |
|  |  | 2,5 | Cleavage | AT5G39710.1 | EMB2745 | Tetratricopeptide repeat (TPR)-like superfamily protein |
|  |  | 2,5 | Cleavage | AT2G28680.1 |  | RmlC-like cupins superfamily protein |
|  |  | 3,0 | Cleavage | AT1G79500.5 | AtkdsA1 | Aldolase-type TIM barrel family protein |
|  |  | 3,0  3,0  3,0 | Cleavage  Cleavage  Cleavage | AT5G60360.1  AT5G60360.2  AT5G60360.3 | SAG2, AALP, ALP | Aleurain-like protease |
|  |  | 3,0 | Cleavage | AT2G05780.1 |  | Transposable element gene |
|  |  | 3,0 | Cleavage | AT1G64060.1 | ATRBOH F, ATRBOHF, RBOHAP108, RBOHF, RBOH F | Respiratory burst oxidase protein F |
|  |  | 3,0  3,0  3,0 | Cleavage  Cleavage  Cleavage | AT4G35790.1  AT4G35790.2  AT4G35790.3 | TPLDDELTA, PLDDELTA | Phospholipase D delta |
| miR406 | CDP-P  CDP-L | 2,0 | Cleavage | AT1G54380.1 |  | Spliceosome protein-related |
|  |  | 3,0 | Cleavage | AT1G03660.1 |  | Ankyrin-repeat containing protein |
|  |  | 3,0 | Cleavage | AT5G40340.1 |  | Tudor/PWWP/MBT superfamily protein |
|  |  | 3,0 | Cleavage | AT3G54980.1 |  | Pentatricopeptide repeat (PPR) superfamily protein |
|  |  | 3,5 | Cleavage | AT4G39770.1 | TPPH | Haloacid dehalogenase-like hydrolase (HAD) superfamily protein |
|  |  | 3,5 | Cleavage | AT3G24890.1 | ATVAMP728, VAMP728 | Vesicle-associated membrane protein 728 |
|  |  | 3,5 | Cleavage | AT1G06410.1 | ATTPS7, TPS7, ATTPSA | Trehalose-phosphatase/synthase 7 |
|  |  | 3,5 | Cleavage | AT2G18710.1 | SCY1 | SECY homolog 1 |
|  |  | 3,5 | Cleavage | AT4G23510.1 |  | Disease resistance protein (TIR-NBS-LRR class) family |
|  |  | 3,5  3,5 | Cleavage  Cleavage | AT1G43830.1  AT4G07938.1 |  | Transposable element gene |
| miR408-5p | P-L  CDP-L | 0,0  0,0 | Cleavage  Cleavage | AT2G47020.1  AT2G47020.2 |  | Peptide chain release factor 1 |
|  |  | 2,5 | Cleavage | AT4G03950.1 |  | Nucleotide/sugar transporter family protein |
|  |  | 2,5 | Cleavage | AT4G02940.1 |  | Oxidoreductase, 2OG-Fe(II) oxygenase family protein |
|  |  | 2,5  2,5 | Cleavage  Cleavage | AT1G67480.1  AT1G67480.2 |  | Galactose oxidase/kelch repeat superfamily protein |
|  |  | 2,5 | Cleavage | AT5G42150.1 |  | Glutathione S-transferase family protein |
|  |  | 2,5 | Cleavage | AT1G17180.1 | ATGSTU25, GSTU25 | Glutathione S-transferase TAU 25 |
|  |  | 3,0 | Cleavage | AT1G04210.1 |  | Leucine-rich repeat protein kinase family protein |
|  |  | 3,0 | Cleavage | AT5G10660.1 |  | Calmodulin-binding protein-related |
|  |  | 3,0 | Cleavage | AT2G21830.1 |  | Cysteine/Histidine-rich C1 domain family protein |
|  |  | 3,0 | Cleavage | AT2G31865.1 | ARG2 | Poly(ADP-ribose) glycohydrolase 2 |
| miR472-3p | CDP-P  CDP-L | 0,5  0,5  0,5  0,5  1,0  2,0  2,5 | Cleavage  Cleavage  Cleavage  Cleavage  Cleavage  Cleavage  Translation | AT1G12290.1  AT1G12290.2  AT5G43740.1  AT5G43740.2  AT1G51480.1  AT5G63020.1  AT1G62630.1 |  | Disease resistance protein (CC-NBS-LRR class) family |
|  |  | 2,0 | Cleavage | AT1G12210.1 | RFL1 | RPS5-like 1 |
|  |  | 2,0  2,5 | Cleavage  Cleavage | AT1G12280.1  AT4G10780.1 |  | LRR and NB-ARC domains-containing disease resistance protein |
|  |  | 2,5 | Cleavage | AT1G12220.1 | RPS5 | Disease resistance protein (CC-NBS-LRR class) family |
|  |  | 2,5 | Cleavage | AT4G27190.1 |  | NB-ARC domain-containing disease resistance protein |
|  |  | 3,0 | Cleavage | AT5G47260.1 |  | ATP binding;GTP binding;nucleotide binding;nucleoside-triphosphatases |
|  |  | 3,0 | Cleavage | AT3G26840.1 |  | Esterase/lipase/thioesterase family protein |
|  |  | 3,0 | Cleavage | AT1G64780.1 | ATAMT1;2, AMT1;2 | Ammonium transporter 1;2 |
|  |  | 3,5  3,5 | Translation  Translation | AT4G02450.1  AT4G02450.2 |  | HSP20-like chaperones superfamily protein |
|  |  | 3,5  3,5 | Cleavage  Cleavage | AT1G52730.1  AT1G52730.2 |  | Transducin/WD40 repeat-like superfamily protein |
| miR472-5p | CDP-P | 3,0 | Cleavage | AT4G26940.2 |  | Galactosyltransferase family protein |
|  |  | 3,0  3,0 | Cleavage  Cleavage | AT1G42040.1  AT1G42070.1 |  | Transposable element gene |
|  |  | 3,0 | Cleavage | AT4G24340.1 |  | Phosphorylase superfamily protein |
|  |  | 3,0 | Cleavage | AT3G57580.1 |  | F-box and associated interaction domains-containing protein |
|  |  | 3,0 | Cleavage | AT3G57580.1 |  | F-box and associated interaction domains-containing protein |
|  |  | 3,0 | Cleavage | AT3G22270.1 |  | Topoisomerase II-associated protein PAT1 |
|  |  | 3,0 | Cleavage | AT2G29740.1 | UGT71C2 | UDP-glucosyl transferase 71C2 |
|  |  | 3,0 | Cleavage | AT4G34980.1 | SLP2 | Subtilisin-like serine protease 2 |
|  |  | 3,5 | Cleavage | AT4G07410.1  AT4G07410.2 |  | Transducin family protein / WD-40 repeat family protein |
|  |  | 3,5 | Cleavage | AT3G48480.1 |  | Cysteine proteinases superfamily protein |
| miR773a | CDP-L | 2,0 | Cleavage | AT4G05390.1 | ATRFNR1, RFNR1 | Root FNR 1 |
|  |  | 2,0 | Cleavage | AT4G05390.2 | RFNR1 | Root FNR 1 |
|  |  | 2,0  2,5  2,5  3,0 | Cleavage  Cleavage  Cleavage  Translation | AT5G28776.1  AT3G31630.1  AT3G30570.1  AT3G33151.1 |  | Transposable element gene |
|  |  | 2,5 | Cleavage | AT5G51210.1 | OLEO3 | Oleosin3 |
|  |  | 2,5 | Cleavage | AT3G12012.1 | CPuORF20 | Conserved peptide upstream open reading frame 20 |
|  |  | 2,5 | Cleavage | AT1G79950.1 |  | RAD3-like DNA-binding helicase protein |
|  |  | 3,0 | Cleavage | AT4G14140.1 | MET2, DMT02, DMT2, MET02 | DNA methyltransferase 2 |
|  |  | 3,0 | Cleavage | AT4G14920.1 |  | Acyl-CoA N-acyltransferase with RING/FYVE/PHD-type zinc finger protein |
|  |  | 3,0 | Translation | AT5G64270.1 |  | Splicing factor, putative |
|  |  | 3,0 | Cleavage | AT5G57350.1 |  | AHA3, ATAHA3, HA3 \| H(+)-ATPase 3 |
| miR775 | CDP-P  CDP-L | 2,0 | Cleavage | AT1G53290.1 |  | Galactosyltransferase family protein |
|  |  | 3,0 | Cleavage | AT1G01040.2 | DCL1 | Dicer-like 1 |
|  |  | 3,0 | Cleavage | AT1G01040.1 | DCL1, CAF, SUS1, SIN1, ASU1, EMB76, EMB60, ATDCL1 | Dicer-like 1 |
|  |  | 3,0 | Cleavage | AT1G12240.1 | ATBETAFRUCT4, VAC-INV | Glycosyl hydrolases family 32 protein |
|  |  | 3,5  3,5 | Translation  Cleavage | AT1G21245.1  AT3G24190.1 |  | Protein kinase superfamily protein |
|  |  | 3,5 | Translation | AT1G23390.1 |  | Kelch repeat-containing F-box family protein |
|  |  | 3,5 | Cleavage | AT3G14980.1 |  | Acyl-CoA N-acyltransferase with RING/FYVE/PHD-type zinc finger protein |
|  |  | 3,5  3,5 | Translation  Translation | AT1G73960.1  AT1G73960.2 | TAF2 | TBP-associated factor 2 |
|  |  | 3,5 | Cleavage | AT2G32750.1 |  | Exostosin family protein |
|  |  | 3,5 | Cleavage | AT4G21530.1 |  | Transducin/WD40 repeat-like superfamily protein |
| miR823 | CDP-P  CDP-L | 1,5 | Cleavage | AT1G69770.1 | CMT3 | Chromomethylase 3 |
|  |  | 3,0 | Translation | AT1G31885.1 | NIP3 | NOD26-like intrinsic protein 3;1 |
|  |  | 3,0  3,0 | Cleavage  Cleavage | AT1G76140.1  AT1G76140.2 |  | Prolyl oligopeptidase family protein |
|  |  | 3,5 | Cleavage | AT1G05310.1 |  | Pectin lyase-like superfamily protein |
|  |  | 3,5 | Cleavage | AT5G49180.1 |  | Plant invertase/pectin methylesterase inhibitor superfamily |
|  |  | 3,5 | Cleavage | AT2G38680.1 |  | 5'-nucleotidases;magnesium ion binding |
|  |  | 3,5 | Cleavage | AT5G20320.1 | DCL4, ATDCL4 | Dicer-like 4 |
|  |  | 3,5 | Translation | AT1G08060.2 | MOM, MOM1 | ATP-dependent helicase family protein |
|  |  | 3,5  3,5  3,5  3,5 | Cleavage  Cleavage  Cleavage  Cleavage | AT3G26020.1  AT3G26020.1  AT3G26020.1  AT3G26020.1 |  | Protein phosphatase 2A regulatory B subunit family protein |
|  |  | 3,5 | Cleavage | AT1G52920.1 | GCR2, GPCR | G protein coupled receptor |
| miR824-3p | CDP-P  CDP-L | 2,0 | Cleavage | AT3G08870.1 |  | Concanavalin A-like lectin protein kinase family protein |
|  |  | 2,5 | Cleavage | AT4G25370.1 |  | Double Clp-N motif protein |
|  |  | 3,0 | Cleavage | AT5G04640.1 | AGL99 | AGAMOUS-like 99 |
|  |  | 3,0  3,0 | Cleavage  Cleavage | AT5G27300.1  AT5G27300.2 |  | Pentatricopeptide (PPR) repeat-containing protein |
|  |  | 3,0 | Cleavage | AT2G20030.1 | RING | U-box superfamily protein \| |
|  |  | 3,0 | Cleavage | AT3G19050.1 | POK2 | phragmoplast orienting kinesin 2 |
|  |  | 3,0  3,0 | Cleavage  Cleavage | AT4G07806.1  AT4G37705.1 |  | Transposable element gene |
|  |  | 3,5 | Translation | AT3G12460.1 |  | Polynucleotidyl transferase, ribonuclease H-like superfamily protein |
|  |  | 3,5 | Cleavage | AT5G41180.1 |  | Leucine-rich repeat transmembrane protein kinase family protein |
|  |  | 3,5  3,5 | Cleavage  Cleavage | AT3G12080.1  AT3G12080.2 | emb2738 | GTP-binding family protein |
| miR829-5p | CDP-P | 2,0 | Cleavage | AT1G61215.1 | BRD4 | Bromodomain 4 |
|  |  | 2,0 | Cleavage | AT3G06270.1 |  | Protein phosphatase 2C family protein |
|  |  | 2,5 | Cleavage | AT4G20140.1 | GSO1 | Leucine-rich repeat transmembrane protein kinase |
|  |  | 2,5 | Cleavage | AT4G35910.1 |  | Adenine nucleotide alpha hydrolases-like superfamily protein |
|  |  | 2,5 | Cleavage | AT1G14840.1 | ATMAP70-4, MAP70-4 | Microtubule-associated proteins 70-4 |
|  |  | 2,5 | Cleavage | AT1G14840.2 | MAP70-4 | Microtubule-associated proteins 70-4 |
|  |  | 2,5 | Cleavage | AT5G55540.1 | TRN1, LOP1 | Tornado 1 |
|  |  | 3,0 | Cleavage | AT4G18950.1 |  | Integrin-linked protein kinase family |
|  |  | 3,0  3,0  3,0 | Cleavage  Cleavage  Cleavage | AT2G41070.1  AT2G41070.2  AT2G41070.3 | EEL, ATBZIP12, DPBF4 | Basic-leucine zipper (bZIP) transcription factor family protein |
|  |  | 3,0 | Cleavage | AT3G52680.1 |  | F-box/RNI-like/FBD-like domains-containing protein |
| miR831-3p | P-L  CDP-L | 2,0  2,5  2,5 | Cleavage  Cleavage  Cleavage | AT2G15017.1  AT2G15016.1  AT2G15014.1 | MBD3 | Methyl-CpG-binding domain; DNA binding |
|  |  | 2,0  2,0 | Cleavage  Cleavage | AT1G79920.1  AT1G79920.2 | Hsp 70 | Heat shock protein 70 family protein |
|  |  | 2,0  2,0 | Cleavage  Cleavage | AT1G79930.1  AT1G79930.2 | HSP91 | Heat shock protein 91 |
|  |  | 2,0 | Cleavage | AT2G29700.1 | ATPH1, PH1 | Pleckstrin homologue 1 |
|  |  | 2,5  2,5 | Cleavage  Cleavage | AT1G69220.1  AT1G69220.2 | SIK1 | Protein kinase superfamily protein |
|  |  | 2,5 | Cleavage | AT3G11120.1 |  | Ribosomal protein L41 family |
|  |  | 2,5  2,5  2,5 | Translation  Translation  Translation | AT3G17611.1  AT3G17611.2  AT3G17611.3 | TRBL14, RBL14 | RHOMBOID-like protein 14 |
|  |  | 2,5 | Cleavage | AT1G63100.1 |  | GRAS family transcription factor |
|  |  | 2,5 | Cleavage | AT3G57290.1 | EIF3E, TIF3E1, ATEIF3E-1, INT-6, ATINT6, INT6 | Eukaryotic translation initiation factor 3E |
|  |  | 2,5  2,5 | Cleavage  Cleavage | AT2G43440.1  AT2G43445.1 |  | F-box and associated interaction domains-containing protein |
| miR831-5p | P-L | 2,5 | Cleavage | AT1G20980.1 | SPL14, FBR6, SPL1R2, ATSPL14 | Squamosa promoter binding protein-like 14 |
|  |  | 3,0 | Cleavage | AT1G49880.1 | Erv1 | Erv1/Alr family protein |
|  |  | 3,0 | Cleavage | AT1G23400.1 | CAF2, ATCAF2 | RNA-binding CRS1 / YhbY (CRM) domain-containing protein |
|  |  | 3,0 | Cleavage | AT5G51060.1 | RHD2, ATRBOHC, RBOHC | NADPH/respiratory burst oxidase protein D |
|  |  | 3,0 | Cleavage | AT2G18400.1 |  | Ribosomal protein L6 family protein |
|  |  | 3,0 | Cleavage | AT1G35560.1 |  | TCP family transcription factor |
|  |  | 3,0 | Cleavage | AT1G21460.1 | SWEET1, AtSWEET1 | Nodulin MtN3 family protein |
|  |  | 3,0  3,0 | Cleavage  Cleavage | AT5G02560.1  AT5G02560.2 | HTA12 | Histone H2A 12 |
|  |  | 3,0 | Cleavage | AT5G48120.1 |  | ARM repeat superfamily protein |
|  |  | 3,5  3,5 | Translation  Translation | AT5G08560.1  AT5G08560.2 |  | Transducin family protein / WD-40 repeat family protein |
| miR833a-5p | CDP-L | 2,5 | Cleavage | AT2G36890.1 | RAX2, MYB38, ATMYB38, BIT1 | Duplicated homeodomain-like superfamily protein |
|  |  | 2,5 | Cleavage | AT5G52400.1 | CYP715A1 | Cytochrome P450, family 715, subfamily A, polypeptide 1 |
|  |  | 3,0 | Cleavage | AT4G34170.1 |  | Galactose oxidase/kelch repeat superfamily protein |
|  |  | 3,0 | Cleavage | AT1G31050.1 |  | Basic helix-loop-helix (bHLH) DNA-binding superfamily protein |
|  |  | 3,0 | Cleavage | AT1G34480.1 |  | Cysteine/Histidine-rich C1 domain family protein |
|  |  | 3,0 | Cleavage | AT1G37020.1 |  | Cysteine proteinases superfamily protein |
|  |  | 3,0 | Cleavage | AT4G10270.1 |  | Wound-responsive family protein |
|  |  | 3,5  3,5  3,5  3,5 | Cleavage  Cleavage  Cleavage  Cleavage | AT5G62760.1  AT5G62760.2  AT5G62760.3  AT5G62760.4 |  | P-loop containing nucleoside triphosphate hydrolases superfamily protein |
|  |  | 3,5 | Cleavage | AT5G47550.1 |  | Cystatin/monellin superfamily protein |
|  |  | 3,5  3,5  3,5 | Cleavage  Cleavage  Cleavage | AT2G25290.1  AT2G25290.2  AT2G25290.3 | Phox1 | Octicosapeptide/Phox/Bem1p (PB1) domain-containing protein / tetratricopeptide repeat (TPR)-containing protein |
| miR838 | CDP-L | 2,0 | Cleavage | AT5G20110.1 |  | Dynein light chain type 1 family protein |
|  |  | 2,0  2,5 | Translation  Cleavage | AT1G35390.1  AT3G42836.1 |  | Transposable element gene |
|  |  | 2,0  2,0 | Translation  Translation | AT5G22640.1  AT5G22640.2 | emb1211 | MORN (Membrane Occupation and Recognition Nexus) repeat-containing protein |
|  |  | 2,0  2,0 | Cleavage  Cleavage | AT2G45720.1  AT2G45720.2 |  | ARM repeat superfamily protein |
|  |  | 2,0 | Translation | AT1G67230.1 | LINC 1 | Little nuclei1 |
|  |  | 2,0 | Cleavage | AT1G62480.1 |  | Vacuolar calcium-binding protein-related |
|  |  | 2,0 | Cleavage | AT5G02010.1 | ATROPGEF7, ROPGEF7 | RHO guanyl-nucleotide exchange factor 7 |
|  |  | 2,0  2,0 | Cleavage  Cleavage | AT3G26850.1  AT3G26850.2 |  | Histone-lysine N-methyltransferases |
|  |  | 2,5  2,5 | Cleavage  Cleavage | AT1G09730.1  AT1G09730.1 |  | Cysteine proteinases superfamily protein |
|  |  | 2,5  2,5 | Cleavage  Cleavage | AT3G12380.1  AT3G12380.2 | ATARP5, ARP5 | Actin-related protein 5 |
| miR840-5p | CDP-P  CDP-L | 3,0 | Cleavage | AT1G50770.1 |  | Aminotransferase-like, plant mobile domain family protein |
|  |  | 3,5 | Cleavage | AT1G69570.1 |  | Dof-type zinc finger DNA-binding family protein |
|  |  | 3,5 | Cleavage | AT1G64740.1 | TUA1 | Alpha-1 tubulin |
|  |  | 3,5 | Cleavage | AT2G24590.1 | RSZ22a, At-RSZ22a | RNA recognition motif and CCHC-type zinc finger domains containing protein |
|  |  | 3,5 | Cleavage | AT5G35450.1 |  | Disease resistance protein (CC-NBS-LRR class) family |
|  |  | 4,0 | Cleavage | AT3G02250.1 |  | O-fucosyltransferase family protein |
|  |  | 4,0  4,0 | Cleavage  Cleavage | AT1G68550.1  AT1G68550.2 |  | Integrase-type DNA-binding superfamily protein |
|  |  | 4,0 | Cleavage | AT2G03150.1 |  | ATP/GTP-binding protein family |
|  |  | 4,0 | Cleavage | AT1G10580.1 |  | Transducin/WD40 repeat-like superfamily protein |
|  |  | 4,0 | Cleavage | AT5G15280.1 |  | Pentatricopeptide repeat (PPR) superfamily protein |
| miR841a-3p | CDP-P  CDP-L | 2,5  3,0  3,5  3,5  3,5 | Cleavage  Cleavage  Cleavage  Cleavage  Cleavage | AT3G31935.1  AT4G06578.1  AT2G04130.1  AT2G11180.1  AT3G28705.1 |  | Transposable element gene |
|  |  | 3,0 | Cleavage | AT2G27120.1 | POL2B, TIL2 | DNA polymerase epsilon catalytic subunit |
|  |  | 3,5 | Cleavage | AT3G61780.1 | emb1703 | Embryo defective 1703 |
|  |  | 3,5 | Cleavage | AT2G20400.1 |  | Myb-like HTH transcriptional regulator family protein |
|  |  | 3,5 | Cleavage | AT1G08260.1 | EMB142, EMB2284, POL2A, TIL1, EMB529, ABO4, ESD7 | DNA polymerase epsilon catalytic subunit |
|  |  | 3,5  3,5 | Cleavage  Cleavage | AT2G30460.1  AT2G30460.2 |  | Nucleotide/sugar transporter family protein |
|  |  | 3,5 | Cleavage | AT1G35230.1 | AGP5 | Arabinogalactan protein 5 |
|  |  | 3,5 | Cleavage | AT4G04830.1 | ATMSRB5, MSRB5 | Methionine sulfoxide reductase B5 |
|  |  | 3,5  3,5 | Cleavage  Cleavage | ATCG00860.1  ATCG01280.1 | YCF2.1 | Chloroplast Ycf2;ATPase, AAA type, core |
|  |  | 4,0 | Cleavage | AT1G47790.1 |  | F-box and associated interaction domains-containing protein |
| miR841a-5p | CDP-L | 2,0  2,5 | Cleavage  Cleavage | AT4G13570.1 AT4G13570.2 | HTA4 | Histone H2A 4 |
|  |  | 2,5  2,5  2,5  2,5 | Cleavage  Cleavage  Cleavage  Cleavage | AT5G49470.1  AT5G49470.2  AT5G49470.3  AT5G49470.4 |  | PAS domain-containing protein tyrosine kinase family protein |
|  |  | 3,0  3,0  3,0 | Cleavage  Cleavage  Cleavage | AT2G38810.1  AT2G38810.2  AT2G38810.3 | HTA8 | Histone H2A 8 |
|  |  | 3,0  3,0 | Cleavage  Cleavage | AT4G24270.1 AT4G24270.2 | EMB140 | EMBRYO DEFECTIVE 140 |
|  |  | 3,5 | Cleavage | AT3G08970.1 | ATERDJ3A, TMS1 | DNAJ heat shock N-terminal domain-containing protein |
|  |  | 3,5 | Cleavage | AT4G37000.1 | ACD2, ATRCCR | Accelerated cell death 2 (ACD2) |
|  |  | 4,0 | Translation | AT1G64470.1 |  | Ubiquitin-like superfamily protein |
|  |  | 4,0  4,0 | Cleavage  Cleavage | AT5G15410.1  AT5G15410.2 | DND1, ATCNGC2, CNGC2 | Cyclic nucleotide-regulated ion channel family protein |
|  |  | 4,0 | Cleavage | AT3G53300.1 | CYP71B31 | Cytochrome P450, family 71, subfamily B, polypeptide 31 |
|  |  | 4,0  4,0 | Translation  Translation | AT1G62600.1  AT1G62620.1 |  | Flavin-binding monooxygenase family protein |
| miR841b-3p | CDP-P  CDP-L | 2,5  3,0  3,0  3,0 | Cleavage  Cleavage  Cleavage  Cleavage | AT3G28705.1  AT2G04130.1  AT2G11180.1  AT3G31935.1 |  | Transposable element gene |
|  |  | 3,0  3,0 | Cleavage  Cleavage | AT1G61500.1  AT1G61480.1 |  | S-locus lectin protein kinase family protein |
|  |  | 3,5 | Cleavage | AT4G04830.1 | ATMSRB5, MSRB5 | Methionine sulfoxide reductase B5 |
|  |  | 3,5 | Cleavage | AT4G04830.2 | MSRB5 | Methionine sulfoxide reductase B5 |
|  |  | 3,5 | Cleavage | AT1G33110.2 |  | MATE efflux family protein |
|  |  | 3,5  3,5  3,5 | Translation  Translation  Translation | AT3G20270.1  AT3G20270.2  AT3G20270.3 |  | Lipid-binding serum glycoprotein family protein |
|  |  | 3,5  3,5 | Cleavage  Cleavage | AT4G35600.1  AT4G35600.2 | CONNEXIN 32 | Protein kinase superfamily protein |
|  |  | 3,5  3,5  3,5 | Translation  Translation  Translation | AT3G20270.1  AT3G20270.2  AT3G20270.3 |  | Lipid-binding serum glycoprotein family protein |
|  |  | 4,0 | Cleavage | AT1G74770.1 |  | Zinc ion binding |
|  |  | 4,0 | Translation | AT1G12490.1 |  | F-box associated ubiquitination effector family protein |
| miR841b-5p | CDP-P  CDP-L | 1,5 | Cleavage | AT2G38810.1  AT2G38810.2  AT2G38810.3 | HTA8 | Histone H2A 8 |
|  |  | 3,5 | Cleavage | AT4G18060.1 |  | SH3 domain-containing protein |
|  |  | 3,5 | Cleavage | AT4G13570.1 | HTA4 | Histone H2A 4 |
|  |  | 3,5 | Cleavage | AT2G25687.1 |  | Encodes a defensin-like (DEFL) family protein |
|  |  | 4,0 | Cleavage | AT5G54640.1 | HTA1, RAT5, ATHTA1 | Histone superfamily protein |
|  |  | 4,0 | Cleavage | AT1G70940.1 | PIN3, ATPIN3 | Auxin efflux carrier family protein |
|  |  | 4,0 | Cleavage | AT4G05230.1 |  | Ubiquitin-like superfamily protein |
|  |  | 4,0  4,0  4,0 | Cleavage  Cleavage  Cleavage | AT5G49470.1  AT5G49470.2  AT5G49470.3 |  | PAS domain-containing protein tyrosine kinase family protein |
|  |  | 4,0  4,0 | Cleavage  Cleavage | AT5G21040.1  AT5G21040.2 | FBX2 | F-box protein 2 |
|  |  | 4,0  4,0 | Translation  Translation | AT5G44740.1  AT5G44740.2 | POLH | Y-family DNA polymerase H |
| miR858a | CDP-L | 1,0 | Cleavage | AT2G26950.1 | AtMYB104, MYB104 | Myb domain protein 104 |
|  |  | 1,0 | Cleavage | AT1G06180.1 | ATMYB13, ATMYBLFGN, MYB13 | Myb domain protein 13 |
|  |  | 1,5 | Cleavage | AT5G35550.1 | TT2, ATMYB123, MYB123, ATTT2 | Duplicated homeodomain-like superfamily protein |
|  |  | 2,5 | Cleavage | AT1G79650.2 | RAD23, RAD23B | Rad23 UV excision repair protein family |
|  |  | 3,0 | Cleavage | AT1G17760.1 | CSTF77, ATCSTF77 | Tetratricopeptide repeat (TPR)-like superfamily protein |
|  |  | 3,0 | Cleavage | AT2G26420.1 | PIP5K3 | 1-phosphatidylinositol-4-phosphate 5-kinase 3 |
|  |  | 3,0  3,0 | Cleavage  Cleavage | AT3G57020.1  AT3G57020.2 |  | Calcium-dependent phosphotriesterase superfamily protein |
|  |  | 3,0  3,0 | Cleavage  Cleavage | AT4G34860.1  AT4G34860.2 |  | Plant neutral invertase family protein |
|  |  | 3,5  3,5 | Cleavage  Cleavage | AT5G10100.1  AT5G10100.2 | TPPI | Haloacid dehalogenase-like hydrolase (HAD) superfamily protein \| |
|  |  | 3,5 | Cleavage | AT4G21510.1 |  | F-box family protein |
| miR858b | CDP-L | 1,5 | Cleavage | AT5G35550.1 | TT2, ATMYB123, MYB123, ATTT2 | Duplicated homeodomain-like superfamily protein |
|  |  | 1,5 | Cleavage | AT3G08500.1 | MYB83, AtMYB83 | Myb domain protein 83 |
|  |  | 2,0 | Cleavage | AT2G26950.1 | AtMYB104, MYB104 | Myb domain protein 104 |
|  |  | 2,0 | Cleavage | AT1G06180.1 | ATMYB13, ATMYBLFGN, MYB13 | Myb domain protein 13 |
|  |  | 3,0 | Cleavage | AT1G17760.1 | CSTF77, ATCSTF77 | Tetratricopeptide repeat (TPR)-like superfamily protein |
|  |  | 3,0  3,5 | Cleavage  Cleavage | AT2G09910.1  AT3G42727.1 |  | Transposable element gene |
|  |  | 3,5 | Cleavage | AT3G27170.1 | CLC-B, ATCLC-B | Chloride channel B |
|  |  | 3,5 | Translation | AT3G20630.1 | UBP14, TTN6, ATUBP14, PER1 | Ubiquitin-specific protease 14 |
|  |  | 3,5 | Cleavage | AT4G02990.1 |  | Mitochondrial transcription termination factor family protein |
|  |  | 3,5 | Cleavage | AT2G26420.1 | PIP5K3 | 1-phosphatidylinositol-4-phosphate 5-kinase 3 |
|  |  | 3,5 | Translation | AT1G79930.2 | HSP91 | Heat shock protein 91 |
| miR859 | CDP-P  CDP-L | 0,5  0,5  1,0  1,0  1,0  1,0 | Cleavage  Cleavage  Cleavage  Cleavage  Cleavage  Cleavage | AT3G17265.1  AT5G36200.1  AT3G16820.1  AT3G16880.1  AT3G22350.1  AT3G17570.1 |  | F-box and associated interaction domains-containing protein |
|  |  | 1,0  1,0 | Cleavage  Cleavage | AT3G22710.1  AT3G49510.1 |  | F-box family protein |
|  |  | 1,5 | Cleavage | AT3G14030.1 |  | F-box associated ubiquitination effector family protein |
|  |  | 2,5 | Cleavage | AT1G65990.1 |  | type 2 peroxiredoxin-related / thiol specific antioxidant / mal allergen family protein |
|  |  | 2,5 | Cleavage | AT1G67830.1 | ATFXG1, FXG1 | alpha-fucosidase 1 |
|  |  | 2,5 | Cleavage | AT3G01750.1 |  | Ankyrin repeat family protein |
|  |  | 3,0 | Cleavage | AT3G26580.1 | AT3G26580.1 | Tetratricopeptide repeat (TPR)-like superfamily protein |
|  |  | 3,0  3,0 | Cleavage  Cleavage | AT2G07360.1  AT2G07360.2 |  | SH3 domain-containing protein |
|  |  | 3,0 | Cleavage | AT1G60610.1 |  | SBP (S-ribonuclease binding protein) family protein |
|  |  | 3,5  3,5 | Cleavage  Cleavage | AT3G18980.1  AT3G18980.2 | ETP1 | EIN2 targeting protein1 |
|  |  | 3,5 | Cleavage | AT4G14030.1 | SBP1 | Selenium-binding protein 1 |
|  |  | 3,5 | Cleavage | AT1G29940.1 | NRPA2 | Nuclear RNA polymerase A2 |
| miR861-5p | CDP-P | 2,5  2,5 | Cleavage  Cleavage | AT1G43145.1  AT1G43145.2 |  | S-adenosyl-L-methionine-dependent methyltransferases superfamily protein |
|  |  | 3,5 | Cleavage | AT5G35940.1 |  | Mannose-binding lectin superfamily protein |
|  |  | 3,5 | Cleavage | AT5G45116.1 |  | Transposable element gene |
|  |  | 3,5 | Cleavage | AT3G46700.1 |  | UDP-Glycosyltransferase superfamily protein |
|  |  | 3,5  3,5 | Cleavage  Cleavage | AT3G09560.2  AT3G09560.3 |  | Lipin family protein |
|  |  | 3,5 | Cleavage | AT3G09560.1 | ATPAH1, PAH1 | Lipin family protein |
|  |  | 3,5  3,5  3,5 | Cleavage  Cleavage  Cleavage | AT5G51460.1  AT5G51460.2  AT5G51460.3 | ATTPPA | Haloacid dehalogenase-like hydrolase (HAD) superfamily protein |
|  |  | 3,5 | Cleavage | AT2G39260.1 |  | Binding;RNA binding |
|  |  | 4,0 | Cleavage | AT3G24495.1 | MSH7, MSH6-2, ATMSH7 | MUTS homolog 7 |
|  |  | 4,0  4,0 | Cleavage  Cleavage | AT4G35785.1  AT4G35785.2 |  | RNA-binding (RRM/RBD/RNP motifs) family protein |
| miR863-3p | CDP-P  CDP-L | 2,5  2,5 | Cleavage  Cleavage | AT2G29980.1  AT2G29980.2 | FAD3 | Fatty acid desaturase 3 |
|  |  | 2,5  2,5 | Cleavage  Cleavage | AT5G61570.1  AT5G61570.2 |  | Protein kinase superfamily protein |
|  |  | 3,0 | Cleavage | AT2G27100.1 | SE | C2H2 zinc-finger protein SERRATE (SE) |
|  |  | 3,0 | Cleavage | AT5G14750.1 | WER, ATMYB66, WER1, MYB66 | Myb domain protein 66 |
|  |  | 3,0  3,0  3,5 | Cleavage  Cleavage  Cleavage | AT5G29043.1  AT5G32514.1  AT4G37705.1 |  | Transposable element gene |
|  |  | 3,0 | Cleavage | AT4G22820.1 |  | A20/AN1-like zinc finger family protein |
|  |  | 3,0 | Cleavage | AT5G63630.1 |  | P-loop containing nucleoside triphosphate hydrolases superfamily protein |
|  |  | 3,0 | Cleavage | AT4G01390.1 |  | TRAF-like family protein |
|  |  | 3,5 | Translation | AT1G23410.1 |  | Ribosomal protein S27a / Ubiquitin family protein |
| miR865-5p | CDP-L | 2,5 | Translation | AT5G07140.1 |  | Protein kinase superfamily protein |
|  |  | 2,5 | Cleavage | AT3G29572.1 |  | Transposable element gene |
|  |  | 2,5 | Cleavage | AT3G51895.1 | SULTR3;1, AST12 | Sulfate transporter 3;1 |
|  |  | 2,5 | Cleavage | ATCG01010.1 | NDHF | NADH-Ubiquinone oxidoreductase (complex I), chain 5 protein |
|  |  | 3,0 | Cleavage | AT5G42240.1 | scpl42 | Serine carboxypeptidase-like 42 |
|  |  | 3,0  3,0 | Cleavage  Cleavage | AT1G51805.1  AT1G51805.2 |  | Leucine-rich repeat protein kinase family protein |
|  |  | 3,0 | Cleavage | AT5G61500.1 | ATATG3, ATG3 | Autophagy 3 (APG3) |
|  |  | 3,0 | Cleavage | AT1G61050.1 |  | Alpha 1,4-glycosyltransferase family protein |
|  |  | 3,0 | Cleavage | AT1G10870.1 | AGD4 | ARF-GAP domain 4 |
|  |  | 3,0 | Cleavage | AT2G45510.1 | CYP704A2 | Cytochrome P450, family 704, subfamily A, polypeptide 2 |
| miR866-3p | P-L  CDP-P  CDP-L | 1,5  2,5 | Cleavage  Cleavage | AT2G22070.1  AT1G20230.1 |  | Pentatricopeptide (PPR) repeat-containing protein |
|  |  | 2,0 | Cleavage | AT4G21400.1 | CRK28 | Cysteine-rich RLK (RECEPTOR-like protein kinase) 28 |
|  |  | 2,5  2,5  2,5  2,5  2,5 | Cleavage  Cleavage  Cleavage  Cleavage  Cleavage | AT3G05380.1  AT3G05380.2  AT3G05380.3  AT3G05380.4  AT3G05380.5 | ALY2, ATALY2 | DIRP ;Myb-like DNA-binding domain |
|  |  | 2,5  2,5  2,5 | Cleavage  Cleavage  Cleavage | AT2G41540.1  AT2G41540.2  AT2G41540.3 | GPDHC1 | 6-phosphogluconate dehydrogenase family protein |
|  |  | 3,0 | Cleavage | AT3G57730.1 |  | Protein kinase superfamily protein |
|  |  | 3,0 | Cleavage | AT5G64640.1 |  | Plant invertase/pectin methylesterase inhibitor superfamily |
|  |  | 3,0 | Cleavage | AT4G20110.1 | VSR7, VSR3;1, BP80-3;1 | VACUOLAR SORTING RECEPTOR 7 |
|  |  | 3,0 | Cleavage | AT4G20110.2 | VSR7 | VACUOLAR SORTING RECEPTOR 7 |
|  |  | 3,0 | Cleavage | AT5G54800.1 | GPT1, ATGPT1 | Glucose 6-phosphate/phosphate |
|  |  | 3,0 | Cleavage | AT1G48310.1 | CHR18, CHA18 | Chromatin remodeling factor18 |
| miR866-5p | CDP-P  CDP-L | 1,5 | Cleavage | AT5G06510.2 | NF-YA10 | Nuclear factor Y, subunit A10 |
|  |  | 1,5 | Cleavage | AT3G13820.1 |  | F-box and associated interaction domains-containing protein |
|  |  | 2,5  2,5 | Cleavage  Cleavage | AT4G23340.1  AT4G23340.2 |  | 2-oxoglutarate (2OG) and Fe(II)-dependent oxygenase superfamily protein |
|  |  | 2,5 | Cleavage | AT1G07620.1 |  | GTP-binding protein Obg/CgtA |
|  |  | 3,0 | Cleavage | AT4G33430.2 | BAK1 | BRI1-associated receptor kinase |
|  |  | 3,0 | Cleavage | AT1G12970.1 | PIRL3 | Plant intracellular ras group-related LRR 3 |
|  |  | 3,0 | Cleavage | AT1G52310.1 |  | Protein kinase family protein / C-type lectin domain-containing protein |
|  |  | 3,0 | Cleavage | AT1G54720.1 |  | Early-responsive to dehydration protein-related / ERD protein-related |
|  |  | 3,0 | Cleavage | AT1G01580.1 | FRO2, FRD1, ATFRO2 | Ferric reduction oxidase 2 |
|  |  | 3,0 | Cleavage | AT2G02520.1 |  | RNA-directed DNA polymerase (reverse transcriptase)-related family protein |
| miR870-3p | CDP-P | 2,0  2,5  2,5  3,0 | Cleavage  Cleavage  Cleavage  Cleavage | AT1G35970.1  AT5G34839.1  AT1G35960.1  AT1G40091.1 |  | Transposable element gene |
|  |  | 2,5 | Translation | AT4G02530.1 |  | Chloroplast thylakoid lumen protein |
|  |  | 3,0 | Cleavage | AT5G41290.1 |  | Receptor-like protein kinase-related family protein |
|  |  | 3,0 | Cleavage | AT1G16490.1 |  | ATMYB58, MYB58 \| myb domain protein 58 |
|  |  | 3,0  3,0  3,0 | Cleavage  Cleavage  Cleavage | AT5G46610.1  AT5G46610.2  AT5G46610.3 |  | Aluminium activated malate transporter family protein |
|  |  | 3,0 | Cleavage | AT5G40490.1 |  | RNA-binding (RRM/RBD/RNP motifs) family protein |
|  |  | 3,0 | Cleavage | AT2G13720.1 |  | DNA topoisomerase (ATP-hydrolyzing)s;ATP binding;DNA binding |
|  |  | 3,0 | Cleavage | AT5G22355.1 |  | Cysteine/Histidine-rich C1 domain family protein |
|  |  | 3,0 | Cleavage | AT1G48540.2 |  | Outer arm dynein light chain 1 protein |
|  |  | 3,0 | Cleavage | AT3G57740.1 |  | Protein kinase superfamily protein |
| miR2111a-3p | CDP-L | 3,5 | Translation | AT5G10460.1 |  | Haloacid dehalogenase-like hydrolase (HAD) superfamily protein |
|  |  | 3,5 | Cleavage | AT1G16780.1 | VHP2;2, AtVHP2;2 | Inorganic H pyrophosphatase family protein |
|  |  | 3,5 | Cleavage | AT5G19920.1 |  | Transducin/WD40 repeat-like superfamily protein |
|  |  | 3,5  3,5 | Cleavage  Cleavage | AT2G14740.1  AT2G14740.1 | ATVSR3, VSR3, VSR2;2, BP80-2;2 | Vaculolar sorting receptor 3 |
|  |  | 3,5  3,5 | Cleavage  Cleavage | AT4G19960.1  AT4G19960.2 | KUP9 | K+ uptake permease 9 |
|  |  | 3,5 | Cleavage | AT2G07500.1 |  | Transposable element gene |
|  |  | 4,0 | Cleavage | AT1G05470.1 | CVP2 | DNAse I-like superfamily protein |
|  |  | 4,0 | Translation | AT5G47800.1 |  | Phototropic-responsive NPH3 family protein |
|  |  | 4,0 | Cleavage | AT3G52080.1 | chx28 | Cation/hydrogen exchanger 28 |
|  |  | 4,0 | Cleavage | AT5G42970.1 | COP8, FUS4, EMB134, COP14, CSN4, FUS8, ATS4 | Proteasome component (PCI) domain protein |
| miR2111a-5p  miR2111b-5p | CDP-L  CDP-L | 1,0 | Cleavage | AT3G27150.1 |  | Galactose oxidase/kelch repeat superfamily protein |
|  |  | 2,0  2,0  2,0 | Translation  Translation  Translation | AT1G07010.1  AT1G07010.2  AT1G07010.3 |  | Calcineurin-like metallo-phosphoesterase superfamily protein |
|  |  | 2,5 | Cleavage | AT5G41300.1 |  | Receptor-like protein kinase-related family protein |
|  |  | 3,0 | Translation | AT5G52010.1 |  | C2H2-like zinc finger protein |
|  |  | 3,0 | Cleavage | AT3G30695.1 |  | Transposable element gene |
|  |  | 3,0 | Cleavage | AT5G58590.1 | RANBP1 | RAN binding protein 1 |
|  |  | 3,5 | Translation | AT2G19590.1 | ACO1, ATACO1 | ACC oxidase 1 |
|  |  | 3,5  3,5 | Cleavage  Cleavage | AT2G36870.1  AT2G36870.2 | XTH32 | Xyloglucan endotransglucosylase/hydrolase 32 |
|  |  | 3,5  3,5 | Cleavage  Cleavage | AT1G67840.1  AT1G67840.2 | CSK | Chloroplast sensor kinase |
|  |  | 3,5 | Translation | AT1G10620.1 |  | Protein kinase superfamily protein |
| miR3434-3p | CDP-P  CDP-L | 2,5 | Cleavage | AT1G25320.1 |  | Leucine-rich repeat protein kinase family protein |
|  |  | 3,0 | Cleavage | AT3G26840.1 |  | Esterase/lipase/thioesterase family protein |
|  |  | 3,0  3,0  3,0  3,0 | Cleavage  Cleavage  Cleavage  Cleavage | AT3G14860.1  AT3G14860.2  AT1G70280.1  AT1G70280.2 |  | NHL domain-containing protein |
|  |  | 3,0  3,5 | Cleavage  Cleavage | AT4G04280.1  AT3G42431.1 |  | Transposable element gene |
|  |  | 3,5 | Cleavage | AT1G71060.1 |  | Tetratricopeptide repeat (TPR)-like superfamily protein |
|  |  | 3,5 | Cleavage | AT4G01220.2 |  | Nucleotide-diphospho-sugar Transferase family protein |
|  |  | 3,5 | Cleavage | AT1G02620.1 |  | Ras-related small GTP-binding family protein |
|  |  | 3,5 | Cleavage | AT2G26200.1 |  | S-adenosyl-L-methionine-dependent methyltransferases superfamily protein |
|  |  | 3,5 | Cleavage | AT4G29130.1 | ATHXK1, GIN2, HXK1 | Hexokinase 1 |
|  |  | 3,5 | Cleavage | AT5G03280.1 | EIN2, PIR2, CKR1, ERA3, ORE3, ORE2, ATEIN2 | NRAMP metal ion transporter family protein |
| miR3933 | CDP-P | 2,5  2,5 | Cleavage  Cleavage | AT4G22235.1  AT4G22235.2 |  | Arabidopsis defensin-like protein |
|  |  | 2,5 | Cleavage | AT1G77330.1 |  | 2-oxoglutarate (2OG) and Fe(II)-dependent oxygenase superfamily protein |
|  |  | 2,5 | Cleavage | AT1G28335.1 | LCR31 | Low-molecular-weight cysteine-rich 31 |
|  |  | 3,0 | Cleavage | AT2G30580.1 | DRIP2 | DREB2A-interacting protein 2 |
|  |  | 3,0 | Cleavage | AT2G43930.1 |  | Protein kinase superfamily protein |
|  |  | 3,0 | Cleavage | AT4G16580.1 |  | Protein phosphatase 2C family protein |
|  |  | 3,0  3,0 | Cleavage  Cleavage | AT3G03100.1  AT3G03100.2 |  | NADH:ubiquinone oxidoreductase, 17.2kDa subunit |
|  |  | 3,0 | Cleavage | AT3G53080.1 |  | D-galactoside/L-rhamnose binding SUEL lectin protein |
|  |  | 3,0 | Cleavage | AT5G27345.1 |  | Transposable element gene |
|  |  | 3,0 | Cleavage | AT3G60100.1 | CSY5 | Citrate synthase 5 |
| miR4245 | CDP-L | 1,5 | Cleavage | AT1G57620.1 |  | emp24/gp25L/p24 family/GOLD family protein |
|  |  | 3,0  3,0  3,5 | Cleavage  Cleavage  Cleavage | AT1G41910.1  AT2G10000.1  AT3G29766.1 |  | Transposable element gene |
|  |  | 3,0 | Cleavage | AT5G44870.1 | LAZ5 | Disease resistance protein (TIR-NBS-LRR class) family |
|  |  | 3,0  3,0 | Cleavage  Cleavage | AT3G58640.1  AT3G58640.2 |  | Mitogen activated protein kinase kinase kinase-related |
|  |  | 3,5 | Translation | AT5G10820.1 |  | Major facilitator superfamily protein |
|  |  | 3,5 | Translation | AT5G11020.1 |  | Protein kinase superfamily protein |
|  |  | 3,5 | Cleavage | AT1G01230.1 |  | ORMDL family protein |
|  |  | 3,5  3,5 | Cleavage  Cleavage | AT3G29763.1  AT3G29765.1 |  | General transcription factor 2-related zinc finger protein |
|  |  | 3,5  3,5 | Cleavage  Cleavage | AT4G13020.2  AT4G13020.3 | MHK | Protein kinase superfamily protein |
|  |  | 3,5 | Cleavage | AT1G35860.1 | TOC75-I | Translocon outer membrane complex 75-I |
|  |  | 3,5 | Translation | AT2G32310.1 |  | CCT motif family protein |
| miR5014a-5p | CDP-L | 2,5  2,5 | Cleavage  Cleavage | AT1G77480.1  AT1G77480.2 |  | Eukaryotic aspartyl protease family protein |
|  |  | 2,5 | Cleavage | AT5G24470.1 | APRR5, PRR5 | Pseudo-response regulator 5 |
|  |  | 3,0 | Cleavage | AT2G18730.1 | ATDGK3, DGK3 | Diacylglycerol kinase 3 |
|  |  | 3,0 | Cleavage | AT5G55910.1 | D6PK | D6 protein kinase |
|  |  | 3,5 | Cleavage | AT1G22040.1 |  | Galactose oxidase/kelch repeat superfamily protein |
|  |  | 3,5 | Cleavage | AT1G55930.1 |  | CBS domain-containing protein / transporter associated domain-containing protein |
|  |  | 3,5 | Cleavage | AT1G56500.1 |  | Haloacid dehalogenase-like hydrolase family protein |
|  |  | 3,5  3,5 | Cleavage  Cleavage | AT3G15180.1  AT3G15180.2 |  | ARM repeat superfamily protein |
|  |  | 4,0  4,0 | Cleavage  Cleavage | AT2G44100.1  AT2G44100.2 | ATGDI1, AT-GDI1, GDI1 | Guanosine nucleotide diphosphate dissociation inhibitor 1 |
|  |  | 4,0 | Cleavage | AT3G32980.1 |  | ER lumen protein retaining receptor family protein |
| miR5020b | CDP-P  CDP-L | 2,0 | Cleavage | AT2G26882.1 |  | Other RNA |
|  |  | 2,0  2,0 | Cleavage  Cleavage | AT1G60430.1  AT1G60430.2 | ARPC3 | Actin-related protein C |
|  |  | 3,0 | Cleavage | AT4G19191.1 |  | Tetratricopeptide repeat (TPR)-like superfamily protein |
|  |  | 3,0 | Cleavage | AT2G07150.1 |  | Transposable element gene |
|  |  | 3,5 | Cleavage | AT5G51450.2 |  | RPM1 interacting protein 3 |
|  |  | 3,5 | Cleavage | AT1G33350.1 |  | Pentatricopeptide repeat (PPR) superfamily protein |
|  |  | 3,5 | Cleavage | AT1G73440.1 |  | Calmodulin-related |
|  |  | 3,5  3,5 | Cleavage  Cleavage | AT5G52230.1  AT5G52230.2 |  | Methyl-CPG-binding domain protein 13 |
|  |  | 3,5 | Cleavage | AT3G13680.1 |  | F-box and associated interaction domains-containing protein |
|  |  | 3,5  3,5 | Cleavage  Cleavage | AT5G13000.1  AT5G13000.2 | ATGSL12, gsl12 | Glucan synthase-like 12 |
| miR5026 | CDP-P  CDP-L | 3,5 | Translation | AT2G46660.1 | CYP78A6 | Cytochrome P450, family 78, subfamily A, polypeptide 6 |
|  |  | 3,5 | Cleavage | AT2G37870.1 |  | Bifunctional inhibitor/lipid-transfer protein/seed storage 2S albumin superfamily protein |
|  |  | 3,5  4,0 | Cleavage  Cleavage | AT3G32385.1  AT2G10130.1 |  | Transposable element gene |
|  |  | 3,5 | Cleavage | AT1G70230.1 | TBL27 | TRICHOME BIREFRINGENCE-LIKE 27 |
|  |  | 4,0 | Cleavage | AT1G33390.1 | ATFAS4, FAS4 | RNA helicase family protein |
|  |  | 4,0 | Cleavage | AT1G72030.1 |  | Acyl-CoA N-acyltransferases (NAT) superfamily protein |
|  |  | 4,0 | Cleavage | AT5G02960.1 |  | Ribosomal protein S12/S23 family protein |
|  |  | 4,0 | Translation | AT1G10585.1 |  | Basic helix-loop-helix (bHLH) DNA-binding superfamily protein |
|  |  | 4,0 | Cleavage | AT3G02320.1 |  | N2,N2-dimethylguanosine tRNA methyltransferase |
|  |  | 4,0 | Cleavage | AT1G21590.1 |  | Protein kinase protein with adenine nucleotide alpha hydrolases-like domain |
| miR5028 | CDP-P  CDP-L | 2,5 | Cleavage | AT3G10010.1 | DML2 | Demeter-like 2 |
|  |  | 2,5  2,5 | Cleavage  Cleavage | AT1G68540.1  AT1G68540.2 |  | NAD(P)-binding Rossmann-fold superfamily protein |
|  |  | 2,5  2,5 | Cleavage  Cleavage | AT1G56280.1  AT1G56280.2 | -  ATDI19, DI19 | Drought-induced 19 |
|  |  | 2,5 | Cleavage | AT1G69740.1 | HEMB1 | Aldolase superfamily protein |
|  |  | 3,0 | Cleavage | AT1G50740.1 |  | Transmembrane proteins 14C |
|  |  | 3,5  3,5 | Cleavage  Cleavage | AT2G21300.1  AT2G21300.2 |  | ATP binding microtubule motor family protein |
|  |  | 3,5  3,5 | Cleavage  Cleavage | AT5G61240.1  AT5G61240.2 |  | Leucine-rich repeat (LRR) family protein |
|  |  | 3,5 | Cleavage | AT1G13200.1 |  | F-box and associated interaction domains-containing protein |
|  |  | 3,5 | Cleavage | AT1G53140.1 | DRP5A | Dynamin related protein 5A |
|  |  | 3,5 | Cleavage | AT5G36005.1 |  | Transposable element gene |
| miR5635c | CDP-P | 3,0 | Cleavage | AT3G23430.1 | PHO1, ATPHO1 | Phosphate 1 |
|  |  | 3,0 | Cleavage | AT1G05020.1 |  | ENTH/ANTH/VHS superfamily protein |
|  |  | 3,0 | Cleavage | AT1G18940.1 |  | Nodulin-like / Major Facilitator Superfamily protein |
|  |  | 3,5  3,5  3,5 | Cleavage  Cleavage  Cleavage | AT2G06220.1  AT2G07160.1  AT5G33382.1 |  | Transposable element gen |
|  |  | 3,5 | Cleavage | AT5G07170.1 |  | Cell cycle regulated microtubule associated protein |
|  |  | 3,5 | Cleavage | AT2G04190.1 |  | TRAF-like family protein |
|  |  | 4,0  4,0 | Cleavage  Cleavage | AT1G63140.1  AT1G63140.2 |  | O-methyltransferase family protein |
|  |  | 4,0 | Cleavage | AT3G11340.1 |  | UDP-Glycosyltransferase superfamily protein |
|  |  | 4,0 | Cleavage | AT2G38025.1 |  | Cysteine proteinases superfamily protein |
|  |  | 4,0 | Cleavage | AT2G41620.1 |  | Nucleoporin interacting component (Nup93/Nic96-like) family protein |
| miR5653 | CDP-P  CDP-L | 2,0 | Cleavage | AT1G27680.1 | APL2 | ADPGLC-PPase large subunit |
|  |  | 2,0 | Cleavage | AT2G40220.1 | ABI4, SUN6, SIS5, ISI3, GIN6, SAN5, ATABI4 | Integrase-type DNA-binding superfamily protein |
|  |  | 2,0 | Cleavage | AT1G30340.1 |  | Transposable element gene |
|  |  | 2,5 | Translation | AT4G18460.1 |  | D-Tyr-tRNA(Tyr) deacylase family protein |
|  |  | 2,5 | Cleavage | AT3G17770.1 |  | Dihydroxyacetone kinase |
|  |  | 3,0 | Cleavage | AT2G35800.1 |  | Mitochondrial substrate carrier family protein |
|  |  | 3,5 | Cleavage | AT1G60230.1 |  | Radical SAM superfamily protein |
|  |  | 3,5  3,5  3,5 | Cleavage  Cleavage  Cleavage | AT5G43270.1  AT5G43270.2  AT5G43270.3 | PL2 | Squamosa promoter binding protein-like 2 |
|  |  | 3,5 | Cleavage | AT3G60630.1 | HAM2, ATHAM2, LOM2 | GRAS family transcription factor |
|  |  | 3,5 | Cleavage | AT4G30650.1 |  | Low temperature and salt responsive protein family |
| miR5659 | CDP-L | 2,5 | Cleavage | AT1G33750.1 |  | Terpenoid cyclases/Protein prenyltransferases superfamily protein |
|  |  | 2,5 | Cleavage | AT3G13380.1 | BRL3 | BRI1-like 3 |
|  |  | 2,5  2,5 | Cleavage  Cleavage | AT5G28430.1  AT5G32520.1 |  | Transposable element gene |
|  |  | 3,0 | Cleavage | AT5G50300.1 | AZG2, ATAZG2 | Xanthine/uracil permease family protein |
|  |  | 3,0 | Cleavage | AT2G20580.1 | RPN1A, ATRPN1A | 26S proteasome regulatory subunit S2 1A |
|  |  | 3,0  3,0 | Cleavage  Cleavage | AT1G48210.2  AT1G48210.1 |  | Protein kinase superfamily protein |
|  |  | 3,5 | Cleavage | AT5G43580.1 |  | Serine protease inhibitor, potato inhibitor I-type family protein |
|  |  | 3,5 | Translation | AT5G63030.1 |  | Thioredoxin superfamily protein |
|  |  | 3,5 | Translation | AT4G39500.1 | CYP96A11 | Cytochrome P450, family 96, subfamily A, polypeptide 11 |
|  |  | 3,5 | Cleavage | AT2G25440.1 | AtRLP20, RLP20 | Receptor like protein 20 |
| miR5996 | CDP-P  CDP-L | 2,5 | Cleavage | AT5G41300.1 |  | Receptor-like protein kinase-related family protein |
|  |  | 3,0 | Cleavage | AT5G58590.1 | RANBP1 | RAN binding protein 1 |
|  |  | 3,0  3,0 | Cleavage  Cleavage | AT4G00320.1  AT1G58310.1 |  | F-box/RNI-like superfamily protein |
|  |  | 3,0 | Cleavage | AT3G59230.1 |  | RNI-like superfamily protein |
|  |  | 3,0 | Cleavage | AT3G51420.1 | SSL4, ATSSL4 | Strictosidine synthase-like 4 |
|  |  | 3,0 | Cleavage | AT5G66880.1 | SNRK2-3, SNRK2.3, SRK2I | Sucrose nonfermenting 1(SNF1)-related protein kinase 2.3 |
|  |  | 3,5 | Cleavage | AT2G43940.1 | ATHOL3, HOL3 | S-adenosyl-L-methionine-dependent methyltransferases superfamily protein |
|  |  | 3,5 | Translation | AT3G51480.1 | ATGLR3.6, GLR3.6 | Glutamate receptor 3.6 |
|  |  | 3,5 | Cleavage | AT5G15870.1 |  | Glycosyl hydrolase family 81 protein |
|  |  | 3,5  3,5 | Cleavage  Cleavage | AT1G65120.1  AT1G65120.2 |  | Ubiquitin carboxyl-terminal hydrolase-related protein |
| miR8166 | CDP-P  CDP-L | 2,5 | Cleavage | AT1G29470.1 |  | S-adenosyl-L-methionine-dependent methyltransferases superfamily protein |
|  |  | 2,5 | Cleavage | AT3G47380.1 |  | Plant invertase/pectin methylesterase inhibitor superfamily protein |
|  |  | 2,5  2,5  2,5 | Cleavage  Cleavage  Cleavage | AT4G23210.1  AT4G23210.2  AT4G23210.3 | CRK13 | Cysteine-rich RLK (RECEPTOR-like protein kinase) 13 |
|  |  | 3,0 | Cleavage | AT1G67580.1 |  | Protein kinase superfamily protein |
|  |  | 3.0  3,0 | Cleavage  Cleavage | AT2G05120.2  AT2G05120.1 |  | Nucleoporin, Nup133/Nup155-like |
|  |  | 3,0 | Cleavage | AT4G33840.1 |  | Glycosyl hydrolase family 10 protein |
|  |  | 3,0 | Cleavage | AT5G52670.1 |  | Copper transport protein family |
|  |  | 3,0 | Cleavage | AT1G32090.1 |  | Early-responsive to dehydration stress protein (ERD4) |
|  |  | 3,0 | Cleavage | AT5G44370.1 | PHT4;6 | Phosphate transporter 4;6 |
|  |  | 3,0 | Cleavage | AT4G02280.1 | SUS3, ATSUS3 | Sucrose synthase 3 |
| miR8168 | CDP-P  CDP-L | 3,0 | Cleavage | AT4G16440.1 |  | Ferredoxin hydrogenases |
|  |  | 3,0 | Cleavage | AT4G34180.1 |  | Cyclase family protein |
|  |  | 3,0 | Cleavage | AT4G00220.1 | LBD30, JLO \| | Lateral organ boundaries (LOB) domain family protein |
|  |  | 3,0 | Translation | AT2G32370.1 | HDG3 | Homeodomain GLABROUS 3 |
|  |  | 3,5 | Cleavage | AT3G10860.1 |  | Cytochrome b-c1 complex, subunit 8 protein |
|  |  | 3,5 | Cleavage | AT4G10120.1 | ATSPS4F | Sucrose-phosphate synthase family protein |
|  |  | 3,5 | Cleavage | AT2G28350.1 | ARF10 | Auxin response factor 10 |
|  |  | 3,5  3,5 | Cleavage  Cleavage | AT1G19230.2  AT1G19230.1 |  | Riboflavin synthase-like superfamily protein |
|  |  | 2,0  2,0 | Cleavage  Cleavage | AT1G09730.1  AT1G09730.2 |  | Cysteine proteinases superfamily protein |
|  |  | 2,5 | Cleavage | AT3G12380.2 | ARP5 | Actin-related protein 5 |
| miR8169 | CDP-P  CDP-L | 2,5 | Cleavage | AT5G23530.1 | AtCXE18, CXE18 | Carboxyesterase 18 |
|  |  | 2,5  3,0  3,0 | Cleavage  Cleavage  Cleavage | AT2G10540.1  AT4G06528.1  AT2G04490.1 |  | Transposable element gene |
|  |  | 3,0 | Cleavage | AT4G21520.1 |  | Transducin/WD40 repeat-like superfamily protein |
|  |  | 3,0  3,0  3,0 | Cleavage  Cleavage  Cleavage | AT2G31810.3  AT2G31810.2  AT2G31810.1 |  | ACT domain-containing small subunit of acetolactate synthase protein |
|  |  | 3,0  3,0  3,0 | Cleavage  Cleavage  Cleavage | AT4G14103.1  AT4G14103.2 AT3G59170.1 |  | F-box/RNI-like superfamily protein |
|  |  | 3,5  3,5 | Cleavage  Cleavage | AT1G68580.1  AT1G68580.2 |  | Agenet domain-containing protein / bromo-adjacent homology (BAH) domain-containing protein |
|  |  | 3,5 | Cleavage | AT1G51990.1 |  | O-methyltransferase family protein |
|  |  | 3,5 | Cleavage | AT3G12780.1 | PGK1 | Phosphoglycerate kinase 1 |
|  |  | 3,5 | Translation | AT4G22930.1 | PYR4, DHOASE | Pyrimidin 4 |
|  |  | 3,5 | Cleavage | AT4G34250.1 | KCS16 | 3-ketoacyl-CoA synthase 16 |
| miR8175 | P-L  CDP-P  CDP-L | 3,5 | Cleavage | AT5G28641.1 |  | Transposable element gene |
|  |  | 3,5 | Cleavage | AT1G22040.1 |  | Galactose oxidase/kelch repeat superfamily protein |
|  |  | 3,5 | Cleavage | AT5G47500.1 |  | Pectin lyase-like superfamily protein |
|  |  | 3,5 | Cleavage | AT3G05520.2 |  | Subunits of heterodimeric actin Filament capping protein Capz superfamily |
|  |  | 3,5  3,5 | Cleavage  Cleavage | AT5G36890.1 AT5G36890.2 | BGLU42 | Beta glucosidase 42 |
|  |  | 3,5 | Translation | AT5G55340.1 | MBOAT | Membrane bound O-acyl transferase family protein |
|  |  | 4,0  4,0 | Translation  Translation | AT3G63220.1  AT3G63220.2 |  | Galactose oxidase/kelch repeat superfamily protein |
|  |  | 4,0 | Translation | AT5G02030.1 | LSN, PNY, HB-6, BLR, RPL, BLH9, VAN | POX (plant homeobox) family protein |
|  |  | 4,0 | Cleavage | AT2G25680.1 | MOT1 | Molybdate transporter 1 |
|  |  | 4,0  4,0  4,0  4,0 | Cleavage  Cleavage  Cleavage  Cleavage | AT1G10740.1  AT1G10740.1  AT1G10740.1  AT1G10740.1 |  | Alpha/beta-Hydrolases superfamily protein |
| miR8177 | CDP-P | 2,0 | Cleavage | AT2G01560.1 |  | Plant protein 1589 of unknown function |
|  |  | 2,5  2,5  3,0 | Cleavage  Cleavage  Cleavage | AT2G03650.1  AT3G42803.1  AT3G30814.1 |  | Transposable element gene |
|  |  | 3,0 | Cleavage | AT3G23050.2 | IAA7, AXR2 | Indole-3-acetic acid 7 |
|  |  | 3,5  3,5 | Cleavage  Cleavage | AT5G18500.1  AT5G18500.2 |  | Protein kinase superfamily protein |
|  |  | 3,5 | Cleavage | AT5G21960.1 |  | Integrase-type DNA-binding superfamily protein |
|  |  | 3,5  3,5  3,5  3,5 | Cleavage  Cleavage  Cleavage  Cleavage | AT1G14570.1  AT1G14570.2  AT1G14570.3  AT1G14570.4 |  | UBX domain-containing protein |
|  |  | 3,5 | Cleavage | AT1G31430.1 |  | Pentatricopeptide repeat (PPR-like) superfamily protein |
|  |  | 3,5 | Cleavage | AT5G08200.1 |  | Peptidoglycan-binding LysM domain-containing protein |
|  |  | 3,5 | Cleavage | AT1G14000.1 | VIK | VH1-interacting kinase |
|  |  | 3,5  3,5 | Cleavage  Cleavage | AT5G45500.1  AT5G45500.2 |  | RNI-like superfamily protein |

Tab. 4 Enzyme mixture composition for *A. thaliana* mesophilic protoplasts isolation, pH 5.6 (Chupeau et al. 2013)

| **Components** | **mg/ 1000ml** |
| --- | --- |
| KNO_3_ (POCH) | 250 |
| CaCl_2_ (POCH) | 11,3 |
| MgSO_4_ x 7H_2_O (POCH) | 25 |
| (NH_2_)_4_SO_4_ (POCH) | 13,4 |
| NaH_2_PO_4_ (POCH) | 15 |
| Gamborg B5 Microsoles (Duchefa) | 1000 |
| Inositol (Duchefa) | 100 |
| Panthotenate Ca (Sigma-Aldrich) | 1 |
| Biotin (Duchefa) | 0,01 |
| Niacin (Duchefa) | 1 |
| Pyridoxin (Duchefa) | 1 |
| Thiamin (Duchefa) | 1 |
| Glucose (Sigma-Aldrich) | 45 000 |
| Glycine (Sigma-Aldrich) | 25 000 |
| MES (2-(N-morpholino)ethanesulfonic acid) (Sigma-Aldrich) | 700 |
| Macerozyme (Serva) | 1 000 |
| Cellulase Onozuka R-10 (Yakult) | 1 000 |

Tab. 5 Media compositions for *A. thaliana* mesophilic protoplast culture (mg/L), pH 5,6 (Chupeau et al. 2013)

| **Components** | **mg/1000ml** |
| --- | --- |
| KNO_3_ (POCH) | 505 |
| NH_2_NO_3_ (POCH) | 160 |
| CaCl_2_ (POCH) | 334,2 |
| MgSO_4_ x 7H_2_O (POCH) | 370 |
| KH_2_PO_4_ (POCH) | 170 |
| (NH_4_)_5_[Fe(C_6_H_4_O_7_)_2_] (POCH)***** | 30 |
| KI (POCH) | 0,01 |
| H_3_BO_3_ (POCH) | 1 |
| MnSO_4_ x 4H_2_O (POCH) | 0,1 |
| ZnSO_4_ x 7H_2_O (POCH) | 1 |
| CuSO_4_ x 5H_2_O (POCH) | 0,03 |
| AlCl_3_ (POCH) | 0,03 |
| NiCl_2_ x 6H_2_O (POCH) | 0,03 |
| Gamborg B5 vitamins (Duchefa) | 1031 |
| Panthotenate Ca (Sigma-Aldrich) | 1 |
| Biotin (Duchefa) | 0,01 |
| Folic acid (Duchefa) | 0,2 |
| Glucose (Sigma-Aldrich) | 40000 |
| Mannitol (Sigma-Aldrich) | 60000 |
| 2,4-dichlorophenoxyacetic acid (Duchefa) | 1 |
| Thidiazuron-TZ (Duchefa) | 0,022 |
| MES (2-(N-morpholino)ethanesulfonic acid) (Sigma-Aldrich) | 700 |

Tab. 6 Primers used for mRNAs

| **mRNA** | **Forward** | **Reverse** |
| --- | --- | --- |
| TCP4 | TCGCTCCTCCTACTCCTTCA | GGTGGAGATGGATTGGTGAT |
| RGD3 | GCGGACATTGGTAATGAGGA | TACACTTCCATGGCGGATCT |
| CUC1 | CAACGGGACTGAGAACGAAC | CGTCAAGGCGATACTCATGC |
| CUC2 | TCCTGTTTCTCCACTGTCCC | TCGATGCAGAAGACGATCCA |
| STM | GCTCGTCAACAACTGCTTGA | AATGGTGAGGATGTGTTGCG |
